# An updated phylogeny of Boraginales based on the Angiosperms353 probe set: a roadmap for understanding morphological evolution

**DOI:** 10.1093/aob/mcaf061

**Published:** 2025-04-10

**Authors:** Maria-Anna Vasile, Tim Böhnert, Julius Jeiter, Domingos Cardoso, Peter W Moonlight, Maximilian Weigend

**Affiliations:** Bonn Institute of Organismic Biology, University of Bonn, Bonn, Germany; Bonn Institute of Organismic Biology, University of Bonn, Bonn, Germany; Chair of Botany, Faculty of Biology, TUD Dresden University of Technology, Dresden, Germany; Instituto de Pesquisas Jardim Botânico do Rio de Janeiro, Rio de Janeiro, Brazil; Instituto de Biologia, Universidade Federal da Bahia, Salvador, Bahia, Brazil; Botany, School of Natural Sciences, Trinity College Dublin, Dublin, Ireland; Bonn Institute of Organismic Biology, University of Bonn, Bonn, Germany

**Keywords:** Asterids, Hyb-Seq, lamiids, molecular phylogenetics, systematics, target sequence capture, Tree of Life, internal ovary architecture, placentation, fruit evolution

## Abstract

**Background and Aims:**

Boraginales, a subcosmopolitan order with ~2700 species in currently 11 families, has seen fluctuating classification at the family and generic levels. Gynoecium and fruit morphology have been pivotal in morphological classifications, but a systematic evaluation based on anatomical and ontogenetic data has not been attempted.

**Methods:**

We revisited the phylogenetic relationships in Boraginales by analysing 162 newly sequenced samples using the Angiosperms353 probe set, together with existing data from 88 samples. Our sampling covers >80 % of the genera and all currently and previously recognized families. A morphological assessment of taxonomically important gynoecial and fruit characters was done to guide our proposed family-level classification.

**Key Results:**

The phylogenies are largely consistent with previous phylogenetic studies, with better resolution overall and support from both datasets (exons, supercontigs) and all analyses implemented (coalescence, ASTRAL-Pro3, concatenation). The placement of Hydrophyllaceae as nested within Namaceae is a novel result, while the placement of the parasitic Lennoaceae as nested within Ehretiaceae is confirmed with maximum confidence. The placement of Codonaceae remains ambiguous: retrieved within Boraginales I, as sister to the clade comprising Wellstediaceae and Boraginaceae based on exons, and as sister branch to all remaining Boraginales II based on supercontigs. We propose the recognition of a total of nine families in Boraginales. Tribal relationships in Boraginaceae subfam. Cynoglossoideae are fully resolved for the first time. Our data show that shifts in placentation and ovule number are instrumental for the arisal of complex internal ovary architectures.

**Conclusions:**

The results highlight the effectiveness of the Angiosperms353 probe set for understanding the evolutionary history of Boraginales and pave the way for resolving remaining unresolved nodes and taxonomic issues. Complex modifications of bicarpellate ovaries led to an extreme and lineage-specific diversification of fruits that probably played a crucial role in Boraginales macroevolution. Combining this highly resolved phylogeny with future critical morphological analyses promises a deep understanding of evolutionary trajectories in Boraginales.

## INTRODUCTION

**Fig. 1.**
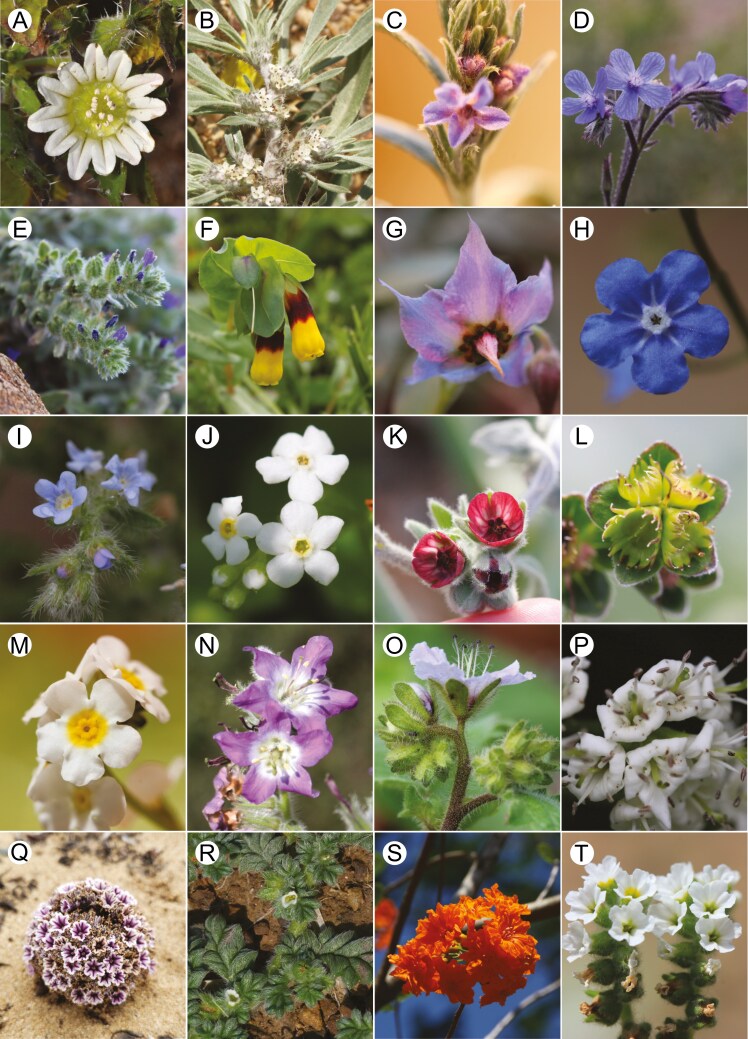
Morphological diversity in Boraginales. (A) Codonaceae (*Codon royenii* L.). (B) Wellstediaceae (*Wellstedia* cf. *dinteri* Pilg, Ugor via iNaturalist https://www.inaturalist.org/observations/194363877 CC-BY-NC 4.0 https://creativecommons.org/licenses/by-nc/4.0/, Namibia). (C–M) Boraginaceae. (C) Echiochiloideae (*Echiochilon fruticosum* Desf., xx-0-BONN-15043). (D) Boragineae (*Anchusa azurea* Mill., Greece). (E) Lithospermeae (*Echium arenarium* Guss., Greece). (F) Lithospermeae (*Cerinthe major* L., Greece). (G) Trichodesmeae (*Trichodesma boissieri* Post, JO-0-BONN-35154). (H) Omphalodeae [*Omphalodes nitida* (Willd.) Hoffmanns. & Link, xx-0-BONN-14659]. (I) Rochelieae, Eritrichiinae [*Lappula consanguinea* (Fisch. & C.A.Mey.) Gürke., xx-0-BONN-39580]. (J) Myosotideae (*Trigonotis formosana* Hayata, xx-0-BONN- 29917). (K) Cynoglossinae (*Cynoglossum*  *columnae* Mill., Greece). (L) Cynoglossinae [*Lindelofia longiflora* (DC.) Baill. xx-0-BONN-27824]. (M) Amsinckiinae [*Plagiobothrys chorisianus* var. *hickmanii* (Greene) I.M.Johnst., xx-0-BONN-27824]. (N) Namaceae [*Wigandia urens* (Ruiz & Pav.) Kunth, Colombia]. (O) Hydrophyllaceae (*Phacelia bolanderi* A.Grey, xx-0-BONN-40976). (P) Ehretiaceae (*Ehretia dicksonii* Hance, xx-0-BONN-12602). (Q) Lennoaceae (*Pholisma arenarium* Nutt., photo and collection: B. Lee & D. Grossenbacher 1, California, USA). (R) Coldeniaceae (*Coldenia procumbens* L., Radha Veach via iNaturalist https://www.inaturalist.org/observations/149752935 CC-BY-NC 4.0 https://creativecommons.org/licenses/by-nc/4.0/, India). (S) Cordiaceae (*Cordia sebestena* L., photo: M. Ackermann, Cuba). (T) Heliotropiaceae (*Heliotropium europaeum* L., DE-0-BONN-26153).

Boraginales are a medium-sized order of angiosperms within lamiids (Zhang *et al.*, 2020b), sister to Gentianales (Zuntini *et al.*, 2024). Currently, 11 families with ca. 133 genera and ca. 2700 species are recognized (Chacón *et al.*, 2016; Luebert *et al.*, 2016). Boraginales are subcosmopolitan in distribution, predominantly found in seasonally arid habitats across temperate and tropical regions (Weigend *et al.*, 2014). The stem node of Boraginales is estimated at ca. 109 Ma, probably originating in West Gondwana during the Early Cretaceous. The Boraginales contain two major clades that diverged in the Late Cretaceous, probably after West Gondwana vicariance (Luebert *et al.*, 2017). These clades were identified using chloroplast data (Weigend *et al.*, 2014) and are known as Boraginales I and II. Boraginales show largely conserved floral organization (i.e. merosity of the different organ whorls and their arrangement; Jeiter, 2020) with essentially actinomorphic, tetracyclic, sympetalous and pentamerous flowers that are usually arranged in scorpioid cymes (Fig. 1; Buys and Hilger, 2003).

The outstanding feature of Boraginales is their diverse fruit morphology, which has been central to their systematics, ranging from dehiscent capsules to various indehiscent fruit types. The dispersal units vary widely due to different degrees of seed and pericarp integration (e.g. mericarpids, endomericarpids; see Box 1). Boraginales fruits have attracted significant attention from researchers both in systematics (Gottschling and Hilger, 2004*a*; Selvi *et al.*, 2006; Weigend *et al.*, 2009, 2010, 2013, 2014; Weigend and Hilger, 2010; Hasenstab-Lehman and Simpson, 2012; Gottschling *et al.*, 2014*a*; Diane *et al.*, 2016; Luebert *et al.*, 2016; Otero *et al.*, 2019*a*; Frohlich *et al.*, 2022) and comparative morphology (Hilger, 1987, 1992, 2014; Di Fulvio *et al.*, 1997, 1999; Gottschling *et al.*, 2014*b*; Jeiter *et al.*, 2016, 2018, 2023; Heigl *et al.*, 2020; Vasile *et al.*, 2021, 2022). However, these morphological studies still only cover a proportion of the overall fruit diversity in the order. This fact, combined with limited resolution and support in order-wide phylogenies, has hampered a more comprehensive understanding of the evolution of fruit characters.

Box 1.

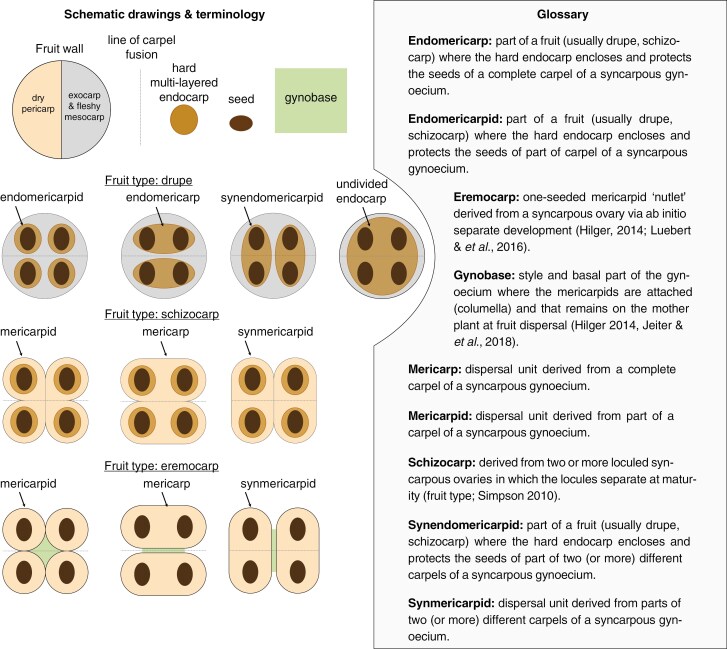

Boraginales classification has remained in flux, due largely to different interpretations of fruit morphology and, more recently, various molecular phylogenies with different sampling and resolution. Boraginales have been treated as a family or order and consequently subdivided in a variety of either subfamilies or families (de Candolle, 1845, 1846; Bentham and Hooker, 1876; Gürke, 1893; Brand, 1913; Takhtajan, 1987, 1997; Bittrich, 2016; Diane *et al.*, 2016; Gottschling *et al.*, 2016; Hilger and Weigend, 2016; Hofmann *et al.*, 2016; Weigend and Hilger, 2016; Weigend *et al.*, 2016). APG IV (2016) recognizes Boraginales as an order but does not follow the classification of Luebert *et al.* (2016), who formalized the separation into 11 families based on molecular and morphological characters; rather, it recognizes only a single family, Boraginaceae.

Two main clades have been identified based on chloroplast data (Gottschling *et al.*, 2001; Weigend *et al.*, 2014): Boraginales I comprising Codonaceae, Wellstediaceae and Boraginaceae, and Boraginales II including Hydrophyllaceae, Namaceae, Heliotropiaceae, Lennoaceae, Ehretiaceae, Cordiaceae, Coldeniaceae and Hoplestigmataceae. Each clade consists of basal grades of two families with dehiscent fruits (Codonaceae + Wellstediaceae; Hydrophyllaceae + Namaceae). The remaining families have only four ovules, in indehiscent fruits (drupes, eremocarps or schizocarps; Box 1; Lennoaceae are one of the few exceptions).

Boraginales I was resolved by Weigend *et al.* (2014) with monogeneric Codonaceae placed sister to monogeneric Wellstediaceae and highly diverse Boraginaceae. Historically, Codonaceae were included in Hydrophyllacae *s.l.* (*sensu*  Brand, 1913) based on gynoecium and fruit characters, such as the bifid style, multi-seeded capsules and intrusive parietal placentation (Brand, 1913; Weigend and Hilger, 2016). Wellstediaceae, encompassing about six species, has been historically placed in or close to Boraginaceae, but with a degree of uncertainty due to its divergent flower and fruit morphology (Hilger and Weigend, 2016). Boraginaceae stands out as the most species-rich family in Boraginales, comprising the majority of genera (~90) and about 1600–1700 species (Luebert *et al.*, 2016). An updated infrafamilial classification for Boraginaceae was provided by Chacόn *et al.* (2016) based on Sanger sequencing, but relationships along the backbone remained largely unresolved. Chacόn *et al.* (2016) accept three subfamilies, namely Echiochiloideae, Boraginoideae and Cynoglossoideae, the last being the most species rich. Nuclear alignments (ITS1 and ITS2 regions) resulted in a poorly resolved backbone, especially in Cynoglossoideae (Chacón *et al.*, 2016). Chloroplast data in turn yielded an overall better resolved backbone at subfamily level (i.e. the sister relationship of Cynoglossoideae to Boraginoideae), as well as at tribal and subtribal levels (Chacón *et al.*, 2016). However, our understanding of relationships within Boraginaceae remains incomplete (see previous studies; Hilger *et al.*, 2004; Selvi *et al.*, 2006; Weigend *et al.*, 2009, 2014; Hasenstab-Lehman and Simpson, 2012; Nazaire and Hufford, 2012; Chacón *et al.*, 2016, 2019; Otero *et al.*, 2019*a*), with some clades consistently recovered as unresolved in phylogenetic analyses (e.g. tribal and subtribal classification of Cynoglossoideae) and some generic limits entirely obscure (esp. *Cynoglossum* L.; Pourghorban *et al.*, 2020, 2023).

Several familial relationships within Boraginales II remain unresolved (Luebert *et al.*, 2016). Namaceae have long been treated as clade II of Hydrophyllaceae *s.l.* (*sensu*  Hofmann *et al.*, 2016) and have been resolved as either the sister group of the Hydrophyllaceae I clade or nested within it forming a paraphyletic grade of the remaining Boraginales II based on chloroplast data, but with low or moderate support (Ferguson, 1998; Nazaire and Hufford, 2012; Refulio‐Rodriguez and Olmstead, 2014; Weigend *et al.*, 2014). Hydrophyllaceae and Namaceae were only recently resolved as sister groups with maximum support based on whole transcriptome sequence data, in line with the classification of Luerbert *et al*. (2016), but with very limited sampling (excluding the largest genus *Nama* L., Zhang *et al.*, 2020b). Until now, neither molecular nor morphological data satisfactorily support the current classification. Furthermore, the position of Heliotropiaceae is also ambiguous. Based on either nuclear or chloroplast markers, Heliotropiaceae were repeatedly retrieved as a weakly supported sister to a clade comprising Lennoaceae, Ehretiaceae, Coldeniaceae, Hoplestigmataceae and Cordiaceae (Gottschling *et al.*, 2001; Refulio‐Rodriguez and Olmstead, 2014; Weigend *et al.*, 2014). However, in Zhang *et al.*, (2020b), Heliotropiaceae were retrieved as sister to the Cordiaceae + Coldeniaceae, while Ehretiaceae were retrieved as sister to Heliotropiaceae + (Cordiaceae + Coldeniaceae), albeit based on a very limited sampling. The placement of holoparasitic Lennoaceae had been doubtful since its description by Solms-Laubach (1870) and it has been consistently recognized at the family level due to its highly derived morphology (Bittrich, 2016; Luebert *et al.*, 2016). Based on molecular data, Lennoaceae were recognized as closely related to Boraginales II (Smith *et al.*, 2000) and retrieved either as sister to Ehretiaceae (Gottschling *et al.*, 2001) or nested in Ehretiaceae (Nazaire and Hufford, 2012; Weigend *et al.*, 2014; Gottschling *et al.*, 2014a; Zhang *et al.*, 2020b). Infrafamilial classification of all Boraginales II families has been addressed in several studies with variable degrees of resolution (Hydrophyllaceae and Namaceae: Ferguson, 1998; Walden *et al.*, 2014; Vasile *et al.*, 2020; Heliotropiaceae: Hilger and Diane, 2003; Luebert *et al.*, 2011*a*; Frohlich *et al.*, 2022; Cordiaceae and Ehretiaceae: Gottschling *et al.*, 2005, 2014*a*), yet many open questions remain regarding inter- and infrafamilial relationships.

In recent years target enrichment has become important for reconstructing phylogenies in numerous clades within the angiosperms (Lee *et al.*, 2021; [Bibr CIT0113][Bibr CIT0073]). It remains almost completely unexplored in Boraginales and has only been used at the genus or family level thus far (*Lithospermum* L. Cohen, 2022; Boraginaceae Cohen, 2025; *Myosotis* L. Meudt *et al.*, 2025). Here, we aim to provide new insights into the evolution of Boraginales. To achieve this, we use the Angiosperms353 (Johnson *et al.*, 2019) universal target enrichment probe set at different taxonomic levels to evaluate previously published hypotheses for relationships of Boraginales. Our goals are to: (1) resolve the backbone of Boraginales up to subtribal level; (2) provide a basis for an updated classification at the family level that accommodates most of the morphological heterogeneity of the order and renders a solid foundation for future studies; and (3) in the light of a well-resolved phylogeny, provide a hypothesis of fruit evolution in Boraginales.

## MATERIALS AND METHODS

### Taxon sampling

This study comprises 251 accessions of Boraginales, representing all 11 currently or previously recognized families (*sensu*  Luebert *et al.*, 2016) and 111 genera (83 % of the overall genus diversity; Table 1). Species names follow Plants of the World Online (POWO, https://powo.science.kew.org, accessed 23 December 2024). In total, 162 new sequence datasets were generated for this study. Leaf tissue preserved in silica gel, mainly collected from the field or botanical gardens, as well as tissue samples from herbarium specimens, were utilized. The following herbaria provided material for this study: AA, B, BM, BONN, E, EIF, F, FI, GZU, HAJB, HUH, JE, K, LE, M, NSK, NY, PERTH, RB, SGO, TARI, TUH, UPS and USM. Sequences of the remaining 88 Boraginales samples as well as four additional outgroup samples from the closely related orders Solanales (Solanaceae and Convolvulaceae) and Gentianales (Gentianaceae and Rubiaceae) were taken from the Plant and Fungal Tree of Life project (Baker *et al.*, 2022). All voucher information can be found in the Supplementary Data (Table S1).

**Table 1.** Taxon coverage at the genus level and number of accessions per taxonomic group. Number of accepted genera is approximately given according to Luebert *et al.* (2016), Chacón *et al.* (2016) and POWO. The single asterisk indicates that only two genera are currently accepted within Cynoglossinae: *Microparacaryum* (Popov ex Riedl) Hilger & Podlech and *Cynoglossum* L*. s.l.* The double asterisk indicates that according to Jepson eFlora, Revision 4. 2021, *Turricula parryi* (A.Gray) J.F.Macbr. is treated as *Eriodictyon parryi* (A.Gray) Greene. The monotypic genera *Nogalia* Verdc. and *Rotula* Lour. have not been considered as accepted, following Luebert *et al.* (2016).

Family	Subfamily	Tribe	Subtribe	No. of accepted genera	No. of genera in this study	No. of accessions
Boraginaceae	Echiochiloideae	Echiochileae		3	2	3
Boraginaceae	Boraginoideae	Boragineae	Boragininae	15	12	14
Boraginaceae	Boraginoideae	Boragineae	Mortziinae	2	2	3
Boraginaceae	Boraginoideae	Lithospermeae		25	20	29
Boraginaceae	Cynoglossoideae	Trichodesmeae		3	3	5
Boraginaceae	Cynoglossoideae	Lasiocaryeae		3	3	4
Boraginaceae	Cynoglossoideae	Asperugeae		4	4	7
Boraginaceae	Cynoglossoideae	Omphalodeae		6	4	7
Boraginaceae	Cynoglossoideae	Rochelieae	Eritrichiinae	7	8	13
Boraginaceae	Cynoglossoideae	Rochelieae	Heterocaryinae	1	1	2
Boraginaceae	Cynoglossoideae	Craniospermeae		1	1	2
Boraginaceae	Cynoglossoideae	Myosotideae		4 (5)	4	12
Boraginaceae	Cynoglossoideae	Cynoglosseae	Cynoglossinae	2*	2	22
Boraginaceae	Cynoglossoideae	Cynoglosseae	Bothriosperminae	5	2	3
Boraginaceae	Cynoglossoideae	Cynoglosseae	Microulinae	3	2	4
Boraginaceae	Cynoglossoideae	Cynoglosseae	Amsinckiinae	ca. 14	10	14
Codonaceae				1	1	3
Wellstediaceae				1	1	2
Hydrophyllaceae				12	9	11
Namaceae				3**	3	12
Heliotropiaceae				4	3	31
Ehretiaceae				7	7	27
Lennoaceae				2	2	3
Cordiaceae				2	2	13
Coldeniaceae				1	1	3
Hoplestigmataceae				1	1	2

### DNA extraction, library preparation and sequencing

Genomic DNA was extracted from dried tissue using a CTAB protocol (Doyle and Doyle, 1987), modified by increasing the incubation time to 90 min, followed by an initial quality control using electrophoresis on 1 % agarose gels using Lonza GelStar Nucleic Acid Gel Stain (100×; Lonza Bioscience, Basel, Switzerland) and lambda DNA (250 μg, Promega, Madison, WI, USA). DNA concentration was further quantified using a Quantus Fluorometer (Promega) based on which 162 samples with ~1 μg genomic DNA per sample were prepared. Samples were sent to a sequencing service for library preparation and subsequent sequencing (LGC Genomics GmbH, Biosearch Technologies, Berlin, Germany). See Acuña-Castillo *et al.* (2024) for a detailed description of the library preparation. In brief, preparation of indexed Illumina libraries was carried out using Encore Rapid DR Multiplex System 1–96 (Tecan Group Ltd, Männedorf, Switzerland). Each library was amplified individually over 10 cycles with MyTaq HS Red Mix (Meridian Bioscience, Memphis, TN, USA) and standard Illumina primers, in a 30 µL reaction volume. Enrichment was performed using the ‘myBaits Expert Angiosperms-353’ (Arbor Biosciences, Cambridge, MA, USA) following the plant material protocol, but again using the MyTaq HS Red Mix to prevent PCR bias during this step. DNA library quality control was performed via an Agilent Fragment Analyzer (Agilent, Santa Clara, CA, USA) and Qubit 2.0 Fluorometer (Life Technologies, CA, USA). An Illumina NextSeq 500/550 v2 (Illumina, San Diego, CA, USA) was used for sequencing, generating short paired-end reads of 150 bp with an aimed average sequencing depth of 1.6 million reads per sample. Raw sequencing data are deposited in the European Nucleotide Archive (ENA) under accession number PRJEB87169.

### Data processing and assembly

Demultiplexing of the sequenced libraries was performed with the Illumina bcl2fastq v.2.20 conversion software (Illumina). Up to two mismatches or Ns were allowed in the barcode read when the barcode distances between all libraries on the lane allowed for it. FastQC v.0.11.9 (Andrews *et al.*, 2012) was used for evaluating raw read quality, checking adapter remnants and producing FastQC reports for all FASTQ files. Sequencing reads were further processed using Trimmomatic v.0.39 (Bolger *et al.*, 2014) to remove leading and trailing low-quality bases with a Phred-scaled quality score below 20. With this setting, bases were cut when the average quality within a 4-bp sliding window dropped below 20 and reads shorter than 30 bp were excluded (LEADING: 20, TRAILING: 20, SLIDING WINDOW: 4:20, MINLEN: 30). Only cleaned reads in which both read pairs passing this quality control were kept for subsequent assembly and analyses.

For sequence assembly and extraction, the HybPiper v.2.1.1 (Johnson *et al.*, 2016) pipeline was used. For the HybPiper assembly, the mega353 target file (McLay *et al.*, 2021) was used instead of the default Angio353 target file (Johnson *et al.*, 2019). The mega353 fasta file was filtered for Boraginales (15 targets) but also for sister orders of Boraginales: Gentianales (17 targets) and Solanales (26 targets), resulting in reference sequences from 58 taxa. Those were then checked for sequences with low-complexity regions prior to the assembly, using the ‘hybpiper check_targetfile’ command and then fixed with the *fix_targetfile.py* script available with HybPiper. The trimmed paired reads were mapped to the filtered mega353 targets using the Burrows–Wheeler alignment option (Li and Durbin, 2009) and then assembled *de novo* with SPAdes (Bankevich *et al.*, 2012). Then, for each locus, exon as well as supercontig sequences (exon + intron) were recovered by providing the *--run_intronerate* flag, while running the *exonerate_hits.py* script available with HybPiper. Both exon and supercontigs were retained for downstream analyses. To summarize recovery efficiency, the *hybpiper_stats.py* script available with HybPiper was used (Supplementary Data Table S2) and visualization of the coverage was done by implementing the *gene_recovery_heatmap.py* script (Supplementary Data Fig. S1).

During the HybPiper assembly, if multiple contigs with >10× depth of coverage in a sample map to the same target gene with >75 % sequence identity, this target gene is marked for the presence of paralogues in that sample. HybPiper selects the sequence with the highest percentage identity to the reference if all competing long contigs have similar depth. The *paralog_retriever.py* script available with HybPiper was used to further investigate the distribution of paralogues within our samples. This script produces, among other files, a report table with the number of sequences for each gene and sample, along with a heatmap visualization (Supplementary Data Fig. S2). If many samples have more than one copy for several genes, it may indicate an ancient gene duplication. If one sample tends to have many copies, it may indicate polyploidy. Subsequently, three datasets resulted from sequence assembly – an exon dataset, a supercontigs dataset, and another exon dataset for which multi-copy gene regions were not removed in order to evaluate the performance of paralogue filtering by using a coalescence-based approach that is capable of accounting for multi-copy regions.

### Sequence alignment

The two datasets filtered for paralogy (exon and supercontig) as well as the multi-copy exon dataset were first aligned using MAFFT v.7.515 (Katoh and Standley, 2013) with default parameters. The individual gene alignments produced with MAFFT were further processed with CIAlign v.1.1.0 (Tumescheit *et al.*, 2022). The flags *--crop_ends* (i.e. ends of sequences were cropped when poorly aligned), *--remove_min_length* (i.e. sequences were removed if they were shorter than 50 bp, excluding gaps), *--remove_divergent_minperc* (i.e. 0.5 was the minimum proportion of positions which should be identical to the most common base in order to be preserved) and *--insertion_min_flank* (i.e. a minimum number of 5 bases on either side of an insertion is needed to classify it as an insertion) were used. The fine-tuned alignments were used on the one hand for gene tree analyses and on the other hand concatenated using the R package TOAST v.0.0.1.5000 (Transcriptome Ortholog Alignment Sequence Tools; available from www.github.com/ziyili20/TOAST) in R v.4.3.1 (R Core Team, 2023) for concatenated analyses. TOAST utilizes the orthologue searches based on the software benchmarking Universal Single-Copy Orthologs (BUSCO) to assemble multiple sequence alignments of orthologous loci. In addition to the construction of concatenated alignments, TOAST generated a partition block and assessed overall missing data patterns across the alignments. Finally, five individual sets of alignments were generated for downstream phylogenetic analyses: individual gene alignments for exons and supercontigs, respective concatenated alignments, as well as individual exon alignments with all multi-copy regions.

### Phylogenetic reconstruction

Multi-locus species tree estimation and locus concatenation approaches were implemented in this study. For the concatenated alignments of both exons and supercontigs, a partitioned analysis was conducted in IQ-TREE v.2.0.3 (Nguyen *et al.*, 2015) in order to reduce statistical inconsistency. Roch and Steel (2015) demonstrated that concatenation using unpartitioned maximum likelihood analysis can be statistically inconsistent and even positively misleading in the presence of incomplete lineage sorting (ILS). Therefore, the analysis was performed with ModelFinder (Kalyaanamoorthy *et al.*, 2017) identifying the best model + partitioning strategy (MFP + MERGE) and using an ultrafast bootstrap option (Hoang *et al.*, 2018) to generate 1000 bootstrap replicates. However, concatenation methods can be inconsistent under ILS and may estimate incorrect species trees as the number of genes increases (Xiong *et al.*, 2022), even with high support under some conditions (Warnow, 2015). For these reasons, more attention is given to the multi-locus species tree estimations. For these analyses the individual locus alignments of the exons and supercontigs were used as input in IQ-TREE v.2.0.3 (Nguyen *et al.*, 2015) which provides the option to compute individual locus trees and simultaneously run ModelFinder to find the best fitting model for each locus. Again, ultrafast bootstrapping was used to generate 1000 bootstrap replicates for each gene tree. To account for ILS, an unrooted species tree was estimated with ASTRAL-III v.5.7.8 (Zhang *et al.*, 2018) and the branches on the species tree were scored using local posterior probabilities. Additionally, the R package AstralPlane v.0.1.1 (https://github.com/chutter/AstralPlane) was used to estimate tree discordance and plot the quartet frequencies as pie charts.

According to Zhang *et al.*, (2020b), Boraginales II have a whole genome duplication (WGD) at their crown node (Ks value = 0.73, divergence time = 61.86 Ma), while one WGD event is also detected for Boraginaceae (Ks value = 0.87, divergence time = 65.81 Ma) and one for Codonaceae (Ks value = 0.58, divergence time = 41.38 Ma). Ufimov *et al.* (2022) suggest that deleting the loci flagged as paralogous from the analysis is inappropriate for taxa with a WGD event in their relatively recent history, as deleting loci with paralogue warnings will cause substantial data loss. Therefore, for evaluating the effect of the paralogous genes on the phylogenetic reconstruction, we used ASTRAL-Pro3 v.1.19.3.5 (Zhang *et al.*, 2020a; Zhang and Mirarab, 2022), a tool which allows the use of multi-copy genes and utilizes a measure of quartet similarity between single-copy and multi-copy trees accounting for orthology and paralogy. Trees were rooted with Solanales, since the order was repeatedly retrieved as sister to Gentianales + Boraginales (Zhang *et al.*, 2020b; Zuntini *et al.*, 2024), and visualized in FigTree v.1.4.4 (http://tree.bio.ed.ac.uk/software/figtree/).

### Morphological assessment

Given the importance of fruit and gynoecium characters to current and previous family-level classifications in Boraginales (Bentham and Hooker, 1876; Gürke, 1893; Luebert *et al.*, 2016), we focused on collecting data in order to summarize the major fruit and gynoecium types across the major clades identified in our phylogenetic analyses, and to guide our proposed family-level classification. Specifically, we focused on the following characters: placentation and ovary subdivision, presence/absence of dehiscence and ovule/seed number (and their integration in the pericarp) to give an order- and family-level overview of gynoecium development and internal ovary structure summarized as schematic drawings. We refrained from carrying out ancestral state reconstructions because our aim was not to look at evolutionary trends in these characters. Interpretation of the detailed evaluation of the bewildering array of character states found in Boraginales II (Hilger, 1987, 1992; Di Fulvio *et al.*, 1997, 1999; Gottschling and Hilger, 2004*b*; Gottschling *et al.*, 2014*b*; Jeiter *et al.*, 2016, 2018, 2023; Heigl *et al.*, 2020; Vasile *et al.*, 2021, 2022) is clearly beyond the scope of the present study, but will be addressed in future publications.

Morphological and developmental data on the gynoecium and fruit with a focus on the above-mentioned characters were taken from the literature (Hilger, 1987, 1989, 1992, 2014; Di Fulvio *et al.*, 1997, 1999; Diane *et al.*, 2002; Gottschling and Hilger, 2004*b*; Gottschling *et al.*, 2014*b*; Jeiter *et al.*, 2016, 2018, 2023; Heigl *et al.*, 2020; Vasile *et al.*, 2021, 2022). Additionally, original data were obtained using scanning electron microscopy (SEM) for 80 species and with micro-computed X-ray tomography (μCT) for 91 species. Sample preparation, scanning and data processing followed Vasile *et al.* (2022). Scans were performed at the Zoological Research Museum Alexander König in Bonn, at Naturkunde Museum Stuttgart and at Bonn Institute of Organismic Biology, Animal Diversity Section. Scanner settings and reconstruction related information can be found in the Supplementary Data (Table S3). Image stack visualization and segmentation of structures (ovules/seeds and placentae) were done in Dragonfly v.2021.3 and v.2022.1 (Object Research Systems, Montreal, Canada).

## RESULTS

### Sequence recovery

Among the 255 samples used to reconstruct the Boraginales phylogeny, including the four samples used as outgroups, the sequence data had an average of 6.1 × 10^6^ raw reads (min. 1.05 × 10^5^; max. 10.4 × 10^7^), while the average mapped reads to the target loci were 9.7 × 10^5^. Target gene recovery ranged from 151 in *Hoplestigma klaineanum* Pierre to all 351 genes (three samples, see Supplementary Data Table S2, Fig. S1). The mean gene recovery per sample was 328 genes. *Hoplestigma klaineanum* was initially included in the phylogenetic reconstruction and was retrieved as sister to *Hoplestigma pierreanum* Gilg. but was excluded from the final analyses due to the large number of missing gene regions. For downstream analyses, a set of 349 genes were used, as two genes were excluded due to missing data. The evaluation of paralogy yielded that of the 351 Angiosperm genes recovered, 274 had paralogue warnings for at least one of the 255 samples, but only 38 genes had paralogue warnings for at least 10 % of the samples. The distribution of paralogues in our data ranged from 0 to 91 (the latter in *Tiquilia tacnensis* A.T.Richardson) paralogues per sample with most of samples having low paralogue representation (average of 14 paralogue warnings). There were no distinct patterns regarding the distribution of paralogues across clades.

### Phylogenetic relationships

**Fig. 2.**
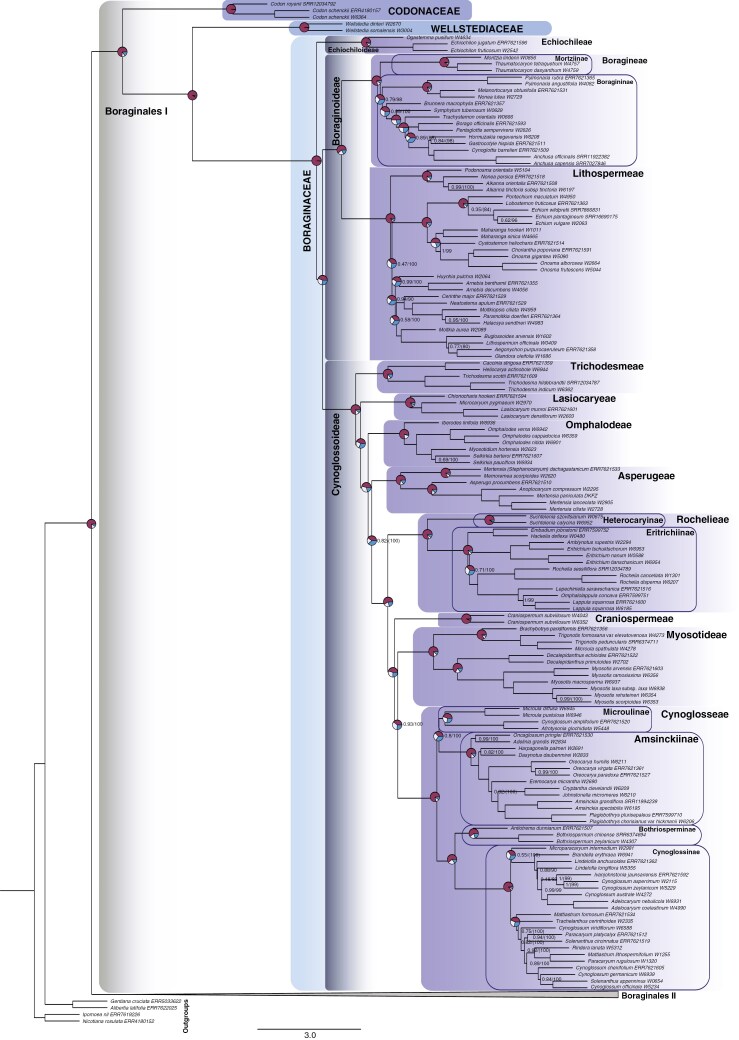
Phylogenetic relationships within Boraginales I. ASTRAL-III species tree (MuLo-Exn) generated using the retrieved exonic regions of 353 nuclear loci from the Angiosperms353 probe set. Within Boraginaceae the subfamilies, tribes and subtribes are annotated with frames of different colour depending on the taxonomic rank. Quartet score pie charts are shown on the major backbone nodes. Numerical values denote the local posterior probability (LPP) for MuLo-Exn (left) and the ultrafast bootstrap approximation (UFBoot) from the concatenation analysis (ConcaExn; right). UFBoot values in parentheses indicate incongruences in the topology between the MuLo-Exn and the ConcaExn tree. Branches without support values indicate full support (LPP/UFBoot = 1/100). Scale bar for branches is in coalescent units (CUs).

**Fig. 3.**
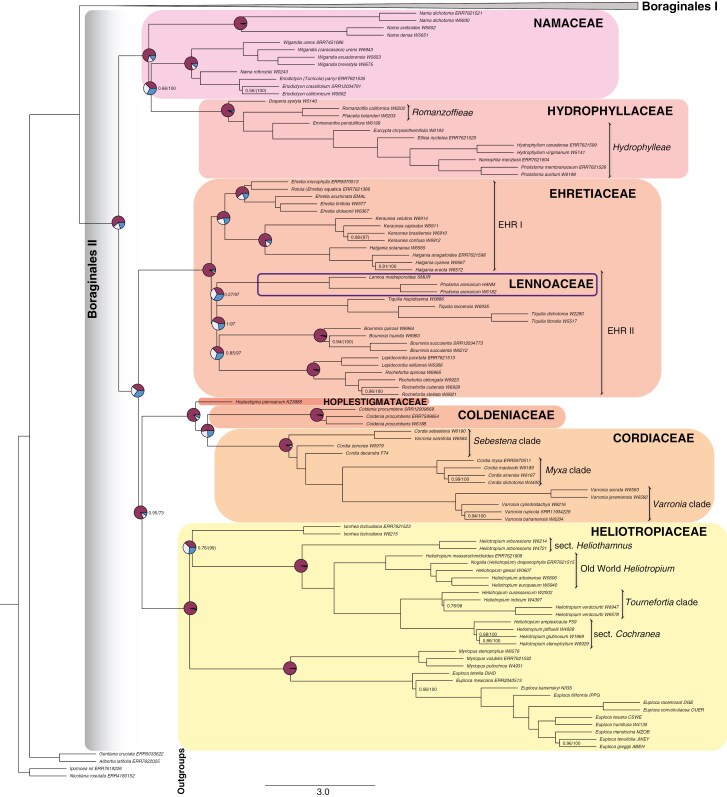
Phylogenetic relationships within Boraginales II. ASTRAL-III species tree (MuLo-Exn) generated using the retrieved exonic regions of 353 nuclear loci from the Angiosperms353 probe set. Families are annotated in different colour, while Lennoaceae is shown with a line box. Quartet score pie charts are shown on the major backbone nodes. Numerical values denote the local posterior probability (LPP) for MuLo-Exn (left), and the ultrafast bootstrap approximation (UFBoot) from the concatenation analysis (ConcaExn; right). UFBoot values in parentheses indicate incongruences in the topology between the MuLo-Exn and the ConcaExn tree. Branches without support values indicate full support (LPP/UFBoot = 1/100). Scale bar for branches is in coalescent units (CUs).

**Fig. 4.**
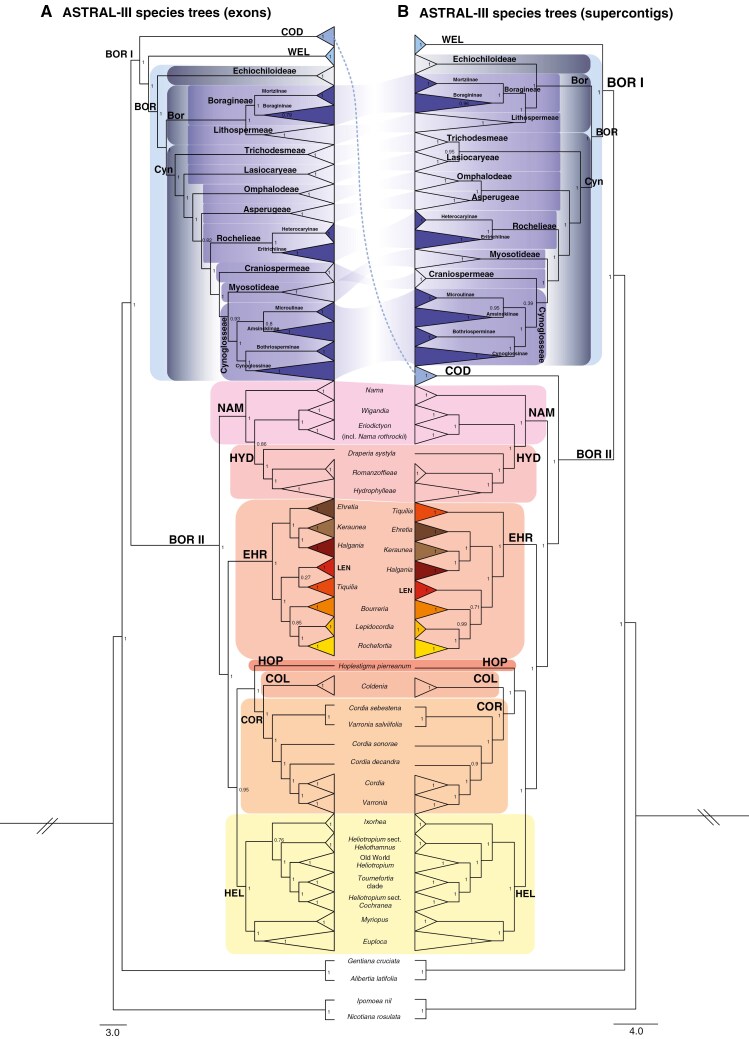
Cophylo plot showing a comparison between MuLo-Exn (A) and MuLo-SupC (B) species tree inferred using IQ-TREE. The clades Boraginales I (BOR I) and II (BOR II) are indicated. Families – Codonaceae (COD), Wellstediaceae (WEL), Boraginaceae (BOR), Namaceae (NAM), Hydrophyllaceae (HYD), Ehretiaceae (EHR), Lennoaceae (LEN), Hoplestigmataceae (HOP), Coldeniaceae (COL), Cordiaceae (COR) and Heliotropiaceae (HEL) – are shown in different colour. Within Boraginaceae the subfamilies, tribes and subtribes are also annotated with frames of different colour depending on the taxonomic rank. Numerical values denote the local posterior probability (LPP). Scale bar for branches is in coalescent units (CUs).

In total five trees were reconstructed for this study. Two multi-locus species trees were estimated with ASTRAL-III, one based on the exon alignments (hereafter MuLo-Exn; Figs 2–4) and one based on the supercontig alignments (hereafter MuLo-SupC; Fig. 4 and Supplementary Data Fig. S3). Two concatenated maximum likelihood trees were constructed, one using the exon alignments (hereafter ConcaExn; Fig. S4), and one using the supercontig alignments (hereafter ConcaSupC; Fig. S5). Finally, a multi-locus species tree accounting for paralogy was inferred using the exon alignment containing multi-copy gene regions and ASTRAL-Pro3 (hereafter ASTRAL-Pro3; Fig. S6). Both the concatenation analysis and multi-locus species tree estimation approach yielded well-resolved trees with overall congruent topologies. However, as mentioned before, the concatenation methods can be inconsistent, which is why the results of the multi-locus species tree estimations (MuLo-Exn and MuLo-SupC) are primarily described here. Support for the species trees are given as local posterior probability (LPP), while those for the concatenation analyses are given as ultrafast bootstrap approximation (UFBoot). The detailed MuLo-SupC, ConcaExn, ConcaSupC and ASTRAL-Pro3 trees are provided as Figs S3–S6.

All trees show well-resolved phylogenetic relationships among and within all families of Boraginales. According to the Mulo-Exn tree, within Boraginales I (Fig. 2), Codonaceae is retrieved as sister to Wellstediaceae and Boraginaceae with maximum support. The infrafamilial relationships of Boraginaceae are also well resolved. Echiochiloideae is sister to Boraginoideae + Cynoglossoideae with maximum support. At a tribal level within Boraginoideae, the two tribes Boragineae and Lithospermeae are sister to each other with maximum support. At the subtribal level, Mortziinae and Boragininae of tribe Boragineae are sister groups with maximum support, but the crown node of Boragininae is not consistently well supported (LPP = 0.79, UFBoot = 98). The backbone of Cynoglossoideae is well resolved, with only a few cases where the tribal relationships are not resolved with maximum support. Trichodesmeae is successively sister to Lasiocaryeae, which is then successively sister to Omphalodeae, and Omphalodeae is the successive sister group to Asperugeae, with only the latter relationship receiving moderate support (LPP = 0.82). However, the ConcaExn retrieves Lasiocaryeae as successive to a clade comprising Omphalodeae and Asperugeae (UFBoot = 100) and these two tribes are sister to each other with maximum support. Asperugeae in the MuLo-Exn species tree (Fig. 2) and Omphalodeae + Asperugeae in the ConcaExn tree (Supplementary Data Fig. S4) are successive sister groups to the remaining Cynoglossoideae with maximum support. The remaining Cynoglossoideae consists of four tribes: Rochelieae, which is successively sister to Craniospermeae (LPP = 1, UFBoot = 100), with Craniospermeae successively sister to a clade comprising Myosotideae and Cynoglosseae (LPP = 0.93, UFBoot = 100). The subtribes Heterocaryinae and Eritrichiinae of the tribe Rochelieae are also retrieved as sister taxa with maximum support. There are some ambiguities in the large tribe Cynoglosseae. Even though Bothriosperminae + Cynoglossinae receives maximum support and is retrieved as sister to Microulinae + Amsinckiinae with maximum support, Microulinae + Amsinckiinae receives only moderate support in the MuLo-Exn species tree (LPP = 0.80) but maximum support in the ConcaExn tree. Moderate to rarely low resolution for the MuLo-Exn species tree and significant tree discordance is recovered regarding some of the intergeneric relationships within the tribe Lithospermeae and within the subtribes Boragininae, Cynoglossinae and Amsinckiinae. Most of these ambiguities are fully resolved in the concatenation analysis, but this occasionally coincides with differences in topology (see Fig. 2 and Fig. S4). Noteworthy is that within Cynoglossinae, *Microparacaryum intermedium* (Fresen.) Hilger & Podlech is sister to the remaining species (*Cynoglossum s.l*.) with maximum support in the concatenation analysis, but is poorly supported as sister to a clade including *Brandella* R.R.Mill and *Lindelofia* Lehm. in the MuLo-Exn species tree (LPP = 0.55).

Boraginales II also shows well-supported and well-resolved relationships in the MuLo-Exn species tree (Fig. 3). The basally branching clade consists of Namaceae and Hydrophyllaceae. The two families together form a fully supported monophylum, with Namaceae paraphyletic and Hydrophyllaceae nested in Namaceae. The *Nama* clade is sister to Hydrophyllaceae plus the remainder of Namaceae (LPP = 0.86, UFBoot = 100). *Nama* itself is retrieved as polyphyletic due to the placement of *Nama rothrockii* A.Gray as sister to *Eriodictyon* Benth. *Wigandia* Kunth is sister with maximum support to the clade comprising *Nama rothrockii* and *Eriodictyon* Benth. In Hydrophyllaceae, all relationships are fully resolved and supported. *Draperia* Torr. is sister to the Romanzoffieae + Hydrophylleae clade. Romanzoffieae comprises *Romanzoffia* Cham. as sister to *Phacelia* Juss. Hydrophylleae include *Emmenanthe* Benth., *Eucrypta* Nutt., *Ellisia* L., *Hydrophyllum* L., *Nemophila* Nutt. ex W.P.C.Barton and *Pholistoma* Lilja as successive sister groups.

The basal clade of Boraginales II (Namaceae and Hydrophyllaceae) is sister to the remaining families of the clade with maximum support. Ehretiaceae comprise two major sister clades (LPP = 1, UFBoot = 100). Ehretiaceae I includes the *Ehretia* P.Browne clade as sister to *Halgania* Gaudich. + *Keraunea* Cheek & Sim.-Bianch. with maximum support. *Rotula aquatica* Lour. is retrieved in *Ehretia*. Ehretiaceae II is not as well resolved, but Lennoaceae is fully supported as nested in this clade. *Tiquilia* Pers. is sister to Lennoaceae with low support (LPP = 0.27, UFBoot = 97) and together they are sister to a clade comprising *Bourreria* P.Browne + (*Lepidocordia* Ducke + *Rochefortia* Sw.) with high support (LPP = 1, UFBoot = 97). *Bourreria* is sister to *Lepidocordia* + *Rochefortia* with moderate support (LPP = 0.85, UFBoot = 97), but the sister relationship of *Lepidocordia* and *Rochefortia* is fully supported in both analyses.

Ehretiaceae are sister to a clade including Hoplestigmateceae, Coldeniaceae, Cordiaceae and Heliotropiaceae, with monogeneric Hoplestigmataceae sister to the monotypic Coldeniaceae + Cordiaceae. Within Cordiaceae, neither *Cordia* L. nor *Varronia* P.Browne are monophyletic. Members of the *Sebestena*-group according to Gottschling *et al.* (2005), namely *Cordia sebestena* L., *Cordia sonorae* Rose and *Cordia decandra* Hook. & Arn., are basally branching lineages of Cordiaceae, but do not group together. *Varronia salviifolia* (Juss. ex Poir.) Borhidi is retrieved in this basal grade as sister to *Cordia sebestena.* The representatives of the *Myxa* group are retrieved as sister to the remaining species of *Varronia*. The clade comprising Hoplestigmataceae, Coldeniaceae and Cordiaceae is retrieved as sister to Heliotropiaceae with high to moderate support in the analyses based on only exons (MuLo-Exn LPP = 0.95, ConcaExn UFBoot = 73). The crown node of Heliotropiaceae is fully supported in both trees; however, there are topological incongruences. In the MuLo-Exn species tree, *Ixorhea* is retrieved as sister to the *Heliotropium* clade (LPP = 0.76), while in the ConcaExn species tree it is retrieved as sister to *Euploca* Nutt. + *Myriopus* Small (UFBoot = 99). Phylogenetic relationships within *Heliotropium* Tourn. ex. L. are fully resolved. Specifically, South American *H.* sect. *Heliothamnus* is successively sister to the Old World *Heliotropium* clade [including *Nogalia drepanophylla* (Baker) Verdc.], which is sister to *H.* sect. *Cochranea* + *H.* sect. *Tournefortia*.

The phylogenetic trees of the supercontig dataset (MuLo-SupC; Fig. 4, Supplementary Data Fig. S3 and ConcaSupC; Fig. S5) are largely congruent with the species trees of the exon dataset (MuLo-Exn and ConcaExn), but with some major topological differences: Codonaceae are retrieved with maximum support as sister to Boraginales II instead of Boraginales I in both MuLo-SupC and ConcaSupC species trees. In Boraginales I, there are some noteworthy differences in Boraginaceae. Subfamily Echiochiloideae is retrieved as sister to Boraginoideae and together they are sister to Cynoglossoideae in both MuLo-SupC and ConcaSupC species trees. Within Cynoglossoideae, Trichodesmeae and Lasiocaryeae are retrieved as sister tribes with high support (LPP = 0.95), but only for the MuLo-SupC species tree. These two tribes are retrieved as successive sister taxa in all other trees (ConcaSupC, MuLo-Exn and ConcaExn). Similarly, a sister relationship of the tribes Omphalodeae and Asperugeae is fully supported in both MuLo-SupC and ConcaSupC species trees. Myosotideae is successively sister to Craniospermeae and not the other way around, but only in the MuLo-SupC species tree. Support for Microulinae* + *Amsinckiinae is increased (LPP = 0.95 in MuLo-SupC, UFBoot = 100 in ConcaSupC, but LPP = 0.8 in MuLo-Exn). In Cynoglossinae, *Microparacaryum intermedium* is retrieved as sister to the remaining taxa of the subtribe in both MuLo-SupC and ConcaSupC (LPP = 0.44, UFBoot = 100), a topology that was recovered in the ConcaExn species tree as well.

In Boraginales II, the sister relationship of the *Nama* clade to the remaining Namaceae + Hydrophyllaceae is fully supported in all trees (MuLo-Exn, MuLo-SupC, ConcaExn, ConcaSupC). Furthermore, there some topological incongruences regarding Ehretiaceae and Heliotropiaceae. In the MuLo-SupC species tree, within Ehretiaceae, *Tiquilia* is sister to the remaining taxa (LPP = 1) while Lennoaceae is successively sister to *Bourreria* (LPP = 0.71), and *Bourreria* sister to *Lepidocordia* + *Rochefortia* with increased support (LPP = 0.99) as in the MuLo-Exn species tree. The ConcaSupC is the only tree that yields a fully resolved Ehretiaceae, but *Tiquilia* is retrieved as successively sister to Lennoaceae which is the successive sister group to the *Ehretia* + *Halgania* and the *Bourreria* + *Lepidocordia + Rochefortia* clades. Within Heliotropiaceae, *Ixorhea* is fully supported as sister to *Heliotropium* in the MuLo-SupC species tree, but is fully supported as sister to the *Myriopus* *+ Euploca* clade in the ConcaSupC species tree. A direct comparison between the MuLo-SupC and the MuLo-Exn species trees can be found in Fig. 4.

### Gynoecial and fruit characters

**Fig. 5.**
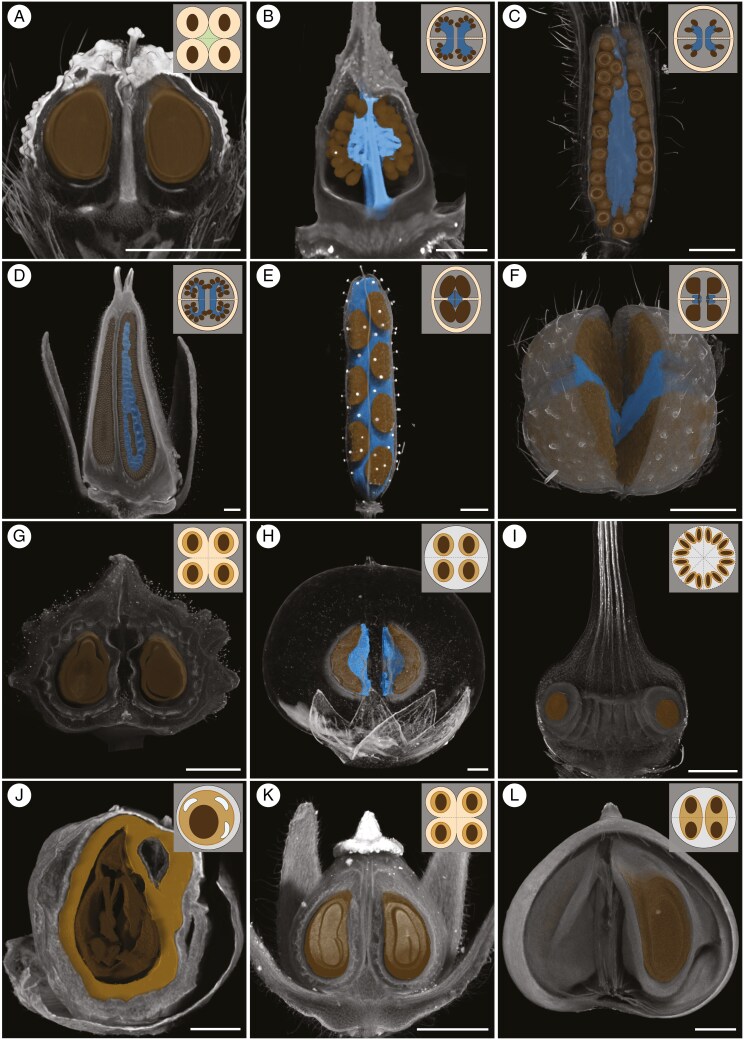
Micro-computed tomography (μCT)-based volume rendering of fruits (A, C, E–L) or gynoecia (B, D) of selected Boraginales showing segmentations of seeds (brown) and placentae (blue). If not stated otherwise, the volume renderings are virtually cut and the lateral view (looking at the line of carpel fusion) is displayed. The schematic drawings at the upper right corner show a median cross-section of the fruit/gynoecium. (A) *Ogastemma pusillum* (Coss. & Durieu ex Bonnet & Barratte) Brummitt; view of two mericarpids; gynobase present. (B) *Codon schenckii* Schinz; anthetic gynoecium. (C) *Nama jamaicensis* L.; closed capsule. (D) *Wigandia ecuadorensis* Cornejo; post-anthetic gynoecium. (E) *Emmenanthe penduliflora* Benth.; closed capsule; the pericarp is reconstructed and displayed with low opacity and solidity. (F) *Phacelia malvifolia* Cham. & Schltdl.; top-view of an opened capsule; the fruit is not virtually cut. (G) *Coldenia procumbens* L.; view of two mericarpids. (H) *Bourreria succulenta* Jacq.; drupe; the exocarp, fleshy mesocarp and lignified endocarp are reconstructed and displayed with low opacity and solidity; seeds of two endomericarpids are displayed. (I) *Pholisma arenarium* Nutt.; closed dehiscent fruit with endomericarpids; consists of 11 carpels. (J) *Varronia cylindristachya* Ruiz & Pav.; one-seeded drupe with undivided endocarp; the lignified endocarp is segmented and shown in light brown. (K) *Heliotropium arbainense* Fresen.; view of two mericarpids. (L) *Tournefortia undulata* Ruiz & Pav.; view of a synendomericarpid. Scale bars = 1 mm.

**Fig. 6.**
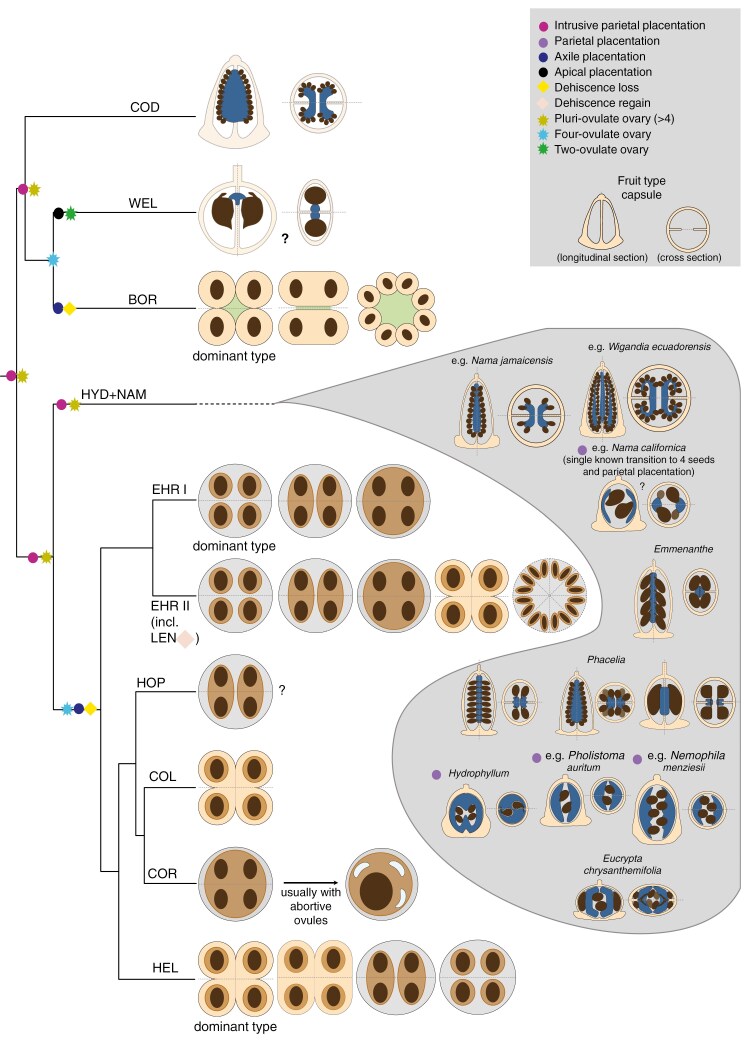
Hypothetical evolutionary series of fruit diversification in Boraginales I (Codonaceae, Wellstediaceae, Boraginaceae) and in Boraginales II (Namaceae, Hydrophyllaceae, Ehretiaceae, Hoplestigmataceae, Coldeniaceae, Cordiaceae, Heliotropiaceae). Cladogram is based on the MuLo-Exn species tree. Morphological characters are defined based on the morphological assessment made in this study. The schematic representation of gynoecia/fruits follows the terminology presented in Box 1. Capsules are loculicidal except for Wellstediaceae (septicidal). The apomorphic dehiscent fruit of Lennoaceae opens via an irregular circumscissile rupturing. Question marks indicate uncertainty due to lack of available morphological data. Within the basal grade of Boraginales II (NAM + HYD) internal ovary architectures remain largely unexplored and only the known types are displayed. The direction of evolution of different internal ovary architectures within each clade is not displayed due to its complexity and uncertainty.

Gynoecium and fruit characters were retrieved for 90 taxa from the literature, supplemented with novel SEM and μCT data for 80 and 91 taxa respectively. Detailed studies of the internal ovary/fruit architecture are presented in in Fig. 5, showing almost all known types of fruit and seed and pericarp integration as listed in Box 1. These data are summarized in a schematic analysis of fruit types across Boraginales (Fig. 6).

## DISCUSSION

### Efficacy of Angiosperms353 and methodological considerations in Boraginales

This is the first study using the Angiosperms353 probe set across Boraginales and to have a comprehensive sampling covering almost 80 % of its genus diversity. Only four previous studies adopted a phylogenomic approach in this group; however, Zhang *et al.*, (2020b) covered the entire asterid clade, encompassing about 17 orders and ca. 110 families, and therefore had limited sampling within Boraginales. Cohen (2022) focused solely on the genus *Lithospermum* L. and Meudt *et al*. (2025) focused solely on the genus *Myosotis* L., while Cohen (2025) used both lineage-specific and Angiosperms353 loci, focusing on the phylogenetic relationships within Boraginaceae. Our results demonstrate the effectiveness of the Angiosperms353 probe set at all taxonomic levels, which is in line with several recent studies across asterids, utilizing the Angiosperms353 probe set in particular, or other target enrichment approaches in general (Bagley *et al.*, 2020; Antonelli *et al.*, 2021; Thomas *et al.*, 2021*b*; Ottenlips *et al.*, 2021). The overall phylogenetic relationships recovered here largely confirm the topologies from previous studies (Ferguson, 1998; Gottschling *et al.*, 2001, 2005; Moore and Jansen, 2006; Thomas *et al.*, 2008; Luebert *et al.*, 2011a; Hasenstab-Lehman and Simpson, 2012; Weigend *et al.*, 2013, 2014; Gottschling *et al.*, 2014a; Chacón *et al.*, 2016; Hasenstab-Lehman, 2017; Vasile *et al.*, 2020; Zhang *et al.*, 2020b; Cohen, 2022, 2025). However, the extensive sampling combined with the phylogenomic approach resolves several long-standing phylogenetic questions discussed below, especially regarding the family-level relationships of Namaceae/Hydrophyllaceae and Lennoaceae, and the relationships within Cynoglossoideae.

Nevertheless, genomic data as used in this study are known to be prone to gene tree discordance and gene paralogy (Smith *et al.*, 2015, 2020; Ufimov *et al.*, 2022), which can impact the accuracy of phylogenetic reconstruction. We explored various datasets (exons, exons with multi-copy genes and supercontigs) and methods (coalescence, ASTRAL-Pro3 and concatenation) to address potential biases. Supercontigs have been shown to generate more robust phylogenies with higher support values along internal nodes (Murphy *et al.*, 2020; Bagley *et al.*, 2020; Thomas *et al.*, 2021*a*) but in our case topologies of exons and supercontigs were equally well supported (Fig. 4; Supplementary Data Figs S4 and S5). Concatenation and coalescent approaches often generate similar topologies for datasets with overall strong phylogenetic signal (as for example in Gentianales: Antonelli *et al.*, 2021; Myrtales: Maurin *et al.*, 2021; Sapindaceae: Buerki *et al.*, 2021; Apiaceae: Clarkson *et al.*, 2021; and Ochnaceae: Shah *et al.*, 2021) and that was no different for Boraginales, yet with a few topological differences and often higher support values in the ConcaExn and ConcaSupC species trees.

### Systematic relationships within Boraginales I

The Boraginales I clade has been considered as including three highly distinctive families: Codonaceae sister to Wellstediaceae + Boraginaceae (Ferguson, 1998; Nazaire and Hufford, 2012; Refulio‐Rodriguez and Olmstead, 2014; Weigend *et al.*, 2014). Our exonic dataset confirms this relationship; however, the longer and more variable dataset of supercontigs retrieved Codonaceae as sister to the remaining Boraginales II (Fig. 4). Codonaceae are unique within Boraginales I. Their polymerous flowers, pluri-seeded capsular fruits, endospermous seeds and unique pluriseriate, mineralized spines clearly set them apart from Wellstediaceae [1(2)-seeded capsular fruits and tetramerous flowers; Hilger and Weigend, 2016] and from Boraginaceae (4 nutlets, pentamerous flowers and exendospermous seeds; Weigend *et al.*, 2016). Historically, *Codon* was considered as closely related to Hydrophyllaceae in the traditional sense (Brand, 1913), due to similar pollen, gynoecium, fruit and seed morphology. After molecular data indicated that *Codon* is closely allied to Boraginales I, these shared character states with Hydrophyllaceae were considered as plesiomorphic (Weigend and Hilger, 2010). Conversely, a sister relationship of Codonaceae to Hydrophyllaceae and Namaceae is supported by a set of (floral) morphological characters, and therefore the topological contrast of different datasets in our study requires more attention. The question of the exact placement of Codonaceae remains inconclusive at this stage, but it is consistently retrieved as an independent lineage.

Our phylogeny provides the best resolution and highest support so far for the infrafamilial relationships of Boraginaceae and is largely congruent with previous molecular studies (Weigend *et al.*, 2009, 2010, 2013; Chacón *et al.*, 2016, 2019). Previously unresolved or ambiguous relationships in the backbone are here resolved. According to the MuLo-Exn species tree: Trichodesmeae is resolved as sister to the remaining Cynoglossoideae, while Chacón *et al.* (2016) had them as sister to Lasiocaryeae and these together as sister to the remaining Cynoglossoideae. There is maximum support for a successive sister relationship of Omphalodeae to Asperugeae (unresolved in Chacón *et al.*, 2016). Asperugeae are found as successively sister to Rochelieae with moderate support. Craniospermeae are successively sister to Myosotideae with maximum support and then to Cynoglossae with high support, while previously they were retrieved as sister to each other with low support. Finally, Microulinae is moderately supported as sister to Amsinckiinae.

Interestingly, the main topological differences between the exon and supercontig datasets, as well as between the multi-locus species trees and concatenation analyses are broadly consistent with the results of Cohen (2025), where conflicting phylogenetic signal between different analyses are also correlated with greater gene conflict and moderate to weak quartet concordance. Previous nuclear datasets (Nazaire and Hufford, 2012) retrieved Echiochiloideae as sister to Boraginoideae and not to Boraginoideae + Cynoglossoideae, a relationship that we also retrieved when we used the supercontig dataset. There is maximum support for a sister relationship of Asperugeae + Omphalodeae in all analyses except for the MuLo-Exn tree, where they are retrieved as successive sister clades. Trichodesmeae are retrieved as sister to Lasiocaryeae – as in Chacón *et al.* (2016) – only in the MuLo-SupC tree. The successive sister relationship of Craniospermeae to Myosotideae in the exonic dataset is reversed only in the MuLo-SupC tree, where Myosotideae is successively sister to Craniospermeae. Most incongruences are found between the two multi-locus species trees (Mulo-Exn and Mulo-SupC), indicating complex evolutionary gene histories causing tree discordance (see Figs 2–4 and the ASTRAL-Pro3 species tree in Supplementary Data Fig. S6).

### Systematic relationships within Boraginales II

The Boraginales II clade is a natural group including Namaceae, Hydrophyllaceae, Ehretiaceae, Lennoaceae, Coldeniaceae, Hoplestigmataceae, Cordiaceae and Heliotropiaceae (Luebert *et al.*, 2016), which is congruent with our results and previous studies (Weigend *et al.*, 2014; Zhang *et al.*, 2020b). Nevertheless, there are some differences in this study. The exact relationships of the basal grade of Boraginales II (Hydrophyllaceae and Namaceae) were ambiguous (Ferguson, 1998; Nazaire and Hufford, 2012; Refulio‐Rodriguez and Olmstead, 2014; Weigend *et al.*, 2014) until recently, when Zhang *et al.*, (2020b) retrieved them as sister to each other. That result, however, was based on only four species of Hydrophyllaceae and two of Namaceae, with the largest genus *Nama* (53 of ca. 75 spp. in Namaceae) not represented. In the present study, several *Nama* species are retrieved as a basally branching clade sister to Namaceae + Hydrophyllaceae in all analyses, but with a very short branch length and not well supported by quartet concordance. Cohen (2025) retrieved the *Nama* clade as sister to Hydrophyllaceae with moderate support, but with *Nama dichotoma* (Ruiz & Pav.) Choisy as the only representative of the genus. We confirm the previously shown polyphyly of *Nama* (Ferguson, 1998; Vasile *et al.*, 2020) and the need for a new name for *N. rothrockii* (Guilliams and Hasenstab-Lehman, 2024). *Turricula parryi* and *Nama lobbii* A.Gray have been included in *Eriodictyon* in Jepson eFlora (Hannon, 2021), but a formal taxonomic revision is still pending. Most importantly, family delimitation should be adjusted and Namaceae should be re-included in Hydrophyllaceae, in line with most classical studies (Brand, 1913; Heckard, 1963; Bacon, 1987; Di Fulvio *et al.*, 1997; Hofmann *et al.*, 2016).

Not all intergeneric relationships in Ehretiaceae are fully resolved (Figs 3 and 4), but we confirm the inclusion of Lennoaceae in Ehretiaceae as suggested by previous studies (Nazaire and Hufford, 2012; Weigend *et al.*, 2014; Gottschling *et al.*, 2014a; Zhang *et al.*, 2020b) and by recent and detailed morphological data (Jeiter *et al.*, 2023). Lennoaceae have been traditionally treated as a separate family due to their parasitic lifestyle (Bittrich, 2016) and their unique morphological traits (Yatskievych and Mason, 1986; Jeiter *et al.*, 2023). Most phylogenetic studies in Boraginales addressing the position of Lennoaceae suggest long branch attraction (Nazaire and Hufford, 2012; Gottschling *et al.*, 2014*a*; Weigend *et al.*, 2014; Hasenstab-Lehman, 2017), but our data do not. Furthermore, almost all previous phylogenetic studies were based essentially on chloroplast data, which is problematic due the reduced plastome in parasitic Lennoaceae (Schneider *et al.*, 2018). Long branch attraction on chloroplast phylogenies is rather common for parasitic (Su *et al.*, 2015) and mycoheterotrophic lineages (Feng *et al.*, 2016). The peculiar Brazilian *Keraunea* has been recently recognized as a member of Boraginales and this is here clearly confirmed, as well as its sister relationship to Australian *Halgania* (Almeida *et al.*, 2023; Moonlight and Cardoso, 2023). Generic limits in Ehretiaceae are largely confirmed, but with limited sampling in *Bourreria* (3 of 52 spp.) and *Ehretia* (4 of 66 spp.). *Rotula aquatica* is found nested in *Ehretia*, confirming the findings of Gottschling and Hilger (2004*b*).

Cordiaceae historically included only the large (ca. 340 spp.) and broadly defined genus *Cordia*, with five to eight sections or subgenera (Johnston, 1930). Borhidi *et al.* (1988) re-evaluated morphological data and proposed the separation of three genera: *Cordia*, *Varronia* and *Gerascanthus* P.Brown, but only the genus *Varronia* was formally reinstated based on molecular evidence (Gottschling *et al.*, 2005; Gottschling and Miller, 2007; Weeks *et al.*, 2010). Our taxon sampling only provides a limited insight into the large genera *Cordia s.l*. (>200 spp.) and *Varronia* (>140 spp.), but our data strongly indicate that genus limits need to be adjusted with neither of the two genera resolved as monophyletic.

Heliotropiaceae taxonomy has been in flux for decades (Frohlich *et al.*, 2022). The four genera currently recognized ((*Heliotropium* (*Ixorhea* (*Euploca*, *Myriopus*))); Luebert *et al.*, 2016) are confirmed as monophyletic in the present study. Cohen (2025) mentions that *Heliotropium* is not monophyletic, but this is only due to the older nomenclature used. A novel sister relationship of *Ixorhea* to *Heliotropium* is supported by the multi-locus species trees (MuLo-Exn, MuLo-SupC, see Figs 3 and 4 and note the high percentage of tree discordance), but the rest of the trees (ConcaExn, ConcaSupC and ASTRAL-Pro3) support *Ixorhea* as sister to *Euploca* and *Myriopus*, as found by previous studies based on chloroplast data (Luebert *et al.*, 2011*b*; Weigend *et al.*, 2014). The *Heliotropium* clade comprises four major clades: *Heliotropium* sect. *Heliothamnus* I.M.Johnst., Old World *Heliotropium*, *Heliotropium* sect. *Cochranea* (Miers) Post & Kuntze, and the *Tournefortia*-clade, the last comprising *Tournefortia* sect. *Tournefortia* and all remaining New World species of *Heliotropium* (Hilger and Diane, 2003; Luebert and Wen, 2008; Luebert *et al.*, 2011*b*). All four clades are retrieved as monophyletic with maximum support and their relationships in the same order as in previous studies.

### Fruit diversity of Boraginales

Highly resolved and supported phylogenies provide an excellent basis for understanding the evolution of morphological characters, especially when associated with ontogenetic data (Jeiter and Smets, 2023, 2024). Over the last few decades ontogenies with high coverage of developmental stages for a majority of Boraginales families (*sensu*  Luebert *et al.*, 2016) have been generated. The ontogenies focus on, but are not limited to, gynoecium and fruit development; they have allowed detection of structural innovations (e.g. Diane *et al.,* 2002; Jeiter *et al.*, 2018) and identification of the processes involved in changes in ovule number, placentation, or seed and pericarp integration (Gottschling *et al.*, 2014*b*; Weigend *et al.*, 2014; Vasile *et al.*, 2021, 2022; Jeiter *et al.*, 2023).

The development of capsular fruits in the basal clades is very similar and there is strong evidence for pluri-seeded capsule as the ancestral state of the order (Vasile *et al.*, 2021). Within Boraginales I, Codonaceae have pluri-ovulate ovaries with intrusive parietal placentation (Jeiter *et al.*, 2016), but ovule number is probably reduced to four at the crown node of Wellstediaceae and Boraginaceae (Fig. 6). This reduction is accompanied by transitions in placentation (apical in Wellstediaceae; axile in Boraginaceae) and a loss of dehiscence and formation of eremocarps (all Boraginaceae). Ontogenetic data for Boraginaceae (Hilger, 2014; Luebert *et al.*, 2016 and unpublished data) show that eremocarps form through localized carpel bulging, creating a gynobase with a persistent style sitting atop a structure called columella. Eremocarps are dispersed while style and gynobase remain attached to the plant. Seeds are firmly embedded in the pericarp in Boraginaceae (Fig. 5A), but details of gynobase and eremocarp morphology and sculpturing are extraordinarily diverse and intricately linked to an extremely diversified dispersal biology (Cynoglossoideae: Selvi *et al.*, 2011; Otero *et al.*, 2019*a*, *b*).

The basal grade of Boraginales II has been studied extensively (Di Fulvio, 1991, 1998; Di Fulvio *et al.*, 1993, 1997; Hofmann, 1999; Berg, 2009; Vasile *et al.*, 2021, 2022). Parietal or intrusive placentation permits dramatic shifts in ovule number (four to ca. 200 ovules in Namaceae and Hydrophyllaceae; Figs 5C–F and 6). Shifts of ovule/seed number are readily reversible as long as the fruits remain dehiscent, and paedomorphosis is probably a crucial process leading to anatomical changes and the convergent reduction to four ovules (Vasile *et al.*, 2021, 2022). As in Boraginaceae, the remaining lineages of Boraginales II (Ehretiaceae, Hoplestigmataceae, Coldeniaceae, Cordiaceae, Heliotropiaceae) are characterized by a fixation to four ovules/seeds accompanied with loss of dehiscence, a transition to axile placentation (Fig. 6) and an integration of the seeds into the pericarp. An evolutionary novelty within Boraginales II (excl. Hydrophyllaceae and Namaceae) is that the seeds are embedded in a hard, multi-layered endocarp, providing a considerable advantage for plants that often live in habitats that are only intermittently supplied with water (see, for example, Hilger, 1989; Diane *et al.*, 2002; Gottschling *et al.*, 2014*b*). Another apomorphy is observed in Lennoaceae which have an increase in seed number via carpel multiplication and release the multiple endomericarpids via an irregular rupturing of the meso- and exocarp following the desiccation of these tissues (Jeiter *et al.*, 2023)

Heterochrony and heterotopy have been crucial for modifications of individual structures and lead to divergent and clade-specific ovary architectures via different process combinations and synorganization of these structures (Fig. 6). Minor heterochronous shifts in individual aspects of the developmental trajectories – placentation, ovule initiation, ovary expansion – can lead to very different mature forms (Vasile *et al.*, 2021, 2022). Heterotopy is also common with, for example, relocation of placentae to an apical or subapical position through elongation of the basal septa [e.g. in *Tiquilia* Pers.: Gottschling *et al.*, 2014*b* and in *Draperia systyla* (A.Gray) Torr.: Vasile *et al*., 2021], but also changes in ovule position, orientation and shape. The bifacial placentae of *Eucrypta chrysanthemifolia* (Benth.) Greene appear to be an extreme case of heterotopy combined with heterochrony. Heterotopy was identified also in Ehretiaceae and Lennoaceae (position of ovarian cavity, compitum and transmitting tissues; Jeiter *et al.*, 2023).

Evidently, most of the anatomical and internal fruit diversity of Boraginales is concentrated in Boraginales II, and the topic has been in the centre of a range of studies in recent decades (Berg, 1985; Bacon, 1987; Hilger, 1987, 1992, 2014; Di Fulvio *et al.*, 1997, 1999; Gottschling, 2004; Gottschling and Hilger, 2004*b*; Gottschling*et al.*, 2014*b*; Holstein and Gottschling, 2018; Jeiter *et al.*, 2018, 2023; Heigl *et al.*, 2020; Vasile *et al.*, 2021, 2022). Septation and placentation have repeatedly been proposed as highly variable features crucial for the evolution of divergent fruit types. In particular, transitions to axile placentation represent a key factor for fruit diversification. Shivaprakash and Bawa (2022) suggest a derived evolution of parietal placentation from axile placentation by retraction of the septa, as reported by others (Lindsey, 1940; Wilkinson, 1944). However, they do not reject the opposite scenario, such as intruding parietal placentae turning into axile placentation. This phenomenon is evident in Boraginales I and II. Axile placentation, which is dominant in angiosperms, isolates the ovules in well-separated locules, thereby reducing competition of developing seeds for finite resources (Shivaprakash and Bawa, 2022). The assignment of the ovules to separate locules appears to open the way to various levels of seed and pericarp integration and the formation of a range of different dispersal units (endomericarpid, endomericarp, synendomericarpid, mericarpid, mericarp, synmericarpid; Box 1). Furthermore, Shivaprakash and Bawa (2022) demonstrate that the evolution of placentation is associated with an increase or decrease of ovule number. The morphological assessment performed in the present study further underlines these conclusions and suggests a whole range of mechanisms for fruit diversification based on modifications of internal ovary/fruit architectures (Figs 5 and 6).

## CONCLUSION

Our data show that the Angiosperms353 probe set can be successfully employed for phylogenetic reconstruction across many different taxonomic levels in Boraginales. We were able to fill most of the crucial sampling gaps at the family and genus levels, such as *Hoplestigma* Pierre from Hoplestigmataceae, *Rochefortia* and *Keraunea* from Ehretiaceae, and within Boraginaceae at tribal (e.g. Omphalodeae) and subtribal (e.g. Microulinae) levels compared to previous studies. This, together with the improved resolution and support, permits us to balance genus representation and species diversity across the order and to resolve relationships, including many long-standing questions, at both the family and subfamily levels. Our analyses do retrieve a limited degree of topological conflict, possibly reflecting divergent evolutionary histories of different genes. Topological incongruences between different datasets (exons and supercontigs) and between different methods (coalescent-based species tree and concatenation) should receive additional attention in the future (e.g. in Ehretiaceae). Indisputably, the family-level classification can be further resolved compared to Luebert *et al.* (2016), with the recognition of a total of nine families. In Boraginales I we propose the recognition of the three families Codonaceae, Wellstediaceae and Boraginaceae, independent of the possible placement of Codonaceae at the base of Boraginales II. In Boraginales II we propose the recognition of the six families Hydrophyllaceae (including Namaceae), Ehretiaceae (including Lennoaceae), Hoplestigmataceae, Coldeniaceae, Cordiaceae and Heliotropiaceae. The data presented here cast doubt on the monophyly of genera in Cordiaceae (*Cordia* vs. *Varronia*). Taxonomy in species-rich, widespread (sub-)tropical, woody Cordiaceae and Ehretiaceae is poorly resolved and both an in-depth revisionary effort and comprehensively expanded sampling would be required to satisfactorily address details of their phylogeny and classification. In general, taxonomic and classificatory problems still abound mostly in the tropical, woody lineages such as *Tournefortia*, *Cordia*, *Varronia*, *Ehretia* and *Bourreria*, but also some temperate lineages such as Cynoglosseae, and can be possibly resolved by the development of a suitable custom probe set.

The phylogenies provided here together with the comprehensive ontogenetic assessment of comparative morphology across the order aimed to identify potential key characters for the evolution of the order and we suggest the complex internal ovary architectures as such. Clade-specific evolutionary pathways and how these might be related to diversification rates, especially in groups with high diversity in fruit anatomy (e.g. Heliotropiaceae, Ehretiaceae), remain to be addressed in the future. Furthermore, across Boraginales, modifications of the corolla, especially the stamen–corolla tube, but also the different style morphologies and the integration of style and stigma (in Heliotropiaceae) are of crucial importance for the evolutionary trajectories and will need to be explored in more detail. Similarly, morphological and ontogenetic studies including poorly understood woody tropical lineages such as Hoplestigmataceae would be highly desirable to close remaining gaps in our understanding of evolutionary trajectories.

## SUPPLEMENTARY DATA

Supplementary data are available at *Annals of Botany* online and consist of the following.

Table S1: Species included in this study and voucher information. Table S2: Gene recovery statistics produced with the hybpiper_stats.py script available with HybPiper. Table S3. Scanner and reconstruction settings. Figure S1: Heatmap visualizing percentage length recovery for each gene, relative to the mean of the target-file references, produced with the gene_recovery_heatmap.py script available with HybPiper. Figure S2: Heatmap visualizing the paralogue distribution according to the paralogue report table, produced with the paralog_retriever.py script available with HybPiper. Figure S3: Phylogenetic relationships among the Boraginales. ASTRAL-III species tree generated using the retrieved supercontigs of 349 nuclear loci from the Angiosperms353 probe set (MuLo-SupC). Numerical values are local posterior probabilities (LPP). Figure S4: Phylogenetic relationships among the Boraginales based on the concatenation analysis with IQTREE using the retrieved exonic regions of 349 nuclear loci from the Angiosperms353 probe set (ConcaExn). Numerical values are ultrafast bootstrap approximation (UFBoot). Figure S5: Phylogenetic relationships among the Boraginales based on the concatenation analysis with IQTREE using the retrieved supercontigs of 349 nuclear loci from the Angiosperms353 probe set (ConcaSupC). Numerical values are ultrafast bootstrap approximation (UFBoot). Figure S6: Phylogenetic relationships among the Boraginales. ASTRAL-Pro3 species tree generated using all the retrieved copies of the exonic regions of 349 nuclear loci from the Angiosperms353probe set, accounting for orthology and paralogy. Numerical values are local posterior probabilities (LPP).

mcaf061_suppl_Supplementary_Figures_S1

mcaf061_suppl_Supplementary_Figures_S2

mcaf061_suppl_Supplementary_Figures_S3

mcaf061_suppl_Supplementary_Figures_S4

mcaf061_suppl_Supplementary_Figures_S5

mcaf061_suppl_Supplementary_Figures_S6

mcaf061_suppl_Supplementary_Tables_S1

mcaf061_suppl_Supplementary_Tables_S2

mcaf061_suppl_Supplementary_Tables_S3

## FUNDING

D.Cardoso thanks Conselho Nacional de Desenvolvimento Científico e Tecnológico (Research Productivity Fellowship, grant no. 314187/2021-9) and Fundação Carlos Chagas Filho de Amparo à Pesquisa do Estado do Rio de Janeiro (JCNE grant no. E-26/200.153/2023) for supporting his research on plant phylogenomics and evolution. We want to express our gratitude for the funding of the new SEM-Unit based on grant INST 217/1078-1 FUGG of the Deutsche Forschungsgemeinschaft. Funding for the Skyscan 1272 of BIOB, Animal Diversity Section was provided by the Deutsche Forschungsgemeinschaft, INST 217/849-1 FUGG.
