# Supplementary figures and images for: An updated phylogeny of Boraginales based on the Angiosperms353 probe set: a roadmap for understanding morphological evolution
Source: Ann Bot. 2025 Apr 10;136(1):77–97. doi: 10.1093/aob/mcaf061 (PMC12401892; doi:10.1093/aob/mcaf061)

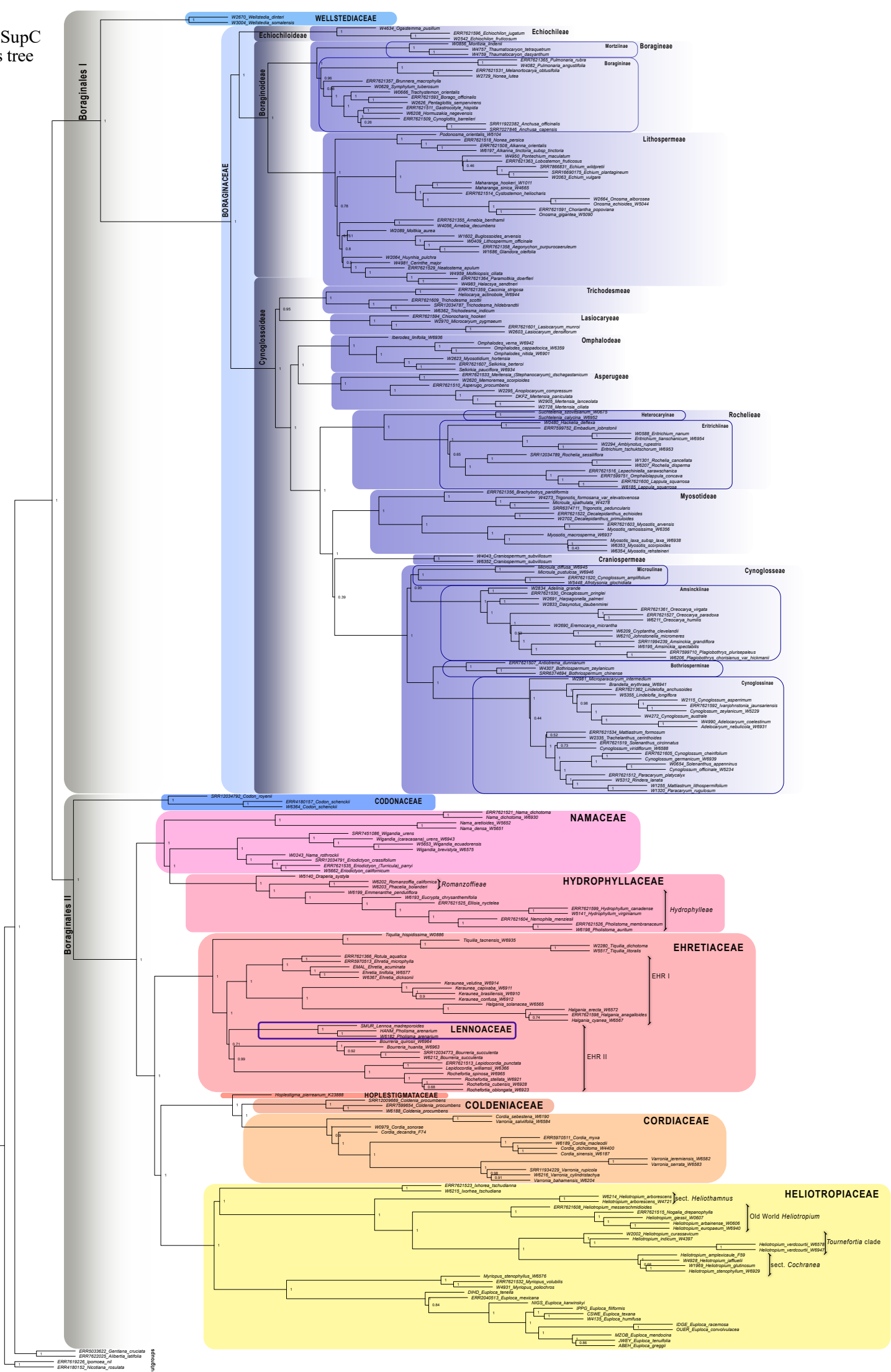

Supplement: mcaf061_suppl_Supplementary_Figures_S3 [file mcaf061_suppl_supplementary_figures_s3.pdf]

ConcaExn Species tree

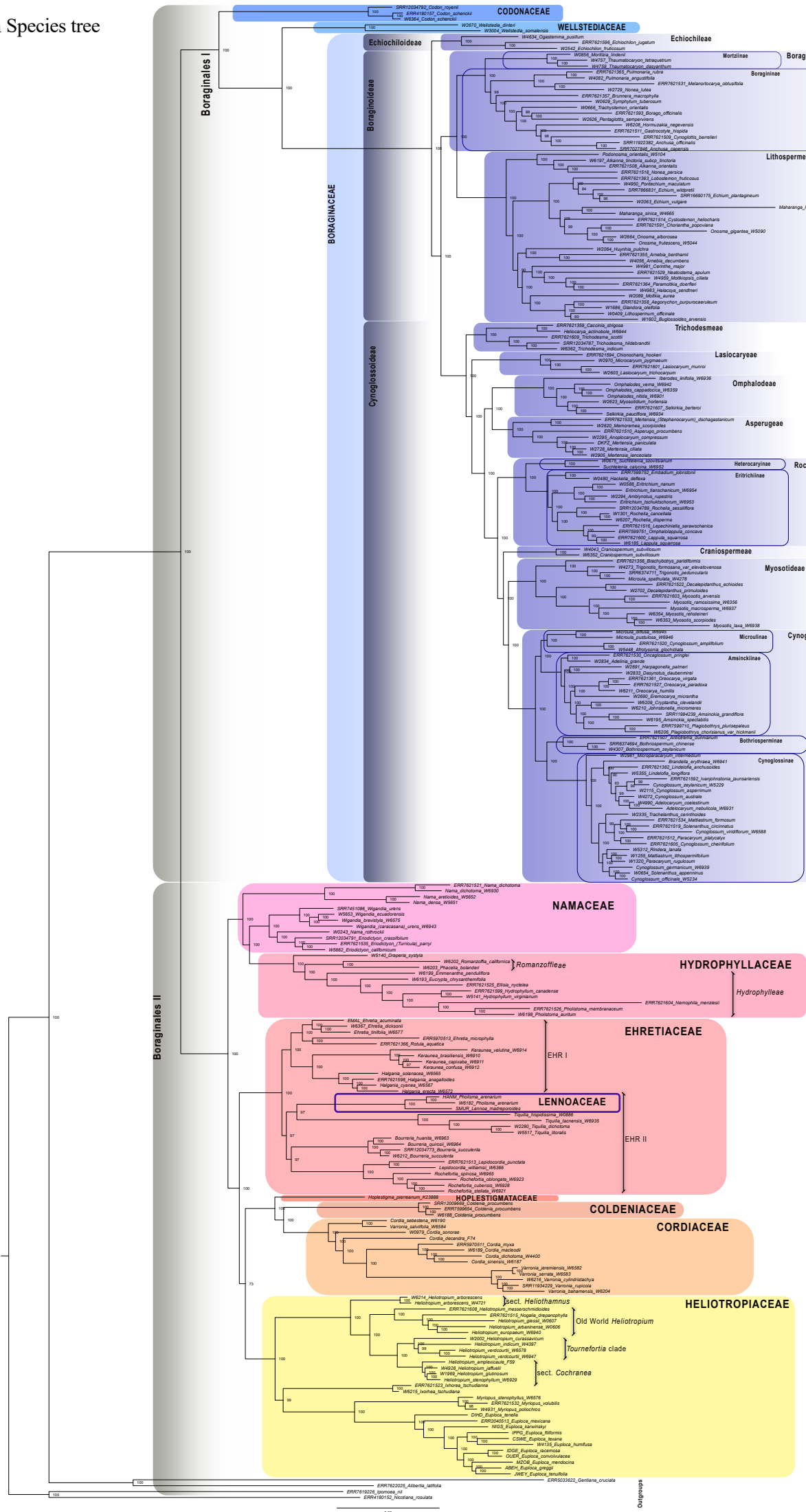

Supplement: mcaf061_suppl_Supplementary_Figures_S4 [file mcaf061_suppl_supplementary_figures_s4.pdf]

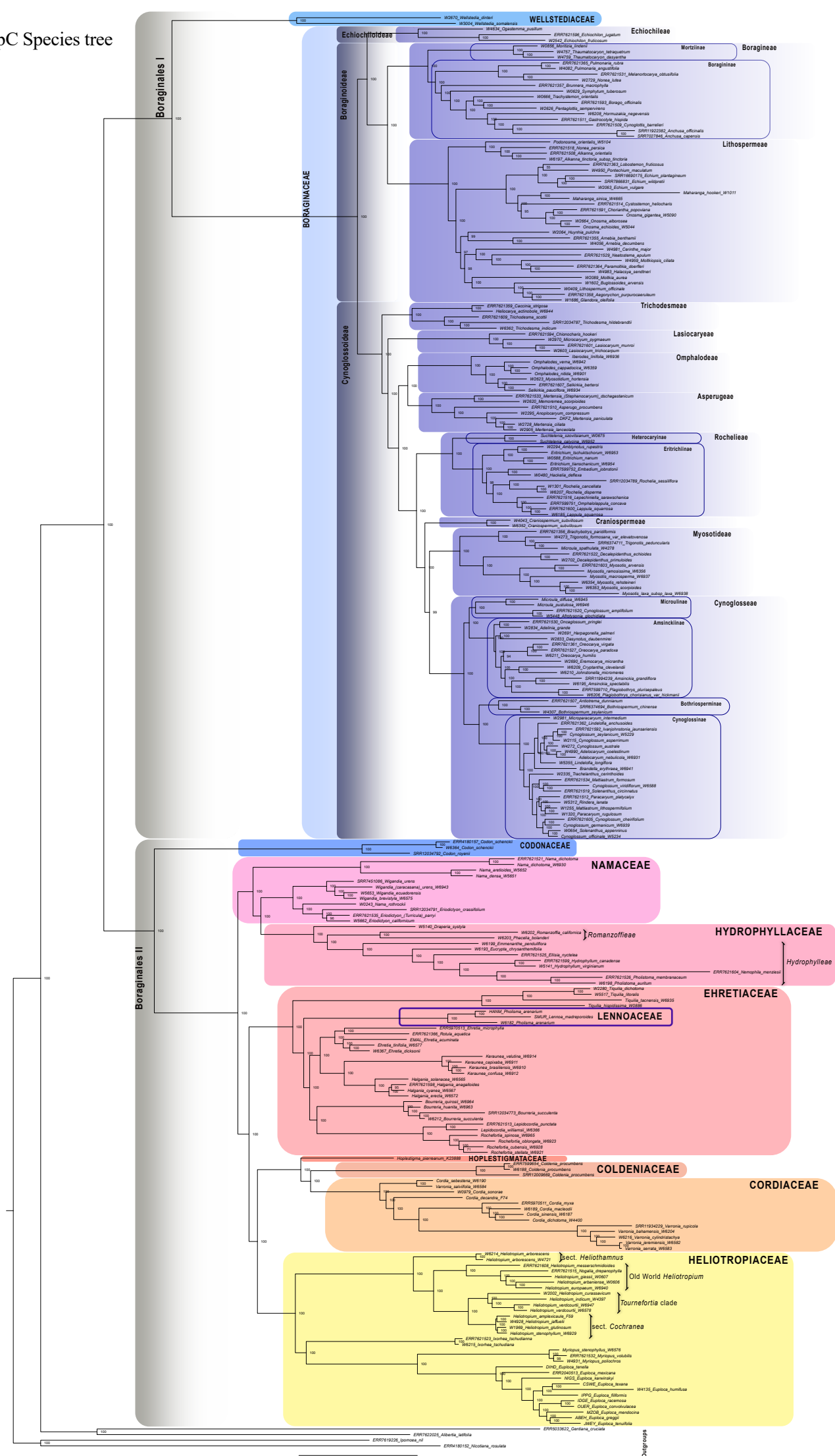

Supplement: mcaf061_suppl_Supplementary_Figures_S5 [file mcaf061_suppl_supplementary_figures_s5.pdf]
