# Supplementary material for: An updated phylogeny of Boraginales based on the Angiosperms353 probe set: a roadmap for understanding morphological evolution
Source: Ann Bot. 2025 Apr 10;136(1):77–97. doi: 10.1093/aob/mcaf061 (PMC12401892; doi:10.1093/aob/mcaf061)
Supplement: mcaf061_suppl_Supplementary_Figures_S6 [file mcaf061_suppl_supplementary_figures_s6.pdf]

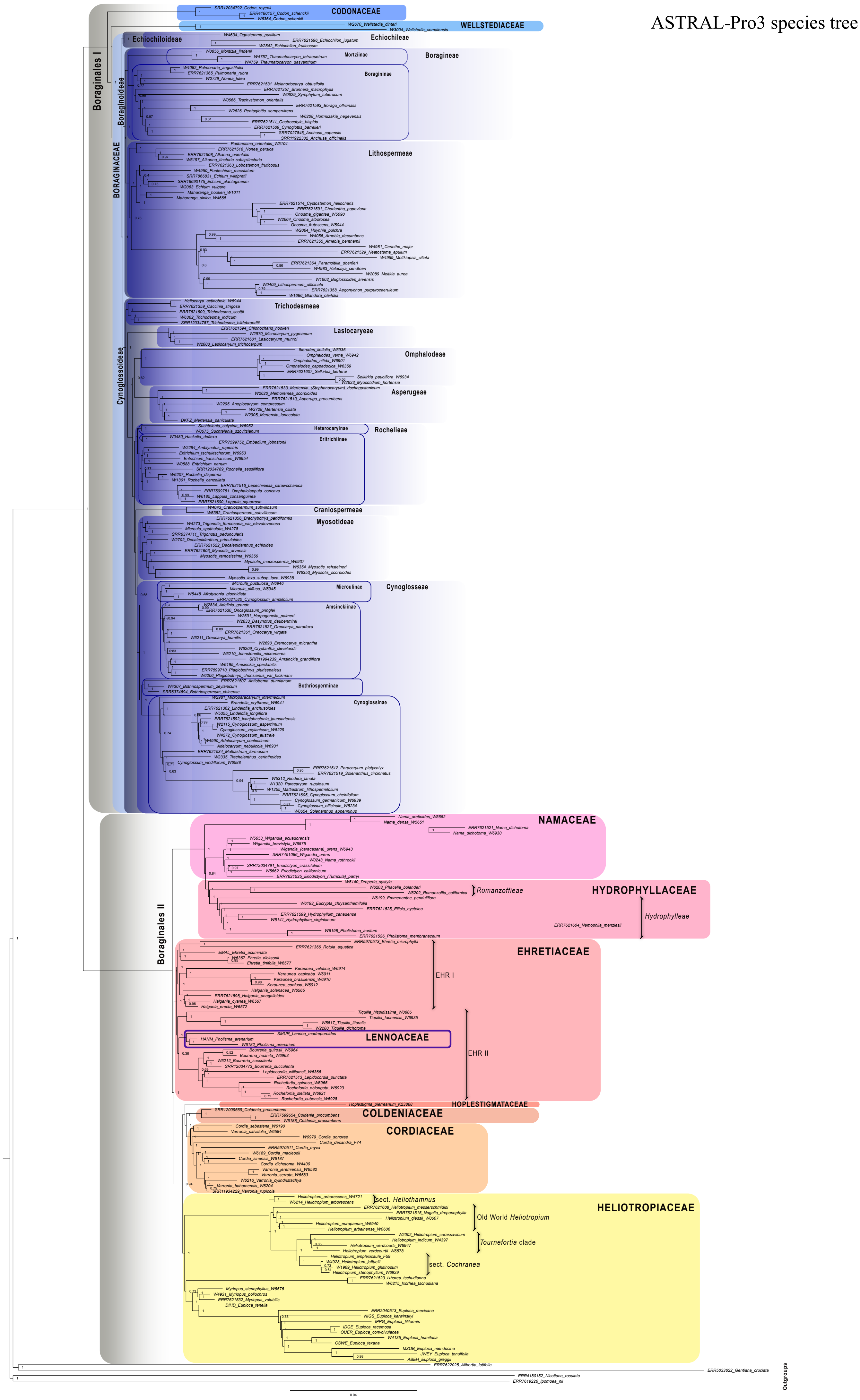
ASTRAL-Pro3 species tree
CODONACEAE
WELLSTEDIACEAE
Boraginales I
BORAGINACEAE
Echiochiloideae
Echiochileae
Boraginoideae
Boragineae
Mortziinae
Boragininae
Lithospermeae
Cynoglossoideae
Trichodesmeae
Lasiocaryeae
Omphalodeae
Asperugeae
Rochelieae
Heterocaryinae
Eritrichiinae
Craniospermeae
Myosotideae
Cynoglosseae
Microulinae
Amsinckiinae
Bothriosperminae
Cynoglossinae
Boraginales II
NAMACEAE
HYDROPHYLLACEAE
Romanzoffieae
Hydrophylleae
EHRETIACEAE
EHR I
EHR II
LENNOACEAE
HOPLESTIGMATACEAE
COLDENIACEAE
CORDIACEAE
HELIOTROPIACEAE
sect. Heliothamnus
Old World Heliotropium
Tournefortia clade
sect. Cochranea
Outgroups
0.04

Effect of paralogy on phylogenetic reconstruction

The ASTRAL-Pro3 analysis utilized all available multi-copy and single copy genes and yielded overall a very similar species tree in terms of topology and support values to that of the MuLo-Exn species tree (figs. 2 & 3), but with generally shorter branches. There are some major differences in Boraginaceae, such as the retrieval of Omphalodeae + Asperugeae with moderate support (LPP = 0.62), the improved resolution of the Lithospermeae backbone and in the lower resolution of the Cynoglossoideae backbone at tribal level (Myosotideae sister to Cynoglosseae, LPP = 0.65) and at subtribal level (Microulinae sister to Amsinckiinae, LPP = 0.67). *Microparacaryum intermedium* is retrieved as sister to the remaining Cynoglossinae with moderate support (LPP = 0.74). Within Boraginales II, in the second clade of Ehretiaceae, *Tiquilia* is successive sister to Lennoaceae with maximum support and Lennoaceae sister to the *Bourreria* + *Lepidocordia* + *Rochefortia* but with low support (LPP =0.36). Finally, within Heliotropiaceae, *Ixhorea* is retrieved as sister to *Myriopus* + *Euploca* (LPP = 0.73), a topology that was retrieved also from the concatenation analyses of both datasets (exons and the supercontigs).
