# Supplementary material for: An updated phylogeny of Boraginales based on the Angiosperms353 probe set: a roadmap for understanding morphological evolution
Source: Ann Bot. 2025 Apr 10;136(1):77–97. doi: 10.1093/aob/mcaf061 (PMC12401892; doi:10.1093/aob/mcaf061)
Supplement: mcaf061_suppl_Supplementary_Tables_S1 [file mcaf061_suppl_supplementary_tables_s1.docx]

TABLE S1. *Species included in this study and voucher information.*

| Family | Subfamily | Tribe | Subtribe | Species | Geographical Origin | Voucher | Bonn DNA Nr. | Accession | Project / Reference |
| --- | --- | --- | --- | --- | --- | --- | --- | --- | --- |
| Boraginaceae | Boraginoideae | Boragineae | Boragininae | *Anchusa capensis* Thub. | China; Nanjing | N/A |  | [SRR7027846](https://www.ebi.ac.uk/ena/browser/view/SRR7027846) | PRJNA415507 Anchusa capensis |
| Boraginaceae | Boraginoideae | Boragineae | Boragininae | *Anchusa officinalis* L. | N/A | H. Ma L18 (FUS) |  | [SRR11922382](https://www.ebi.ac.uk/ena/browser/view/SRR11922382) | Zhang et al., 2020 |
| Boraginaceae | Boraginoideae | Boragineae | Boragininae | *Borago officinalis* L. | N/A | M.W. Chase 2746 (K) |  | [ERR7621593](https://www.ebi.ac.uk/ena/browser/view/ERR7621593) | PAFTOL |
| Boraginaceae | Boraginoideae | Boragineae | Boragininae | *Brunnera macrophylla* (Adams) I.M.Johnst. | N/A | s.n.; 1948-24801 (K) |  | [ERR7621357](https://www.ebi.ac.uk/ena/browser/view/ERR7621357) | PAFTOL |
| Boraginaceae | Boraginoideae | Boragineae | Boragininae | *Cynoglottis barrelieri* (All.) Vural & Kit Tan | North Macedonia; Rosna | E. Mayer & T. Wraber  39308 (K); K001393221 | | [ERR7621509](https://www.ebi.ac.uk/ena/browser/view/ERR7621509) | PAFTOL |
| Boraginaceae | Boraginoideae | Boragineae | Boragininae | *Gastrocotyle hispida* (Forssk.) Bunge | N/A | K. Abulaila et al.  2009JOR16-2 (K); K001393223 | | [ERR7621511](https://www.ebi.ac.uk/ena/browser/view/ERR7621511) | PAFTOL |
| Boraginaceae | Boraginoideae | Boragineae | Boragininae | *Melanortocarya obtusifolia* (Willd.) Selvi, Bigazzi, Hilger & Papini | Greece; Mt. Parnitha | s.n. (K); K001275692 |  | [ERR7621531](https://www.ebi.ac.uk/ena/browser/view/ERR7621531) | PAFTOL |
| Boraginaceae | Boraginoideae | Boragineae | Boragininae | *Pulmonaria rubra* Schott | N/A | s.n.; 1994-1200 (K) |  | [ERR7621365](https://www.ebi.ac.uk/ena/browser/view/ERR7621365) | PAFTOL |
| Boraginaceae | Boraginoideae | Boragineae | Boragininae | *Hormuzakia negevensis* (Danin) Danin & Hilger | Israel; Philistean Plain | T. Faraj s.n. BONN3202 | W6208 | ERS23813090 | Present work |
| Boraginaceae | Boraginoideae | Boragineae | Boragininae | *Nonea lutea* (Desr.) DC. | N/A | M. Ackermann 702 (B); B100360718 | W2729 | ERS23813091 | Present work |
| Boraginaceae | Boraginoideae | Boragineae | Boragininae | *Pentaglottis sempervirens* (L.) Tausch ex L.H.Bailey | N/A | M. Weigend 9065 (B) | W2626 | ERS23813092 | Present work |
| Boraginaceae | Boraginoideae | Boragineae | Boragininae | *Pulmonaria angustifolia* L. | N/A | M. Ackermann 1031 (BONN) | W4082 | ERS23813093 | Present work |
| Boraginaceae | Boraginoideae | Boragineae | Boragininae | *Symphytum tuberosum* L. | Germany | H.H. Hilger s.n. (B); B100360153 | W0629 | ERS23813094 | Present work |
| Boraginaceae | Boraginoideae | Boragineae | Boragininae | *Trachystemon oriental*is (L.) D.Don | N/A | H.H. Hilger s.n. (B) | W0666 | ERS23813095 | Present work |
| Boraginaceae | Boraginoideae | Boragineae | Mortziinae | *Moritzia lindenii* (A.DC.) Benth. ex Gürke | Peru; Panao | M. Weigend et al. 5436 (USM) | W0856 | ERS23813096 | Present work |
| Boraginaceae | Boraginoideae | Boragineae | Mortziinae | *Thaumatocaryuon dasyanthum* Weigend et al. | Brazil; Uribici | R. Trevisan 1717 (BONN) | W4759 | ERS23813097 | Present work |
| Boraginaceae | Boraginoideae | Boragineae | Mortziinae | *Thaumatocaryon tetraquetrum* (Cham.) I.M.Johnst. | Brazil; Palmas | R. Trevisan 788 (BONN) | W4757 | ERS23813098 | Present work |
| Boraginaceae | Boraginoideae | Boragineae |  | *Nonea persica* Boiss. | Iraq; Sarsang to Sanrona Tuka | F.K.H. Hamid & M. Jasi  41061 (K); K001275669 | | [ERR7621518](https://www.ebi.ac.uk/ena/browser/view/ERR7621518) | PAFTOL |
| Boraginaceae | Boraginoideae | Lithospermeae |  | *Aegonychon purpurocaeruleum* (L.) Holub | N/A | M.W. Chase 6055 (K) |  | [ERR7621358](https://www.ebi.ac.uk/ena/browser/view/ERR7621358) | PAFTOL |
| Boraginaceae | Boraginoideae | Lithospermeae |  | *Alkanna orientalis* (L.) Boiss. | Turkey | Davis 42140 (K) |  | [ERR7621508](https://www.ebi.ac.uk/ena/browser/view/ERR7621508) | PAFTOL |
| Boraginaceae | Boraginoideae | Lithospermeae |  | *Arnebia benthamii* (Wall. ex G.Don) I.M.Johnst. | N/A | M.W. Chase 34887 (K) |  | [ERR7621355](https://www.ebi.ac.uk/ena/browser/view/ERR7621355) | PAFTOL |
| Boraginaceae | Boraginoideae | Lithospermeae |  | *Cystostemon heliocharis* (S.Moore) A.G.Mill. & Riedl | Yemen; South of Huth | J.R.I. Wood 1606 (K); K001275666 |  | [ERR7621514](https://www.ebi.ac.uk/ena/browser/view/ERR7621514) | PAFTOL |
| Boraginaceae | Boraginoideae | Lithospermeae |  | *Echium plantagineum* L. | Portugal; Quinta do Marques,  Oeiras | J. Capelo et al., 96329 (LISE) |  | [SRR16690175](https://trace.ncbi.nlm.nih.gov/Traces/?view=run_browser&acc=SRR16690175&display=download) | PAFTOL |
| Boraginaceae | Boraginoideae | Lithospermeae |  | *Echium wildpretii* H.Pearson ex Hook.f. | N/A | N/A |  | [SRR7866831](https://www.ebi.ac.uk/ena/browser/view/SRR7866831) | PAFTOL |
| Boraginaceae | Boraginoideae | Lithospermeae |  | *Lobostemon fruticosus* (L.) H.Buek | N/A | s.n.; 2002-438 (K) |  | [ERR7621363](https://www.ebi.ac.uk/ena/browser/view/ERR7621363) | PAFTOL |
| Boraginaceae | Boraginoideae | Lithospermeae |  | *Maharanga hookeri* (C.B.Clarke) L.Cecchi & Hilger | Tibet; South of Lhasa | W. Obermayer 08431 (GZU) | W1011 | ERS23813099 | Present work |
| Boraginaceae | Boraginoideae | Lithospermeae |  | *Maharanga sinica* (Diels) L.Cecchi & Hilger | China; Xiaojin Xia | D.E. Boufford et al. 38228, (B), (HUH) | W4665 | ERS23813100 | Present work |
| Boraginaceae | Boraginoideae | Lithospermeae |  | *Neatostema apulum* (L.) I.M.Johnst. | Turkey; West of Antalya | T.A. Tengwall 491 (K); K001275688 |  | [ERR7621529](https://www.ebi.ac.uk/ena/browser/view/ERR7621529) | PAFTOL |
| Boraginaceae | Boraginoideae | Lithospermeae |  | *Onosma frutescens* Lam. | Italy; Rivoli Veronese | L. Cecchi & F. Selvi 16.32 (FI) | W5044 | ERS23813101 | Present work |
| Boraginaceae | Boraginoideae | Lithospermeae |  | *Onosma gigantea* var*. hispida* Boiss. | Turkey; Konya | L. Cecchi et al. 13.35 (FI) | W5090 | ERS23813102 | Present work |
| Boraginaceae | Boraginoideae | Lithospermeae |  | *Onosma popoviana* (Riedl) L.Cecchi & Hilger | Iraq; NE of Rania | A. Rawi & I. Serhang 23818 (K); K000450574 | | [ERR7621591](https://www.ebi.ac.uk/ena/browser/view/ERR7621591) | PAFTOL |
| Boraginaceae | Boraginoideae | Lithospermeae |  | *Paramoltkia doerfleri* (Wettst.) Greuter & Burdet | N/A | M.W. Chase 38130.19603 (K) |  | [ERR7621364](https://www.ebi.ac.uk/ena/browser/view/ERR7621364) | PAFTOL |
| Boraginaceae | Boraginoideae | Lithospermeae |  | *Podonosma orientalis* (L.) Feinbrun | Syria; Palmira | L. Cecchi et al. 07.16 (FI) | W5104 | ERS23813103 | Present work |
| Boraginaceae | Boraginoideae | Lithospermeae |  | *Alkanna tinctoria* Tausch subsp. *tinctoria* | France; Mourèzes | s.n.; BONN2664 | W6197 | ERS23813104 | Present work |
| Boraginaceae | Boraginoideae | Lithospermeae |  | *Arnebia decumbens* (Vent.) Coss. & Kralik | Kazakhstan; Karaganda Region | А.Yu. Korolyuk & E.A. Korolyuk (NSK) | W4056 | ERS23813105 | Present work |
| Boraginaceae | Boraginoideae | Lithospermeae |  | *Buglossoides arvensis* (L.) I.M.Johnst. subsp. *arvensis* | Germany | M. Weigend & K. Weigend 2000 (B, NY, M) | W1602 | ERS23813106 | Present work |
| Boraginaceae | Boraginoideae | Lithospermeae |  | *Cerinthe major* L. | N/A | BONN1485 | W4981 | ERS23813107 | Present work |
| Boraginaceae | Boraginoideae | Lithospermeae |  | *Echium vulgare* L. | Germany | O. Mohr 597 (B); B100267626 | W2063 | ERS23813108 | Present work |
| Boraginaceae | Boraginoideae | Lithospermeae |  | *Glandora oleifolia* (Lapeyr.) D.C.Thomas | N/A | H.H. Hilger s.n. (B) | W1686 | ERS23813109 | Present work |
| Boraginaceae | Boraginoideae | Lithospermeae |  | *Halacsya sendtneri* (Boiss.) Dörfl. | Albania; Kükes Region | L. Cecchi et al. s.n. (BONN) | W4983 | ERS23813110 | Present work |
| Boraginaceae | Boraginoideae | Lithospermeae |  | *Huynhia pulchra* (Willd. ex Roem. & Schult.) Greuter & Burdet | N/A | O. Mohr 587 (B) | W2064 | ERS23813111 | Present work |
| Boraginaceae | Boraginoideae | Lithospermeae |  | *Lithospermum officinale* L. | Germany | A. Werres & M. Ristow s.n. (B) | W0409 | ERS23813112 | Present work |
| Boraginaceae | Boraginoideae | Lithospermeae |  | *Moltkia aurea* Boiss. | lbania | M.Bigazzi & F. Selvi 02.14 (B); B100327015 | W2089 | ERS23813113 | Present work |
| Boraginaceae | Boraginoideae | Lithospermeae |  | *Moltkiopsis ciliata* (Forssk.) I.M.Johnst. | Morocco; Meknès-Tafilalet | T. Joßberger TJ-321 (BONN) | W4959 | ERS23813114 | Present work |
| Boraginaceae | Boraginoideae | Lithospermeae |  | *Onosma alborosea* Fisch. & C.A.Mey. | N/A | s.n. (Bot. Garten 19(7)4-16-74-80 (B)); B100360731 | W2664 | ERS23813115 | Present work |
| Boraginaceae | Boraginoideae | Lithospermeae |  | *Pontechium maculatum* (L.) Böhle & Hilger | N/A | T. Joßberger s.n. (BONN) | W4950 | ERS23813116 | Present work |
| Boraginaceae | Cynoglossoideae | Asperugeae |  | *Anoplocaryum compressum* Ledeb. | Mongolia | H.H. Hilger 1607 (B) | W2295 | ERS23813117 | Present work |
| Boraginaceae | Cynoglossoideae | Asperugeae |  | *Asperugo procumbens* L. | Armenia; NE Artabuynk | G. Fayvush & E. Vitek 09-0644 (K); K001393222 |  | [ERR7621510](https://www.ebi.ac.uk/ena/browser/view/ERR7621510) | PAFTOL |
| Boraginaceae | Cynoglossoideae | Asperugeae |  | *Memoremea scorpioides* (Haenke) A.Otero, Jim.Mejías, Valcárcel & P.Vargas | Germany; Bayern | M. Weigend 9039 (B) | W2620 | ERS23813118 | Present work |
| Boraginaceae | Cynoglossoideae | Asperugeae |  | *Mertensia ciliata* (E.James ex Torr.) G.Don | USA | M. Ackermann 709 (B) | W2728 | ERS23813119 | Present work |
| Boraginaceae | Cynoglossoideae | Asperugeae |  | *Mertensia dschagastanica* Regel | Uzbekistan; Chodsha-barku | V. Botschantzev & A. Vvedensky 6422 (K); K001275694 | | [ERR7621533](https://www.ebi.ac.uk/ena/browser/view/ERR7621533) | PAFTOL |
| Boraginaceae | Cynoglossoideae | Asperugeae |  | *Mertensia lanceolata* (Pursh) DC. | USA | M. Weigend 9171 (B) | W2905 | ERS23813120 | Present work |
| Boraginaceae | Cynoglossoideae | Asperugeae |  | *Mertensia paniculata* (Aiton) G.Don | N/A | 132747 (ALTA) |  | [ERR2040526 = DKFZ](https://datacommons.cyverse.org/browse/iplant/home/shared/commons_repo/curated/oneKP_capstone_2019/transcript_assemblies/DKFZ-Mertensia_paniculata) | OneKP |
| Boraginaceae | Cynoglossoideae | Craniospermeae |  | *Craniospermum subvillosum* Lehm. | Russia; lake Baikal | А.Yu. Korolyuk & N. Dulepova 741 (NSK) | W6352 | ERS23813121 | Present work |
| Boraginaceae | Cynoglossoideae | Craniospermeae |  | *Craniospermum subvillosum* Lehm. | Mongolia; Chovd | А.Yu. Korolyuk s.n. (NSK) | W4043 | ERS23813122 | Present work |
| Boraginaceae | Cynoglossoideae | Cynoglosseae | Amsinckiinae | *Adelinia grandis* (Douglas ex Lehm.) J.I.Cohen | USA | M. Schwarzländer KW_891(B) | W2834 | ERS23813123 | Present work |
| Boraginaceae | Cynoglossoideae | Cynoglosseae | Amsinckiinae | *Amsinckia grandiflora* (A.Gray) Kleeb ex Greene | N/A | H. Ma HM1610 (FUS) |  | [SRR11994239](https://www.ebi.ac.uk/ena/browser/view/SRR11994239) | Zhang et al., 2020 |
| Boraginaceae | Cynoglossoideae | Cynoglosseae | Amsinckiinae | *Amsinckia spectabilis* Fisch. & C.A.Mey. | USA; Washington | T. Joßberger BONN1266 | W6195 | ERS23813124 | Present work |
| Boraginaceae | Cynoglossoideae | Cynoglosseae | Amsinckiinae | *Cryptantha clevelandii* Greene | N/A | s.n.; BONN3205 | W6209 | ERS23813125 | Present work |
| Boraginaceae | Cynoglossoideae | Cynoglosseae | Amsinckiinae | *Dasynotus daubenmirei* I.M.Johnst. | USA | M. Schwarzländer KW_889 (B) | W2833 | ERS23813126 | Present work |
| Boraginaceae | Cynoglossoideae | Cynoglosseae | Amsinckiinae | *Eremocarya micrantha* (Torr.) Greene | USA | M. Weigend 9058 (B) | W2690 | ERS23813127 | Present work |
| Boraginaceae | Cynoglossoideae | Cynoglosseae | Amsinckiinae | *Harpagonella palmeri* A.Gray | USA; California | M. Weigend 9056 (B) | W2691 | ERS23813128 | Present work |
| Boraginaceae | Cynoglossoideae | Cynoglosseae | Amsinckiinae | *Johnstonella micromeres* (A.Gray) Hasenstab & M.G.Simpson | N/A | s.n.; BONN3207 | W6210 | ERS23813129 | Present work |
| Boraginaceae | Cynoglossoideae | Cynoglosseae | Amsinckiinae | *Oncaglossum pringlei* (Greenm.) Sutorý | Mexico; near Tapalpa | s.n. (K); K001275690 |  | [ERR7621530](https://www.ebi.ac.uk/ena/browser/view/ERR7621530) | PAFTOL |
| Boraginaceae | Cynoglossoideae | Cynoglosseae | Amsinckiinae | *Oreocarya humilis* (A.Gray) Greene | N/A | s.n. (45916 BG Bonn) | W6211 | ERS23813130 | Present work |
| Boraginaceae | Cynoglossoideae | Cynoglosseae | Amsinckiinae | *Oreocarya paradoxa* A.Nelson | USA; Colorado | C.R. Broome 8497 (K);  K001275683 | | [ERR7621527](https://www.ebi.ac.uk/ena/browser/view/ERR7621527) | PAFTOL |
| Boraginaceae | Cynoglossoideae | Cynoglosseae | Amsinckiinae | *Oreocarya virgata* (Porter) Greene | N/A | M.W. Chase 6092 (K) |  | [ERR7621361](https://www.ebi.ac.uk/ena/browser/view/ERR7621361) | PAFTOL |
| Boraginaceae | Cynoglossoideae | Cynoglosseae | Amsinckiinae | *Plagiobothrys chorisianus* var. *hickmanii* (Greene) I.M.Johnst. | N/A | s.n.; BONN3219 | W6206 | ERS23813131 | Present work |
| Boraginaceae | Cynoglossoideae | Cynoglosseae | Amsinckiinae | *Plagiobothrys plurisepal***e**us (F.Muell.) I.M.Johnst. | Australia | AD 99827150 |  | [ERR7599710](https://www.ebi.ac.uk/ena/browser/view/ERR7599710) | Genomics for Australian Plants (GAP) |
| Boraginaceae | Cynoglossoideae | Cynoglosseae | Bothriosperminae | *Antiotrema dunnianum* (Diels) Hand.-Mazz. | N/A | s.n.; 36031 (K) |  | [ERR7621507](https://www.ebi.ac.uk/ena/browser/view/ERR7621507) | PAFTOL |
| Boraginaceae | Cynoglossoideae | Cynoglosseae | Bothriosperminae | *Bothriospermum chinense* Bunge | China; Shanghai | N/A |  | [SRR6374694](https://www.ebi.ac.uk/ena/browser/view/SRR6374694) | Bioproject PRJNA421868 |
| Boraginaceae | Cynoglossoideae | Cynoglosseae | Bothriosperminae | *Bothriospermum zeylanicum* (J.Jacq.) Druce | Japan; Tanegashima Division | T. Joßberger s.n. (BONN) | W4307 | ERS23813132 | Present work |
| Boraginaceae | Cynoglossoideae | Cynoglosseae | Cynoglossinae | *Adelocaryum nebulicola* R.R.Mill | Oman; Dhofar | A. Miller 7202; E (E00699269) | W6931 | ERS23813133 | Present work |
| Boraginaceae | Cynoglossoideae | Cynoglosseae | Cynoglossinae | *Brandella erythraea* (Brand) R.R.Mill | Saudi Arabia; Taif Jeddah road | I. Collenette 1707; E (E00843476) | W6941 | ERS23813134 | Present work |
| Boraginaceae | Cynoglossoideae | Cynoglosseae | Cynoglossinae | *Cynoglossum germanicum* Jacq. | France; Soultz-Haut-Rhin | A. Kirchberg; BONN2963 | W6939 | ERS23813135 | Present work |
| Boraginaceae | Cynoglossoideae | Cynoglosseae | Cynoglossinae | *Cynoglossum officinale* L. | Germany; NRW Iversheim | T. Joßberger TJ-369 | W5234 | ERS23813136 | Present work |
| Boraginaceae | Cynoglossoideae | Cynoglosseae | Cynoglossinae | *Cynoglossum zeylanicum* (Sw. ex Lehm.) Thunb. ex Brand | Nepal; Likhu Khola | J.D. Stainton 4640 (BM) | W5229 | ERS23813137 | Present work |
| Boraginaceae | Cynoglossoideae | Cynoglosseae | Cynoglossinae | *Adelocaryum coelestinum (Lindl.) Brand* | India; Maharashtra | N.R. Gupte 18 | W4990 | ERS23813138 | Present work |
| Boraginaceae | Cynoglossoideae | Cynoglosseae | Cynoglossinae | *Cynoglossum asperrimum* Nakai | Japan | O. Mohr 615 (B); B100360582 | W2115 | ERS23813139 | Present work |
| Boraginaceae | Cynoglossoideae | Cynoglosseae | Cynoglossinae | *Cynoglossum australe* R.Br. | Australia; Tasmania | T. Joßberger s.n. (BONN) | W4272 | ERS23813140 | Present work |
| Boraginaceae | Cynoglossoideae | Cynoglosseae | Cynoglossinae | *Cynoglossum cheirifolium* L. | N/A | M.W. Chase 6065 (K) |  | [ERR7621605](https://www.ebi.ac.uk/ena/browser/view/ERR7621605) |  |
| Boraginaceae | Cynoglossoideae | Cynoglosseae | Cynoglossinae | *Cynoglossum viridiflorum* Pall. ex Lehm. | Kazakhstan; Dzungarian Alatau | N.J. Rubtsov (AA) | W6588 | ERS23813141 | Present work |
| Boraginaceae | Cynoglossoideae | Cynoglosseae | Cynoglossinae | *Ivanjohnstonia jaunsariensis* Kazmi | India; Kakuau | J.S. Gamble 27383 (K); K000998422 |  | [ERR7621592](https://www.ebi.ac.uk/ena/browser/view/ERR7621592) | PAFTOL |
| Boraginaceae | Cynoglossoideae | Cynoglosseae | Cynoglossinae | *Lindelofia anchusoides* (Lindl.) Lehm. | N/A | M.W. Chase 38131.19907 (K) |  | [ERR7621362](https://www.ebi.ac.uk/ena/browser/view/ERR7621362) | PAFTOL |
| Boraginaceae | Cynoglossoideae | Cynoglosseae | Cynoglossinae | *Lindelofia longiflora* (DC.) Baill. | Hungary; Vácrátót | s.n.; BONN2379 | W5355 | ERS23813142 |  |
| Boraginaceae | Cynoglossoideae | Cynoglosseae | Cynoglossinae | *Mattiastrum formosum* Rech.f. & Riedl | Afghanistan; Salang-Tal | D. Podlech 18096 (K); K001275695 |  | [ERR7621534](https://www.ebi.ac.uk/ena/browser/view/ERR7621534) | PAFTOL |
| Boraginaceae | Cynoglossoideae | Cynoglosseae | Cynoglossinae | *Mattiastrum lithospermifolium* (Lam.) Brand | Greece | H.H. Hilger s.n. (B); B100844769 | W1255 | ERS23813143 | Present work |
| Boraginaceae | Cynoglossoideae | Cynoglosseae | Cynoglossinae | *Microparacaryum intermedium* (Fresen.) Hilger & Podlech | N/A | H. Kürschner 10-52 (Herbarium Kürschner) | W2981 | ERS23813144 | Present work |
| Boraginaceae | Cynoglossoideae | Cynoglosseae | Cynoglossinae | *Paracaryum platycalyx* Riedl | Iran; Khorasan | K.H. Rechinger 57545 (K); K001275663 |  | [ERR7621512](https://www.ebi.ac.uk/ena/browser/view/ERR7621512) |  |
| Boraginaceae | Cynoglossoideae | Cynoglosseae | Cynoglossinae | *Paracaryum rugulosum* (DC.) Boiss. | Turkey; Gürün | N/A | W1320 | ERS23813145 | Present work |
| Boraginaceae | Cynoglossoideae | Cynoglosseae | Cynoglossinae | *Rindera lanata* (Lam.) Bunge | Iran; Khomein | Y. Salmaki & S. Zarre 47363 (TUH) | W5312 | ERS23813146 | Present work |
| Boraginaceae | Cynoglossoideae | Cynoglosseae | Cynoglossinae | *Solenanthus apenninus* (L.) Fisch. & C.A.Mey. | Italy | W. Frey s.n. (B); B100844757 | W0654 | ERS23813147 | Present work |
| Boraginaceae | Cynoglossoideae | Cynoglosseae | Cynoglossinae | *Solenanthus circinnatus* Ledeb. | Iran; Khorasan | K.H. Rechinger 53423 (K); K001275671 |  | [ERR7621519](https://www.ebi.ac.uk/ena/browser/view/ERR7621519) | PAFTOL |
| Boraginaceae | Cynoglossoideae | Cynoglosseae | Cynoglossinae | *Trachelanthus cerinthoides* (Boiss.) Kunze | N/A | M.R. Parishani 14275 (M) | W2335 | ERS23813148 | Present work |
| Boraginaceae | Cynoglossoideae | Cynoglosseae | Microulinae | *Microula diffusa* (Maxim.) I.M.Johnst. | China | T.N. Ho et al. 2558 (BONN) | W6945 | ERS23813149 | Present work |
| Boraginaceae | Cynoglossoideae | Cynoglosseae | Microulinae | *Microula pustulosa* (C.B.Clarke) Duthie | China; Qinghai | T.N. Ho et al. 2328 (E) | W6946 | ERS23813150 | Present work |
| Boraginaceae | Cynoglossoideae | Cynoglosseae | Microulinae | *Afrotysonia glochidiata* (R.R.Mill) R.Mill | Republic of South Africa, Mondi’s Donnybrook Estate | D.G.A. Styles 5700 (BONN) | W5448 | ERS23813151 | Present work |
| Boraginaceae | Cynoglossoideae | Cynoglosseae | Microulinae | *Cynoglossum amplifolium* Hochst. ex A.DC. | Tanzania | S. Bidgood & P. Keeley 288 (K);  K001275673 | | [ERR7621520](https://www.ebi.ac.uk/ena/browser/view/ERR7621520) | PAFTOL |
| Boraginaceae | Cynoglossoideae | Lasiocaryeae |  | *Chionocharis hookeri* (C.B.Clarke) I.M.Johnst. | Nepal; Above Lhonak | Crawford et al. 571 (K) |  | [ERR7621594](https://www.ebi.ac.uk/ena/browser/view/ERR7621594) | PAFTOL |
| Boraginaceae | Cynoglossoideae | Lasiocaryeae |  | *Lasiocaryum densiflorum* (Duthie) I.M.Johnst. | N/A | I.W.J. Sinclair & D.G. Long 5405 (E) | W2603 | ERS23813152 | Present work |
| Boraginaceae | Cynoglossoideae | Lasiocaryeae |  | *Lasiocaryum munroi* (C.B.Clarke) I.M.Johnst. | China; Shigatse | Cutting & Vernay 81 (K) |  | [ERR7621601](https://www.ebi.ac.uk/ena/browser/view/ERR7621601) | PAFTOL |
| Boraginaceae | Cynoglossoideae | Lasiocaryeae |  | *Microcaryum pygmaeum* I.M.Johnst. | China; Sichuan | D.E. Boufford et al. 36208 (B, HUH) | W2970 | ERS23813153 | Present work |
| Boraginaceae | Cynoglossoideae | Myosotideae |  | *Microula spathulata* W.T.Wang | China; Sichuan | D.E. Boufford 33531 (B, HUH, P) | W4278 | ERS23813154 | Present work |
| Boraginaceae | Cynoglossoideae | Myosotideae |  | *Myosotis laxa* Lehm. subsp. *laxa* | USA; NY, Guggenheim lakes | M. Bennett 9210 (B) | W6938 | ERS23813155 | Present work |
| Boraginaceae | Cynoglossoideae | Myosotideae |  | *Myosotis macrosperma* Engelm. | USA; Carolina | J.B. Nelson 30352 (B) | W6937 | ERS23813156 | Present work |
| Boraginaceae | Cynoglossoideae | Myosotideae |  | *Myosotis ramosissima* Rochel ex Schult. | Germany; NRW, Königswinter | S. Abrahamczyk (BONN) | W6356 | ERS23813157 | Present work |
| Boraginaceae | Cynoglossoideae | Myosotideae |  | *Myosotis rehsteineri* (Hausm.) Wartm. ex Reut. | N/A | s.n.; BONN577 | W6354 | ERS23813158 | Present work |
| Boraginaceae | Cynoglossoideae | Myosotideae |  | *Myosotis scorpioides* L. | Germany; NRW, Bonn | s.n.; BONN2988 | W6353 | ERS23813159 | Present work |
| Boraginaceae | Cynoglossoideae | Myosotideae |  | *Brachybotrys paridiformis* Maxim. ex Oliv. | N/A | E.A. Pimenova s.n.; 88112 (K) |  | [ERR7621356](https://www.ebi.ac.uk/ena/browser/view/ERR7621356) | PAFTOL |
| Boraginaceae | Cynoglossoideae | Myosotideae |  | *Decalepidanthus echioides* (Royle ex Benth.) Dickoré & Hilger | Pakistan; Kargah valley. | S.B. Lyon 8014 (K); K001275678 |  | [ERR7621522](https://www.ebi.ac.uk/ena/browser/view/ERR7621522) | PAFTOL |
| Boraginaceae | Cynoglossoideae | Myosotideae |  | *Decalepidanthus primuloides* (Decne.) Dickoré & Hilger | N/A | s.n.; 19751894 (E) | W2702 | ERS23813160 | Present work |
| Boraginaceae | Cynoglossoideae | Myosotideae |  | *Myosotis arvensis* (L.) Hill | N/A | M.W. Chase 6057 (K) |  | [ERR7621603](https://www.ebi.ac.uk/ena/browser/view/ERR7621603) |  |
| Boraginaceae | Cynoglossoideae | Myosotideae |  | *Trigonotis formosana* var. *elevatovenosa (Hayata)* S.D.Shen & J.C.Wang | Taiwan; Ilan County | M. Weigend 8128 (BONN) | W4273 | ERS23813161 | Present work |
| Boraginaceae | Cynoglossoideae | Myosotideae |  | *Trigonotis peduncularis* (Trevir.) Benth. ex F.B.Forbes & Hemsl. | China; Shanghai | N/A |  | [SRR6374711](https://www.ebi.ac.uk/ena/browser/view/SRR6374711) | Bioproject PRJNA421868 |
| Boraginaceae | Cynoglossoideae | Omphalodeae |  | *Iberodes linifolia* (L.) M.Serrano, R.Carbajal & S.Ortiz | N/A | M. Ackermann 712 (B); B100360905 | W6936 | ERS23813162 | Present work |
| Boraginaceae | Cynoglossoideae | Omphalodeae |  | *Omphalodes cappadocica* (Willd.) DC. | Russia; Sotschi | F. Trabert s.n. BONN973 | W6359 | ERS23813163 | Present work |
| Boraginaceae | Cynoglossoideae | Omphalodeae |  | *Omphalodes nitida* (Willd.) Hoffmanns. & Link | N/A | BONN3768 | W6901 | ERS23813164 | Present work |
| Boraginaceae | Cynoglossoideae | Omphalodeae |  | *Omphalodes verna* Moench | N/A | H.H. Hilger (B) | W6942 | ERS23813165 | Present work |
| Boraginaceae | Cynoglossoideae | Omphalodeae |  | *Selkirkia pauciflora* (Ruiz & Pav.) Holstein & Weigend | Chile | T. Kern 23 (B) | W6934 | ERS23813166 | Present work |
| Boraginaceae | Cynoglossoideae | Omphalodeae |  | *Myosotidium hortensia* (Decne.) Baill. | New Zealand; Chatham Is | M. Weigend 9068 (B) | W2623 | ERS23813167 | Present work |
| Boraginaceae | Cynoglossoideae | Omphalodeae |  | *Selkirkia berteroi* (Colla) Hemsl. | Chile; Masatiera | O.T. Solbrig 3807 (K) |  | [ERR7621607](https://www.ebi.ac.uk/ena/browser/view/ERR7621607) | PAFTOL |
| Boraginaceae | Cynoglossoideae | Rochelieae | Eritrichiinae | *Eritrichium tianschanicum* Iljin ex Ovczinnikova | Kazakhstan; Kegen | B. Schischkin (LE) | W6954 | ERS23813168 | Present work |
| Boraginaceae | Cynoglossoideae | Rochelieae | Eritrichiinae | *Eritrichium tschuktschorum* Jurtzev & V.V.Petrovsky | Russia; Magadan | T.N. Antropova & A.P. Chochrjakov s.n. (NSK) | W6953 | ERS23813169 | Present work |
| Boraginaceae | Cynoglossoideae | Rochelieae | Eritrichiinae | *Amblynotus rupestris* (Georgi) Popov | N/A | H.H. Hilger 1597 (B); B100360714 | W2294 | ERS23813170 | Present work |
| Boraginaceae | Cynoglossoideae | Rochelieae | Eritrichiinae | *Embadium johnstonii* Ising | Australia | AD 251469 |  | [ERR7599752](https://www.ebi.ac.uk/ena/browser/view/ERR7599752) | Genomics for Australian Plants (GAP) |
| Boraginaceae | Cynoglossoideae | Rochelieae | Eritrichiinae | *Eritrichium nanum* (L.) Gaudin | Switzerland | E. Zippel et al. s.n. (B) | W0588 | ERS23813171 | Present work |
| Boraginaceae | Cynoglossoideae | Rochelieae | Eritrichiinae | *Hackelia deflexa* (Wahlenb.) Opiz | Switzerland | W. Frey s.n. (B) | W0480 | ERS23813172 | Present work |
| Boraginaceae | Cynoglossoideae | Rochelieae | Eritrichiinae | *Lappula squarrosa* (Retz.) Dumort. | N/A | M.W. Chase 6549 (K) |  | [ERR7621600](https://www.ebi.ac.uk/ena/browser/view/ERR7621600) | PAFTOL |
| Boraginaceae | Cynoglossoideae | Rochelieae | Eritrichiinae | *Lappula squarrosa* (Retz.) Dumort. | N/A | T. Joßberger s.n. (BONN) | W6185 | ERS23813173 | Present work |
| Boraginaceae | Cynoglossoideae | Rochelieae | Eritrichiinae | *Lepechiniella sarawschanica* (Lipsky) Popov | Kyrgyzstan; Jalal-Abad Oblast | S. Landrein 179 (K); K000493434 |  | [ERR7621516](https://www.ebi.ac.uk/ena/browser/view/ERR7621516) | PAFTOL |
| Boraginaceae | Cynoglossoideae | Rochelieae | Eritrichiinae | *Omphalolappula concava* (F.Muell.) Brand | Australia | AD 251455 |  | [ERR7599751](https://treeoflife.kew.org/specimen-viewer/23827) | Genomics for Australian Plants (GAP) |
| Boraginaceae | Cynoglossoideae | Rochelieae | Eritrichiinae | *Rochelia cancellata* Boiss. & Balansa | N/A | M. Bigazzi & F. Selvi 02.53 (FI) | W1301 | ERS23813174 | Present work |
| Boraginaceae | Cynoglossoideae | Rochelieae | Eritrichiinae | *Rochelia disperma* (L.f.) K.Koch | Israel; Mount Hermon | Y. Ur, BONN3209 | W6207 | ERS23813175 | Present work |
| Boraginaceae | Cynoglossoideae | Rochelieae | Eritrichiinae | *Rochelia sessiliflora* (Boiss.) Khoshsokhan & Kaz.Osaloo | N/A | H. Ma L55 (FUS) |  | [SRR12034789](https://www.ebi.ac.uk/ena/browser/view/SRR12034789) | Zhang et al., 2020 |
| Boraginaceae | Cynoglossoideae | Rochelieae | Heterocaryinae | *Suchtelenia calycina* (C.A.Mey.) DC. | Kazakhstan; Aral | А.Yu. Korolyuk; NSK | W6952 | ERS23813176 | Present work |
| Boraginaceae | Cynoglossoideae | Rochelieae | Heterocaryinae | *Suchtelenia szovitsiana* (Fisch. & C.A.Mey.) Sennikov | Ν/A | F. Selvi & M. Bigazzi 28.5.00 (FI) | W0675 | ERS23813177 | Present work |
| Boraginaceae | Cynoglossoideae | Trichodesmeae |  | *Heliocarya actinobole* (Bunge) Ranjbar & Khalvati | Iran; Mazandaran | M. Assadi & Jamzad 55216; TARI | W6944 | ERS23813178 | Present work |
| Boraginaceae | Cynoglossoideae | Trichodesmeae |  | *Trichodesma indicum* (L.) Sm. | India; Maharashtra | N.R. Gupte 20 | W6362 | ERS23813179 | Present work |
| Boraginaceae | Cynoglossoideae | Trichodesmeae |  | *Caccinia strigosa* Boiss. | N/A | M.W. Chase 38129.19692 (K) |  | [ERR7621359](https://www.ebi.ac.uk/ena/browser/view/ERR7621359) | PAFTOL |
| Boraginaceae | Cynoglossoideae | Trichodesmeae |  | *Trichodesma hildebrandtii* Gürke | Oman; Dhofar | H. Ma L57 (FUS) |  | [SRR12034787](https://www.ebi.ac.uk/ena/browser/view/SRR12034787) | Zhang et al., 2020 |
| Boraginaceae | Cynoglossoideae | Trichodesmeae |  | *Trichodesma scottii* Balf.f. | N/A | M.W. Chase 2912 (K) |  | [ERR7621609](https://www.ebi.ac.uk/ena/browser/view/ERR7621609) | PAFTOL |
| Boraginaceae | Echiochiloideae | Echiochileae |  | *Echiochilon jugatum* I.M.Johnst. | N/A | Gallagher 76965 (K) |  | [ERR7621596](https://www.ebi.ac.uk/ena/browser/view/ERR7621596) | PAFTOL |
| Boraginaceae | Echiochiloideae | Echiochileae |  | *Echiochilon fruticosum* Desf. | N/A | A. Kagiampaki s.n. (B); B100267677 | W2542 | ERS23813180 | Present work |
| Boraginaceae | Echiochiloideae | Echiochileae |  | *Ogastemma pusillum* (Coss. & Durieu ex Bonnet & Barratte) Brummitt | Morocco; Zagora | T. Joßberger TJH-300 (BONN) | W4634 | ERS23813181 | Present work |
| Codonaceae |  |  |  | *Codon royenii* L. | South Africa; Cape | H. Ma L52 (FUS) |  | [SRR12034792](https://www.ebi.ac.uk/ena/browser/view/SRR12034792) | Zhang et al., 2020 |
| Codonaceae |  |  |  | *Codon schenckii* Schinz | Namibia | R. Seydel 2968 (K) |  | [ERR4180157](https://www.ebi.ac.uk/ena/browser/view/ERR4180157) | PAFTOL |
| Codonaceae |  |  |  | *Codon schenckii* Schinz | Namibia; Kaokoveld | P. Lintermann s.n.; BONN653 | W6364 | ERS23813182 | Present work |
| Coldeniaceae |  |  |  | *Coldenia procumbens* L. | China; Hainan | C.F. Zhang 4466 (FUS) |  | [SRR12009669](https://www.ebi.ac.uk/ena/browser/view/SRR12009669) | Zhang et al., 2020 |
| Coldeniaceae |  |  |  | *Coldenia procumbens* L. | Australia | MEL 2441170A |  | [ERR7599654](https://www.ebi.ac.uk/ena/browser/view/ERR7599654) | Genomics for Australian Plants (GAP) |
| Coldeniaceae |  |  |  | *Coldenia procumbens* L. | India; Asangaon | N.R. Gupte NRG-12 | W6188 | ERS23813183 | Present work |
| Convolvulaceae |  |  |  | *Ipomoea nil* (L.) Roth | N/A | C.J. Leon et al. 498 (K) |  | [ERR7619226](https://www.ebi.ac.uk/ena/browser/view/ERR7619226) | PAFTOL |
| Cordiaceae |  |  |  | *Cordia decandra* Hook. & Arn. | Chile | F. Luebert & Kritzner 1873 (SGO EIF) | F74 | ERS23813184 | Present work |
| Cordiaceae |  |  |  | *Cordia dichotoma* G.Forst. | India; Maharashtra | N.R. Gupte NRG-05 | W4400 | ERS23813185 | Present work |
| Cordiaceae |  |  |  | *Cordia macleodii* (Griff.) Hook.f. & Thomson | India; Asangaon | N.R. Gupte NRG-13 | W6189 | ERS23813186 | Present work |
| Cordiaceae |  |  |  | *Cordia myxa* L. | N/A | A.J.M. Leeuwenberg 7592 (K) |  | [ERR5970511](https://www.ebi.ac.uk/ena/browser/view/ERR5970511) | PAFTOL |
| Cordiaceae |  |  |  | *Cordia sebestena* L. | India; Badlapur | N.R. Gupte NRG-14 | W6190 | ERS23813187 | Present work |
| Cordiaceae |  |  |  | *Cordia sinensis* Lam. | India; Colaba | N.R. Gupte NRG-10 | W6187 | ERS23813188 | Present work |
| Cordiaceae |  |  |  | *Cordia sonorae* Rose | USA | J.S. Miller & Campos 2956 (B); B100305541 | W0979 | ERS23813189 | Present work |
| Cordiaceae |  |  |  | *Varronia bahamensis* (Urb.) Millsp. | Bahamas, New Providence | M.A. Hamilton MAH861 (K) | W6204 | ERS23813190 | Present work |
| Cordiaceae |  |  |  | *Varronia cylindristachya* Ruiz & Pav. | Peru; Ferreñafe | Μ. Weigend et al. MJ14/147; BONN1937 | W6216 | ERS23813191 | Present work |
| Cordiaceae |  |  |  | *Varronia jeremiensis* (Urb. & Ekman) Borhidi | Dominican Republic; Pedernales | M. Weigend 9921 (BONN) | W6582 | ERS23813192 | Present work |
| Cordiaceae |  |  |  | *Varronia rupicola* (Urb.) Britton | N/A | N/A |  | [SRR11934229](https://www.ebi.ac.uk/ena/browser/view/SRR11934229) | Zhang et al., 2020 |
| Cordiaceae |  |  |  | *Varronia salviifolia* (Juss. ex Poir.) Borhidi | Dominican Republic; Pedernales | M. Weigend 9923 (BONN) | W6584 | ERS23813193 | Present work |
| Cordiaceae |  |  |  | *Varronia serrata* (L.) Borhidi | Dominican Republic; Pedernales | M. Weigend 9922 (BONN) | W6583 | ERS23813194 | Present work |
| Ehretiaceae |  |  |  | *Bourreria huanita* (Lex.) Hemsl. | Costa Rica; Puntarenas | R. Acuña et al. 3287 (USJ) | W6963 | ERS23813195 | Present work |
| Ehretiaceae |  |  |  | *Bourreria quirosii* Standl. | Costa Rica; Guanacaste | R. Acuña et al. 3322 (USJ) | W6964 | ERS23813196 | Present work |
| Ehretiaceae |  |  |  | *Bourreria succulenta* Jacq. | N/A | s.n.; BONN1298 | W6212 | ERS23813197 | Present work |
| Ehretiaceae |  |  |  | *Bourreria succulenta* Jacq*.* | N/A | H. Ma L71 (FUS) |  | [SRR12034773](https://www.ebi.ac.uk/ena/browser/view/SRR12034773) | Zhang et al., 2020 |
| Ehretiaceae |  |  |  | *Ehretia acuminata* R.Br. | N/A | Soltis and Miles 2838 |  | [ERR2040525 = EMAL](https://datacommons.cyverse.org/browse/iplant/home/shared/commons_repo/curated/oneKP_capstone_2019/transcript_assemblies/EMAL-Ehretia_acuminata) | OneKP |
| Ehretiaceae |  |  |  | *Ehretia dicksonii* Hance | N/A | s.n.; (BONN) | W6367 | ERS23813198 | Present work |
| Ehretiaceae |  |  |  | *Ehretia microphylla* Lam. | Papua New Guinea | R. Pullen 6809 (K); K000062263 |  | [ERR5970513](https://www.ebi.ac.uk/ena/browser/view/ERR5970513) | PAFTOL |
| Ehretiaceae |  |  |  | *Ehretia tinifolia* L. | Dominican Republic; Azua | M. Weigend 9915 (BONN) | W6577 | ERS23813199 | Present work |
| Ehretiaceae |  |  |  | *Halgania anagalloides* Endl. | Australia; West Perth. | R.J. Smith & A. Shade 2 (K) |  | [ERR7621598](https://www.ebi.ac.uk/ena/browser/view/ERR7621598) | PAFTOL |
| Ehretiaceae |  |  |  | *Halgania cyanea* Lindl. | Australia | G. Byrne 6051; PERTH 08936749 | W6567 | ERS23813200 | Present work |
| Ehretiaceae |  |  |  | *Halgania erecta* Ewart & B.Rees | Australia | N. Gibson et al. NG7288; PERTH 08759170 | W6572 | ERS23813201 | Present work |
| Ehretiaceae |  |  |  | *Halgania solanacea* F.Muell. | Australia | M. Goods DD 997; PERTH 08876045 | W6565 | ERS23813202 | Present work |
| Ehretiaceae |  |  |  | *Keraunea brasiliensis* Cheek & Sim.-Bianch. | Brazil; Bahia | D. Cardoso et al. 4904 (ALCB, HUEFS, RB, TCD) | W6910 | ERS23813203 | Present work |
| Ehretiaceae |  |  |  | *Keraunea capixaba* Lombardi | Brazil; Bahia | D.A. Folli 7273; CVRD [acc. #15122]; RB [01103439] | W6911 | ERS23813204 | Present work |
| Ehretiaceae |  |  |  | *Keraunea confusa* Moonlight & D.B.O.S.Cardoso | Brazil; Minas Gerais | D. Cardoso et al. 4916 (ALCB, HUEFS, RB, TCD) | W6912 | ERS23813205 | Present work |
| Ehretiaceae |  |  |  | *Keraunea velutina* Moonlight & D.B.O.S.Cardoso | Brazil; Rio de Janeiro | J.G. Costa 257; holotype: RB [00852871] | W6914 | ERS23813206 | Present work |
| Ehretiaceae |  |  |  | *Lepidocordia punctata* Ducke | Brazil; Amazônica | J. Jangoux & B.G.S. Ribeiro 1479 (K); K001275665 | | [ERR7621513](https://www.ebi.ac.uk/ena/browser/view/ERR7621513) | PAFTOL |
| Ehretiaceae |  |  |  | *Lepidocordia williamsii* (I.M.Johnst.) J.S.Mill. | Nicaragua | J.S. Miller & P.P. Moreno 1051; B100313480 | W6366 | ERS23813207 | Present work |
| Ehretiaceae |  |  |  | *Rochefortia cubensis* Britton & P.Wilson | Cuba; Guantánamo | A. Álvarez de Zayas et al. Flora de Cuba 43212; JE00028808 | W6928 | ERS23813208 | Present work |
| Ehretiaceae |  |  |  | *Rochefortia oblongata* Urb. & Ekman | Cuba; Baracoa | A. Álvarez de Zayas et al. Flora de Cuba 43678; JE00028804 | W6923 | ERS23813209 | Present work |
| Ehretiaceae |  |  |  | *Rochefortia spinosa* (Jacq.) Urb | Costa Rica; Guanacaste | R. Acuña & A. Herrera 3353 (USJ) | W6965 | ERS23813210 | Present work |
| Ehretiaceae |  |  |  | *Rochefortia stellata Britton & P.Wilson* | Cuba; Cuba; Guantánamo | J. Bisse & G. Stohr Flora de Cuba 36608; JE00028812 | W6921 | ERS23813211 | Present work |
| Ehretiaceae |  |  |  | *Rotula aquatica* Lour. | Sierra Leone | J. Momoh 130 (K); K001243255 |  | [ERR7621366](https://www.ebi.ac.uk/ena/browser/view/ERR7621366) | PAFTOL |
| Ehretiaceae |  |  |  | *Tiquilia dichotoma* (Ruiz & Pav.) Pers. | Peru | M. Weigend & C. Schwarzer 8331 (B) | W2280 | ERS23813212 | Present work |
| Ehretiaceae |  |  |  | *Tiquilia hispidissima* (Torr. & A.Gray) A.T.Richardson | USA | Howell & True 44757 (B); B100354388 | W0886 | ERS23813213 | Present work |
| Ehretiaceae |  |  |  | *Tiquilia litoralis* (Phil.) A.T.Richardson | N/A | F. Luebert et al. 3272 (BONN) | W5517 | ERS23813214 | Present work |
| Ehretiaceae |  |  |  | *Tiquilia tacnensis* A.T.Richardson | Peru; Moquegua | M. Weigend & M. Ackermann 9249 (B) | W6935 | ERS23813215 | Present work |
| Gentianaceae |  |  |  | *Gentiana cruciata* L. | N/A | 2001-3694 (K) |  | [ERR5033622](https://www.ebi.ac.uk/ena/browser/view/ERR5033622) | PAFTOL |
| Heliotropiaceae |  |  |  | *Euploca convolvulacea* Nutt. | N/A | N/A |  | [ERP023948 = OUER](https://datacommons.cyverse.org/browse/iplant/home/shared/commons_repo/curated/oneKP_capstone_2019/transcript_assemblies/OUER-Heliotropium_convolvulaceum) | OneKP |
| Heliotropiaceae |  |  |  | *Euploca filiformis* (Lehm.) J.I.M.Melo & Semir | N/A | N/A |  | [ERR2040517= OEKO](https://datacommons.cyverse.org/browse/iplant/home/shared/commons_repo/curated/oneKP_capstone_2019/transcript_assemblies/OEKO-Heliotropium_filiforme-2_samples_combined) | OneKP |
| Heliotropiaceae |  |  |  | *Euploca greggii* (Torr.) Halse & Feuillet | N/A | N/A |  | [ERR2040518 =ABEH](https://datacommons.cyverse.org/browse/iplant/home/shared/commons_repo/curated/oneKP_capstone_2019/transcript_assemblies/ABEH-Heliotropium_greggii) | OneKP |
| Heliotropiaceae |  |  |  | *Euploca humifusa* (Kunth) Diane & Hilger | Cuba; Holguín | P. Gonzalez 1306-6 (HAJB) | W4135 | ERS23813216 | Present work |
| Heliotropiaceae |  |  |  | *Euploca karwinskyi* (I.M.Johnst.) J.I.M.Melo | N/A | N/A |  | [ERR2040519=NIGS](https://datacommons.cyverse.org/browse/iplant/home/shared/commons_repo/curated/oneKP_capstone_2019/transcript_assemblies/NIGS-Heliotropium_karwinskyi) | OneKP |
| Heliotropiaceae |  |  |  | *Euploca mendocina* (Phil.) Diane & Hilger | N/A | N/A |  | [ERR2040520 = MZOB](https://datacommons.cyverse.org/browse/iplant/home/shared/commons_repo/curated/oneKP_capstone_2019/transcript_assemblies/MZOB-Heliotropium_mendocinum) | OneKP |
| Heliotropiaceae |  |  |  | *Euploca mexicana* (A.DC.) M.W.Frohl. & M.W.Chase | N/A | N/A |  | [ERR2040513= XVRU](https://datacommons.cyverse.org/browse/iplant/home/shared/commons_repo/curated/oneKP_capstone_2019/transcript_assemblies/XVRU-Heliotropium_calcicola-3_samples_combined) | OneKP |
| Heliotropiaceae |  |  |  | *Euploca racemosa* Rose & Standl. | N/A | N/A |  | [ERR2040521=IDGE](https://datacommons.cyverse.org/browse/iplant/home/shared/commons_repo/curated/oneKP_capstone_2019/transcript_assemblies/IDGE-Heliotropium_racemosum) | OneKP |
| Heliotropiaceae |  |  |  | *Euploca tenella* (Nutt.) Feuillet & Halse | N/A | N/A |  | [ERR2040522 = DIHD](https://datacommons.cyverse.org/browse/iplant/home/shared/commons_repo/curated/oneKP_capstone_2019/transcript_assemblies/DIHD-Heliotropium_tenellum) | OneKP |
| Heliotropiaceae |  |  |  | *Euploca tenuifolia* (R.Br.) Diane & Hilger | N/A | N/A |  | [ERR2040512= JWEY](https://datacommons.cyverse.org/browse/iplant/home/shared/commons_repo/curated/oneKP_capstone_2019/transcript_assemblies/JWEY-Heliotropium_tenuifolium) | OneKP |
| Heliotropiaceae |  |  |  | *Euploca texana* (I.M.Johnst.) M.W.Frohl. & M.W.Chase | N/A | N/A | CSWE | [ERR2040524=MDJK](https://datacommons.cyverse.org/browse/iplant/home/shared/commons_repo/curated/oneKP_capstone_2019/transcript_assemblies/MDJK-Heliotropium_texanum-2_samples_combined) | OneKP |
| Heliotropiaceae |  |  |  | *Heliotropium amplexicaule* Vahl | N/A | M. Dillon 8779 (F) | F59 | ERS23813217 | Present work |
| Heliotropiaceae |  |  |  | *Heliotropium arbainense* Fresen*.* | N/A | H. Förther 4049 (B); B100844184 | W0606 | ERS23813218 | Present work |
| Heliotropiaceae |  |  |  | *Heliotropium arborescens* L. | Peru; Moquegua | M. Weigend s.n. (BONN); BONN822 | W6214 | ERS23813219 | Present work |
| Heliotropiaceae |  |  |  | *Heliotropium arborescens* L. | Peru; Moquegua | M. Weigend & U. Baldárrago s.n. BONN | W4721 | ERS23813220 | Present work |
| Heliotropiaceae |  |  |  | *Heliotropium curassavicum* L. var. *curassavicum* | Peru | M. Weigend 2000/678 (B) | W2002 | ERS23813221 | Present work |
| Heliotropiaceae |  |  |  | *Heliotropium europaeum* L. | Germany; Bonn | s.n.; BONN568 | W6940 | ERS23813222 | Present work |
| Heliotropiaceae |  |  |  | *Heliotropium giessii* Friedr.-Holzh. | N/A | H.H. Hilger 93/03 (B); B100844677 | W0607 | ERS23813223 | Present work |
| Heliotropiaceae |  |  |  | *Heliotropium glutinosum* Phil. | Chile; Atacama | F. Luebert 1970 (SGO) | W1969 | ERS23813224 | Present work |
| Heliotropiaceae |  |  |  | *Heliotropium indicum* L. | India; Badlapur | N. R. Gupte NRG-02 | W4397 | ERS23813225 | Present work |
| Heliotropiaceae |  |  |  | *Heliotropium jaffiuelii* I.M.Johnst. | Chile; Atacama | N/A | W4928 | ERS23813226 | Present work |
| Heliotropiaceae |  |  |  | *Heliotropium messerschmidioides* Kuntze | N/A | M.W. Chase 6025 (K) |  | [ERR7621608](https://www.ebi.ac.uk/ena/browser/view/ERR7621608) | PAFTOL |
| Heliotropiaceae |  |  |  | *Heliotropium stenophyllum* Hook. & Arn. | Chile; Coquimbo | F. Luebert et al. (BONN) | W6929 | ERS23813227 | Present work |
| Heliotropiaceae |  |  |  | *Heliotropium verdcourtii* Craven | N/A | T. Clase 7613 (B) | W6947 | ERS23813228 | Present work |
| Heliotropiaceae |  |  |  | *Heliotropium verdcourtii* Craven | Dominican Republic; Barahona | M. Weigend 9916 (BONN) | W6578 | ERS23813229 | Present work |
| Heliotropiaceae |  |  |  | *Ixorhea tschudiana* Fenzl | Argentina | S.A. Renvoize et al. 3407 (K); K001275679 |  | [ERR7621523](https://www.ebi.ac.uk/ena/browser/view/ERR7621523) | PAFTOL |
| Heliotropiaceae |  |  |  | *Ixorhea tschudiana* Fenzl | Argentina; Salta | B. Schlumpberger BOS753 (BONN) | W6215 | ERS23813230 | Present work |
| Heliotropiaceae |  |  |  | *Myriopus poliochros* (Spreng.) Small | N/A | s.n. (BONN) | W4931 | ERS23813231 | Present work |
| Heliotropiaceae |  |  |  | *Myriopus stenophyllus* (Urb.) Feuillet | Dominican Republic; Azua | M. Weigend 9914 (BONN) | W6576 | ERS23813232 | Present work |
| Heliotropiaceae |  |  |  | *Myriopus volubilis* (L.) Small | Mexico | A.C. Sanders et al. 10813 (K); K001275693 |  | [ERR7621532](https://www.ebi.ac.uk/ena/browser/view/ERR7621532) | PAFTOL |
| Heliotropiaceae |  |  |  | *Nogalia drepanophylla* (Baker) Verdc. | Somalia | Fison, T. s.n. (K); K001275667 |  | [ERR7621515](https://www.ebi.ac.uk/ena/browser/view/ERR7621515) | PAFTOL |
| Hoplestigmaceae |  |  |  | *Hoplestigma klaineanum* Pierre | N/A | N/A |  | SRR16214534 | PRJNA767934 |
| Hoplestigmaceae |  |  |  | *Hoplestigma pierreanum* Gilg. | Cameroon; Mungo River | M. Cheeck 12325; K (ID: K001310892) | K23888 | ERS23813233 | Present work |
| Hydrophyllaceae |  |  |  | *Draperia systyla* (A.Gray) Torr. | USA; California | s.n.; BONN1752 | W5140 | ERS23813234 | Present work |
| Hydrophyllaceae |  |  |  | *Ellisia nyctelea* (L.) L. | USA; Illinois | G.N. Jones 24217 (K); K001275681 |  | [ERR7621525](https://www.ebi.ac.uk/ena/browser/view/ERR7621525) | PAFTOL |
| Hydrophyllaceae |  |  |  | *Emmenanthe penduliflora* Benth. | USA; California | s.n.; BONN2751 | W6199 | ERS23813235 | Present work |
| Hydrophyllaceae |  |  |  | *Eucrypta chrysanthemifolia* (Benth.) Greene | USA; California | s.n.; BONN990 | W6193 | ERS23813236 | Present work |
| Hydrophyllaceae |  |  |  | *Hydrophyllum canadense* L. | N/A | M.W. Chase 2548 (K) |  | [ERR7621599](https://www.ebi.ac.uk/ena/browser/view/ERR7621599) | PAFTOL |
| Hydrophyllaceae |  |  |  | *Hydrophyllum virginianum* L. | USA | s.n.; BONN1820 | W5141 | ERS23813237 | Present work |
| Hydrophyllaceae |  |  |  | *Nemophila menziesii* Hook. & Arn. | N/A | M.W. Chase 6550 (K) |  | [ERR7621604](https://www.ebi.ac.uk/ena/browser/view/ERR7621604) | PAFTOL |
| Hydrophyllaceae |  |  |  | *Phacelia bolanderi* A.Gray | USA | s.n; BONN3006 | W6203 | ERS23813238 | Present work |
| Hydrophyllaceae |  |  |  | *Pholistoma auritum* (Lindl.) Lilja | USA; California | s.n.; BONN2734 | W6198 | ERS23813239 | Present work |
| Hydrophyllaceae |  |  |  | *Pholistoma membranaceum* (Benth.) Constance | USA; California | R.R. Halse 6150 (K); K001275682 |  | [ERR7621526](https://www.ebi.ac.uk/ena/browser/view/ERR7621526) | PAFTOL |
| Hydrophyllaceae |  |  |  | *Romanzoffia californica* Greene | USA | s.n.; BONN3107 | W6202 | ERS23813240 | Present work |
| Lennoaceae |  |  |  | *Lennoa madreporoides* Lex. | N/A | N/A |  | [ERR2040528 = SMUR](https://datacommons.cyverse.org/browse/iplant/home/shared/commons_repo/curated/oneKP_capstone_2019/transcript_assemblies/SMUR-Lennoa_madreporoides) | OneKP |
| Lennoaceae |  |  |  | *Pholisma arenarium* Nutt. | USA; California | B. Lee & L. Grossenbacher 1 (BONN) | W6182 | ERS23813241 | Present work |
| Lennoaceae |  |  |  | *Pholisma arenarium* Nutt. | N/A | N/A |  | [ERR2040529 = HANM](https://treeoflife.kew.org/specimen-viewer/17995) | OneKP |
| Namaceae |  |  |  | *Eriodictyon (Turricula) parryi* (A. Gray) Green | USA; California | M.F. Spencer 1577 (K); K001275696 |  | [ERR7621535](https://www.ebi.ac.uk/ena/browser/view/ERR7621535) | PAFTOL |
| Namaceae |  |  |  | *Eriodictyon californicum* (Hook. & Arn.) Torr. | USA; California | s.n. 2663 (BONN) | W5662 | ERS23813242 | Present work |
| Namaceae |  |  |  | *Eriodictyon crassifolium* Benth. | USA; California | H. Ma L53 (FUS) |  | [SRR12034791](https://www.ebi.ac.uk/ena/browser/view/SRR12034791) | Zhang et al., 2020 |
| Namaceae |  |  |  | *Nama aretioides* (Hook. & Arn.) Brand | N/A | N/A | W5652 | ERS23813243 | Present work |
| Namaceae |  |  |  | *Nama densa* Lemmon | N/A | N/A | W5651 | ERS23813244 | Present work |
| Namaceae |  |  |  | *Nama dichotoma* (Ruiz & Pav.) Choisy | Bolivia; Oropeza | J.R.I. Wood 11873 (K); K000543033 |  | [ERR7621521](https://www.ebi.ac.uk/ena/browser/view/ERR7621521) | PAFTOL |
| Namaceae |  |  |  | *Nama dichotoma* (Ruiz & Pav.) Choisy | Chile; Tarapacá | F. Luebert et al. 3568 (BONN) | W6930 | ERS23813245 | Present work |
| Namaceae |  |  |  | *Nama rothrockii* A.Gray | USA | H.H. Hilger & M. Hofmann 1201 (B) | W0243 | ERS23813246 | Present work |
| Namaceae |  |  |  | *Wigandia (caracasana) urens* (Ruiz & Pav.) Kunth | N/A | s.n. 75-97/26 (MSB) | W6943 | ERS23813247 | Present work |
| Namaceae |  |  |  | *Wigandia brevistyla* Cornejo | Dominican Republic; Peravia | M. Weigend 9912 (BONN) | W6575 | ERS23813248 | Present work |
| Namaceae |  |  |  | *Wigandia ecuadorensis* Cornejo | Ecuador | Joßberger TJH-001 (BONN) | W5653 | ERS23813249 | Present work |
| Namaceae |  |  |  | *Wigandia urens* (Ruiz & Pav.) Kunth | N/A | 30082 (K) |  | [SRR7451086](https://www.ebi.ac.uk/ena/browser/view/SRR7451086) | PAFTOL |
| Rubiaceae |  |  |  | *Alibertia latifolia* (Benth.) K.Schum. | N/A | Persson et al. 635 (GB) |  | [ERR7622025](https://www.ebi.ac.uk/ena/browser/view/ERR7622025) | PAFTOL |
| Solanaceae |  |  |  | *Nicotiana rosulata* (S.Moore) Domin | N/A | M.W. Chase 68280 (K) |  | [ERR4180152](https://www.ebi.ac.uk/ena/browser/view/ERR4180152) | PAFTOL |
| Wellstediaceae |  |  |  | *Wellstedia dinteri* Pilg*.* | Namibia | H.H. Hilger s.n. (B) | W2670 | ERS23813250 | Present work |
| Wellstediaceae |  |  |  | *Wellstedia somalensis* Thulin & A.Johanss. | Somalia | M. Thulin et al. 10084 (UPPS) | W3004 | ERS23813251 | Present work |
