# Supplementary material for: An updated phylogeny of Boraginales based on the Angiosperms353 probe set: a roadmap for understanding morphological evolution
Source: Ann Bot. 2025 Apr 10;136(1):77–97. doi: 10.1093/aob/mcaf061 (PMC12401892; doi:10.1093/aob/mcaf061)
Supplement: mcaf061_suppl_Supplementary_Tables_S2 [file mcaf061_suppl_supplementary_tables_s2.docx]

TABLE S2. *Genes recovery statistics produced with the hybpiper_stats.py script available with HybPiper*

| Name | NumReads | ReadsMapped | PctOnTarget | GenesMapped | GenesWithContigs | GenesWithSeqs | GenesAt25pct | GenesAt50pct | GenesAt75pct | GenesAt150pct | ParalogWarningsLong | ParalogWarningsDepth | GenesWithoutStitchedContigs | GenesWithStitchedContigs | GenesWithStitchedContigsSkipped | GenesWithChimeraWarning |
| --- | --- | --- | --- | --- | --- | --- | --- | --- | --- | --- | --- | --- | --- | --- | --- | --- |
| SRR16214534_Hoplestigma_klaineanum | 27246758 | 275537 | 1 | 352 | 296 | 151 | 95 | 41 | 16 | 0 | 0 | 0 | 108 | 43 | 0 | 0 |
| Cynoglossum_viridiflorum_W6588 | 105374 | 24117 | 22.9 | 333 | 214 | 207 | 120 | 41 | 9 | 0 | 1 | 1 | 131 | 76 | 0 | 0 |
| ERR4180152_Nicotiana_rosulata | 111556 | 37392 | 33.5 | 344 | 223 | 221 | 183 | 125 | 72 | 0 | 18 | 27 | 151 | 70 | 0 | 0 |
| ERR5033622_Gentiana_cruciata | 1212918 | 61662 | 5.1 | 335 | 225 | 224 | 198 | 136 | 88 | 0 | 4 | 12 | 162 | 62 | 0 | 0 |
| SMUR_Lennoa_madreporoides | 16464378 | 52465 | 0.3 | 274 | 227 | 224 | 219 | 197 | 155 | 0 | 1 | 2 | 178 | 46 | 0 | 0 |
| ERR7621594_Chionocharis_hookeri | 1960644 | 103140 | 5.3 | 351 | 265 | 242 | 205 | 131 | 56 | 0 | 3 | 4 | 137 | 105 | 0 | 0 |
| ERR7599710_Plagiobothrys_plurisepalus | 457764 | 60065 | 13.1 | 337 | 257 | 251 | 217 | 136 | 67 | 0 | 2 | 3 | 153 | 98 | 0 | 0 |
| HANM_Pholisma_arenarium | 14680210 | 72469 | 0.5 | 290 | 256 | 253 | 244 | 228 | 207 | 0 | 0 | 1 | 201 | 52 | 0 | 0 |
| ERR7621509_Cynoglottis_barrelieri | 1717854 | 201007 | 11.7 | 348 | 294 | 255 | 193 | 86 | 29 | 0 | 3 | 3 | 124 | 131 | 0 | 0 |
| SRR11922382_Anchusa_officinalis | 23390766 | 300071 | 1.3 | 352 | 349 | 258 | 252 | 241 | 232 | 0 | 14 | 19 | 244 | 14 | 0 | 0 |
| DKFZ_Mertensia_paniculata | 23157920 | 78903 | 0.3 | 339 | 260 | 260 | 249 | 231 | 201 | 0 | 2 | 5 | 226 | 34 | 0 | 0 |
| ERR7599752_Embadium_jobnstonii | 473490 | 70997 | 15 | 339 | 272 | 262 | 225 | 131 | 63 | 0 | 1 | 3 | 139 | 123 | 0 | 0 |
| ERR7621607_Selkirkia_berteroi | 2991853 | 40120 | 1.3 | 329 | 268 | 262 | 188 | 76 | 24 | 0 | 2 | 2 | 140 | 122 | 0 | 0 |
| SRR12034773_Bourreria_succulenta | 21543798 | 342526 | 1.6 | 352 | 348 | 262 | 261 | 255 | 246 | 0 | 10 | 10 | 239 | 23 | 0 | 0 |
| CSWE_Heliotropium_texanum | 19741170 | 141778 | 0.7 | 350 | 263 | 263 | 256 | 239 | 219 | 0 | 16 | 22 | 234 | 29 | 0 | 0 |
| ERR7621596_Echiochilon_jugatum | 1446430 | 77599 | 5.4 | 351 | 283 | 264 | 217 | 142 | 64 | 0 | 3 | 3 | 137 | 127 | 0 | 0 |
| SRR7866831_Echium_wildpretii | 63125553 | 295455 | 0.5 | 339 | 265 | 265 | 242 | 155 | 96 | 0 | 1 | 1 | 197 | 68 | 0 | 0 |
| ERR7621511_Gastrocotyle_hispida | 1215420 | 123649 | 10.2 | 343 | 294 | 273 | 237 | 148 | 66 | 0 | 4 | 7 | 128 | 145 | 0 | 0 |
| SRR16690175_Echium_plantagineum | 6340101 | 4406 | 0.1 | 320 | 281 | 274 | 178 | 70 | 17 | 0 | 0 | 1 | 147 | 127 | 0 | 0 |
| SRR12034792_Codon_royenii | 23587888 | 353807 | 1.5 | 352 | 349 | 276 | 275 | 268 | 253 | 0 | 12 | 11 | 260 | 16 | 0 | 0 |
| W0856_Moritizia_lindenii | 150426 | 66513 | 44.2 | 345 | 278 | 276 | 245 | 169 | 98 | 0 | 10 | 13 | 186 | 90 | 0 | 0 |
| W6182_Pholisma_arenarium | 1292860 | 239081 | 18.5 | 349 | 285 | 278 | 276 | 256 | 215 | 0 | 4 | 9 | 79 | 199 | 0 | 0 |
| ERR7621534_Mattiastrum_formosum | 2227556 | 133715 | 6 | 351 | 307 | 279 | 236 | 145 | 68 | 0 | 2 | 4 | 151 | 128 | 0 | 0 |
| SRR12009669_Coldenia_procumbens | 15020654 | 202952 | 1.4 | 352 | 342 | 286 | 284 | 273 | 250 | 0 | 3 | 3 | 271 | 15 | 0 | 0 |
| ERR7621604_Nemophila_menziesii | 1762486 | 158624 | 9 | 347 | 304 | 289 | 276 | 199 | 115 | 0 | 5 | 7 | 196 | 93 | 0 | 0 |
| ERR7621526_Pholistoma_membranaceum | 2078932 | 248511 | 12 | 346 | 313 | 290 | 275 | 205 | 98 | 0 | 3 | 5 | 132 | 158 | 0 | 0 |
| ERR7621355_Arnebia_benthamii | 3194944 | 164844 | 5.2 | 348 | 312 | 296 | 261 | 182 | 106 | 0 | 4 | 8 | 144 | 152 | 0 | 0 |
| JWEY_Euploca_tenuifolia | 19869388 | 146566 | 0.7 | 349 | 297 | 297 | 290 | 278 | 251 | 0 | 5 | 10 | 262 | 35 | 0 | 0 |
| SRR12034787_Trichodesma_hildebrandtii | 22829280 | 379145 | 1.7 | 352 | 352 | 297 | 295 | 290 | 279 | 0 | 35 | 38 | 285 | 12 | 0 | 0 |
| ERR7621530_Oncaglossum_pringlei | 2962316 | 221425 | 7.5 | 351 | 318 | 298 | 259 | 162 | 78 | 0 | 3 | 6 | 146 | 152 | 0 | 0 |
| ERR7621531_Melanortocarya_obtusifolia | 4931006 | 1316478 | 26.7 | 336 | 304 | 300 | 237 | 123 | 45 | 0 | 3 | 5 | 113 | 187 | 0 | 0 |
| W6208_Hormuzakia_negevensis | 909512 | 80332 | 8.8 | 346 | 302 | 300 | 285 | 214 | 135 | 1 | 11 | 19 | 151 | 149 | 0 | 0 |
| EMAL_Ehretia_acuminata | 19491394 | 154362 | 0.8 | 347 | 304 | 303 | 301 | 294 | 272 | 0 | 7 | 10 | 264 | 39 | 0 | 0 |
| ERR7621591_Choriantha_popoviana | 1413234 | 184263 | 13 | 345 | 321 | 305 | 271 | 186 | 83 | 0 | 1 | 5 | 132 | 173 | 0 | 0 |
| ERR7621601_Lasiocaryum_munroi | 3870579 | 57169 | 1.5 | 329 | 306 | 305 | 251 | 136 | 56 | 0 | 2 | 2 | 126 | 179 | 0 | 0 |
| NIGS_Euploca_karwinskyi | 22808660 | 194229 | 0.9 | 349 | 305 | 305 | 300 | 295 | 274 | 0 | 10 | 18 | 265 | 40 | 0 | 0 |
| ABEH_Euploca_greggii | 24359762 | 188546 | 0.8 | 350 | 308 | 308 | 306 | 294 | 275 | 0 | 1 | 1 | 280 | 28 | 0 | 0 |
| ERR7621356_Brachybotrys_paridiformis | 2957768 | 443165 | 15 | 349 | 327 | 309 | 291 | 228 | 130 | 0 | 4 | 11 | 133 | 176 | 0 | 0 |
| SRR12034789_Rochelia_sessiliflora | 23755168 | 369601 | 1.6 | 352 | 348 | 309 | 305 | 301 | 284 | 0 | 16 | 20 | 296 | 13 | 0 | 0 |
| ERR7599751_Omphalolappula_concava | 2007726 | 365826 | 18.2 | 349 | 320 | 311 | 297 | 243 | 158 | 0 | 13 | 25 | 166 | 145 | 0 | 0 |
| ERR7621507_Antiotrema_dunnianum | 1849240 | 280857 | 15.2 | 347 | 329 | 311 | 287 | 198 | 93 | 0 | 7 | 11 | 108 | 203 | 0 | 0 |
| ERR7621518_Nonea_persica | 2391452 | 380219 | 15.9 | 349 | 333 | 311 | 291 | 193 | 97 | 0 | 4 | 8 | 112 | 199 | 0 | 0 |
| ERR7621593_Borago_officinalis | 2004860 | 214663 | 10.7 | 344 | 317 | 311 | 296 | 234 | 138 | 0 | 17 | 29 | 134 | 177 | 0 | 0 |
| IDGE_Euploca_racemosa | 24597622 | 208469 | 0.8 | 348 | 311 | 311 | 305 | 294 | 275 | 0 | 10 | 15 | 262 | 49 | 0 | 0 |
| ERR7621358_Aegonychon_purpurocaeruleum | 1942806 | 223053 | 11.5 | 348 | 326 | 312 | 289 | 218 | 123 | 0 | 7 | 13 | 132 | 180 | 0 | 0 |
| ERR7621522_Decalepidanthus_echioides | 1660664 | 283354 | 17.1 | 350 | 331 | 312 | 278 | 189 | 91 | 0 | 4 | 7 | 124 | 188 | 0 | 0 |
| DIHD_Euploca_tenella | 23205410 | 213085 | 0.9 | 348 | 313 | 313 | 308 | 298 | 280 | 0 | 21 | 36 | 280 | 33 | 0 | 0 |
| W2089_Moltkia_aurea | 609412 | 120111 | 19.7 | 346 | 315 | 313 | 303 | 252 | 162 | 0 | 8 | 18 | 154 | 159 | 0 | 0 |
| ERR7621508_Alkanna_orientalis | 1924568 | 308093 | 16 | 350 | 334 | 314 | 295 | 194 | 98 | 0 | 5 | 12 | 111 | 203 | 0 | 0 |
| ERR7621514_Cystostemon_heliocharis | 2627508 | 333788 | 12.7 | 348 | 330 | 315 | 291 | 224 | 122 | 0 | 8 | 17 | 130 | 185 | 0 | 0 |
| OUER_Euploca_convolvulacea | 19563714 | 151237 | 0.8 | 349 | 315 | 315 | 310 | 297 | 269 | 0 | 11 | 13 | 282 | 33 | 0 | 0 |
| SRR7451086_Wigandia_urens | 527102 | 78970 | 15 | 350 | 317 | 315 | 307 | 255 | 158 | 0 | 4 | 16 | 130 | 185 | 0 | 0 |
| ERR7621516_Lepechiniella_sarawschanica | 2478886 | 472868 | 19.1 | 344 | 330 | 316 | 301 | 239 | 135 | 0 | 4 | 11 | 121 | 195 | 0 | 0 |
| ERR7621599_Hydrophyllum_canadense | 2874988 | 160243 | 5.6 | 348 | 326 | 316 | 304 | 244 | 143 | 0 | 5 | 10 | 151 | 165 | 0 | 0 |
| ERR7621363_Lobostemon_fruticosus | 3458958 | 458705 | 13.3 | 347 | 333 | 317 | 297 | 238 | 157 | 0 | 6 | 11 | 134 | 184 | 0 | 0 |
| ERR7621364_Paramoltkia_doerfleri | 2845900 | 352797 | 12.4 | 349 | 329 | 317 | 303 | 235 | 145 | 0 | 10 | 17 | 138 | 179 | 0 | 0 |
| ERR7621520_Cynoglossum_amplifolium | 4541816 | 666098 | 14.7 | 352 | 339 | 317 | 296 | 214 | 108 | 0 | 5 | 9 | 107 | 210 | 0 | 0 |
| ERR7621361_Oreocarya_virgata | 1734920 | 280335 | 16.2 | 347 | 330 | 319 | 298 | 227 | 137 | 0 | 3 | 7 | 154 | 165 | 0 | 0 |
| ERR7621529_Neatostema_apulum | 2118800 | 198291 | 9.4 | 352 | 332 | 319 | 289 | 225 | 130 | 0 | 13 | 23 | 117 | 202 | 0 | 0 |
| ERR7621592_Ivanjohnstonia_jaunsariensis | 2167470 | 440580 | 20.3 | 348 | 332 | 319 | 300 | 213 | 115 | 0 | 3 | 9 | 119 | 200 | 0 | 0 |
| ERR7621605_Pardoglossum_cheirifolium | 3426136 | 384609 | 11.2 | 352 | 330 | 319 | 309 | 249 | 162 | 0 | 10 | 22 | 135 | 184 | 0 | 0 |
| ERR5970511_Cordia_myxa | 2685368 | 253587 | 9.4 | 351 | 329 | 321 | 299 | 236 | 145 | 0 | 17 | 39 | 156 | 165 | 0 | 0 |
| ERR7621365_Pulmonaria_rubra | 2024060 | 254206 | 12.6 | 347 | 329 | 321 | 307 | 234 | 142 | 0 | 10 | 15 | 167 | 154 | 0 | 0 |
| IPPG_Euploca_filiformis | 26554464 | 215949 | 0.8 | 350 | 322 | 321 | 320 | 300 | 285 | 0 | 2 | 3 | 294 | 27 | 0 | 0 |
| SRR12034791_Eriodictyon_crassifolium | 24355446 | 444827 | 1.8 | 351 | 350 | 321 | 320 | 318 | 314 | 0 | 37 | 38 | 306 | 15 | 0 | 0 |
| W4759_Moritzia_dasyantha | 805876 | 134186 | 16.7 | 349 | 323 | 322 | 310 | 263 | 178 | 0 | 14 | 30 | 140 | 182 | 0 | 0 |
| ERR7621357_Brunnera_macrophylla | 2967988 | 343351 | 11.6 | 347 | 336 | 323 | 310 | 245 | 165 | 0 | 8 | 24 | 133 | 190 | 0 | 0 |
| ERR7621533_Stephanocaryum_dschagastanicum | 2382534 | 216626 | 9.1 | 351 | 336 | 323 | 299 | 239 | 154 | 0 | 5 | 12 | 108 | 215 | 0 | 0 |
| W6198_Pholistoma_auritum | 883704 | 160062 | 18.1 | 348 | 325 | 324 | 319 | 277 | 217 | 0 | 13 | 19 | 204 | 120 | 0 | 0 |
| ERR7621525_Ellisia_nyctelea | 1114072 | 135099 | 12.1 | 351 | 331 | 325 | 306 | 256 | 141 | 0 | 4 | 6 | 123 | 202 | 0 | 0 |
| ERR7621527_Oreocarya_paradoxa | 2473790 | 382136 | 15.4 | 350 | 335 | 325 | 302 | 232 | 139 | 0 | 5 | 7 | 139 | 186 | 0 | 0 |
| ERR7621600_Lappula_squarrosa | 2911808 | 651290 | 22.4 | 345 | 335 | 325 | 314 | 273 | 179 | 1 | 12 | 33 | 133 | 192 | 0 | 0 |
| MZOB_Euploca_mendocina | 24743260 | 202092 | 0.8 | 350 | 326 | 325 | 322 | 311 | 290 | 0 | 1 | 1 | 292 | 33 | 0 | 0 |
| SRR6374711_Trigonotis_peduncularis | 29031018 | 285311 | 1 | 352 | 335 | 325 | 322 | 312 | 283 | 0 | 10 | 14 | 296 | 29 | 0 | 0 |
| ERR7621519_Solenanthus_circinnatus | 2784242 | 434957 | 15.6 | 351 | 340 | 326 | 316 | 238 | 129 | 0 | 7 | 12 | 123 | 203 | 0 | 0 |
| ERR7621523_Ixhorea_tschudianna | 1033688 | 118730 | 11.5 | 350 | 332 | 326 | 304 | 243 | 153 | 1 | 0 | 3 | 103 | 223 | 0 | 0 |
| SRR11994239_Amsinckia_grandiflora | 104382914 | 653859 | 0.6 | 352 | 351 | 326 | 314 | 271 | 208 | 0 | 3 | 11 | 93 | 233 | 0 | 0 |
| ERR7621359_Caccinia_strigosa | 2988698 | 533553 | 17.9 | 348 | 340 | 327 | 319 | 269 | 179 | 0 | 11 | 26 | 120 | 207 | 0 | 0 |
| ERR7621609_Trichodesma_scottii | 2804616 | 399123 | 14.2 | 348 | 335 | 327 | 321 | 266 | 179 | 0 | 12 | 28 | 140 | 187 | 0 | 0 |
| W4757_Thaumatocaryon_tetraquetrum | 1875730 | 245434 | 13.1 | 349 | 332 | 327 | 319 | 283 | 208 | 0 | 17 | 47 | 134 | 193 | 0 | 0 |
| ERR7621362_Lindelofia_anchusoides | 2572174 | 382090 | 14.9 | 350 | 337 | 328 | 314 | 244 | 146 | 0 | 10 | 18 | 144 | 184 | 0 | 0 |
| SRR6374694_Bothriospermum_chinense | 29719448 | 258733 | 0.9 | 352 | 340 | 328 | 328 | 322 | 308 | 0 | 17 | 20 | 296 | 32 | 0 | 0 |
| ERR7621512_Paracaryum_platycalyx | 3789036 | 625275 | 16.5 | 351 | 344 | 330 | 319 | 256 | 152 | 0 | 8 | 17 | 116 | 214 | 0 | 0 |
| ERR7621603_Myosotis_arvensis | 3738514 | 750929 | 20.1 | 350 | 340 | 330 | 320 | 272 | 194 | 1 | 12 | 32 | 119 | 211 | 0 | 0 |
| W2335_Trachelanthus_cerinthoides | 1164350 | 246771 | 21.2 | 348 | 330 | 330 | 325 | 280 | 197 | 0 | 12 | 26 | 140 | 190 | 0 | 0 |
| ERR5970513_Ehretia_microphylla | 574676 | 210064 | 36.6 | 349 | 336 | 331 | 301 | 226 | 112 | 0 | 4 | 7 | 112 | 219 | 0 | 0 |
| W4950_Pontechium_maculatum | 1197522 | 264456 | 22.1 | 350 | 331 | 331 | 323 | 288 | 217 | 2 | 15 | 31 | 148 | 183 | 0 | 0 |
| ERR4180157_Codon_schenckii | 1930516 | 185980 | 9.6 | 349 | 337 | 332 | 315 | 270 | 186 | 0 | 5 | 11 | 133 | 199 | 0 | 0 |
| SRR7027846_Anchusa_capensis | 59949996 | 680801 | 1.1 | 352 | 350 | 333 | 333 | 332 | 323 | 0 | 38 | 44 | 310 | 23 | 0 | 0 |
| W0588_Eritrichium_nanum | 1491484 | 619811 | 41.6 | 349 | 335 | 333 | 332 | 306 | 238 | 0 | 24 | 43 | 151 | 182 | 0 | 0 |
| W2294_Amblynotus_rupestris | 1428882 | 413133 | 28.9 | 349 | 334 | 333 | 331 | 303 | 239 | 0 | 11 | 26 | 158 | 175 | 0 | 0 |
| W2834_Adelinia_grande | 1179628 | 158266 | 13.4 | 349 | 334 | 333 | 326 | 278 | 192 | 0 | 9 | 21 | 152 | 181 | 0 | 0 |
| ERR7621510_Asperugo_procumbens | 4365998 | 1280404 | 29.3 | 350 | 348 | 334 | 330 | 291 | 217 | 0 | 14 | 32 | 130 | 204 | 0 | 0 |
| ERR7621515_Nogalia_drepanophylla | 3933866 | 392714 | 10 | 351 | 341 | 334 | 325 | 260 | 156 | 0 | 1 | 2 | 96 | 238 | 0 | 0 |
| Maharanga_hookeri_W1011 | 545892 | 191351 | 35.1 | 351 | 336 | 334 | 318 | 238 | 134 | 0 | 8 | 35 | 91 | 243 | 0 | 0 |
| W0654_Solenanthus_appenninus | 1643936 | 518788 | 31.6 | 351 | 337 | 334 | 332 | 298 | 237 | 0 | 15 | 28 | 135 | 199 | 0 | 0 |
| W4056_Arnebia_decumbens | 1294038 | 306174 | 23.7 | 350 | 336 | 334 | 331 | 294 | 205 | 1 | 15 | 37 | 136 | 198 | 0 | 0 |
| W6189_Cordia_macleodii | 865428 | 160198 | 18.5 | 351 | 337 | 334 | 328 | 293 | 213 | 0 | 31 | 72 | 107 | 227 | 0 | 0 |
| W6352_Craniospermum_subvillosum | 1088222 | 297040 | 27.3 | 351 | 337 | 334 | 330 | 294 | 227 | 0 | 13 | 38 | 151 | 183 | 0 | 0 |
| Onosma_gigantea_W5090 | 1828524 | 430719 | 23.6 | 351 | 339 | 335 | 331 | 296 | 219 | 0 | 9 | 40 | 63 | 272 | 0 | 0 |
| W2542_Echiochilon_fruticosum | 1446738 | 329705 | 22.8 | 349 | 337 | 335 | 331 | 291 | 214 | 0 | 13 | 29 | 125 | 210 | 0 | 0 |
| W4043_Craniospermum_echioides | 1132648 | 221927 | 19.6 | 350 | 336 | 335 | 330 | 287 | 218 | 0 | 14 | 35 | 151 | 184 | 0 | 0 |
| W4273_Trigonotis_formosana_var_elevatovenosa | 2256208 | 529347 | 23.5 | 348 | 336 | 335 | 331 | 296 | 218 | 0 | 20 | 59 | 129 | 206 | 0 | 0 |
| W4959_Moltkiopsis_ciliata | 1409954 | 410517 | 29.1 | 351 | 338 | 335 | 331 | 305 | 233 | 0 | 8 | 23 | 154 | 181 | 0 | 0 |
| W6197_Alkanna_mathioli | 1182276 | 342051 | 28.9 | 348 | 336 | 335 | 326 | 301 | 240 | 0 | 10 | 32 | 150 | 185 | 0 | 0 |
| W1686_Glandora_oleifolia | 1504010 | 502338 | 33.4 | 349 | 336 | 336 | 334 | 304 | 235 | 1 | 14 | 31 | 162 | 174 | 0 | 0 |
| W2064_Huynhia_pulchra | 1290164 | 372769 | 28.9 | 349 | 336 | 336 | 331 | 298 | 224 | 1 | 16 | 37 | 127 | 209 | 0 | 0 |
| W2603_Lasiocaryum_trichocarpum | 1758284 | 485666 | 27.6 | 350 | 336 | 336 | 335 | 311 | 252 | 0 | 9 | 24 | 160 | 176 | 0 | 0 |
| W6354_Myosotis_rehsteineri | 2524908 | 1003825 | 39.8 | 348 | 336 | 336 | 332 | 308 | 249 | 0 | 15 | 28 | 160 | 176 | 0 | 0 |
| ERR2040513_Euploca_calcicola | 22572978 | 147505 | 0.7 | 349 | 337 | 337 | 330 | 302 | 242 | 1 | 10 | 19 | 212 | 125 | 0 | 0 |
| ERR7621608_Heliotropium_messerschmidioi | 2695916 | 351684 | 13 | 350 | 341 | 337 | 332 | 294 | 212 | 0 | 2 | 8 | 121 | 216 | 0 | 0 |
| W1255_Mattiastrum_lithospermifolium | 2100938 | 744965 | 35.5 | 351 | 339 | 337 | 334 | 311 | 244 | 0 | 13 | 28 | 144 | 193 | 0 | 0 |
| W2626_Pentaglottis_sempervirens | 1220442 | 283190 | 23.2 | 349 | 338 | 337 | 332 | 290 | 226 | 0 | 11 | 39 | 121 | 216 | 0 | 0 |
| W5312_Rindera_lanata | 1600106 | 449756 | 28.1 | 350 | 339 | 337 | 334 | 302 | 233 | 1 | 13 | 32 | 142 | 195 | 0 | 0 |
| W5448_Afrotysonia_glochidiata | 1235928 | 311437 | 25.2 | 348 | 337 | 337 | 334 | 302 | 231 | 0 | 11 | 19 | 142 | 195 | 0 | 0 |
| W1602_Buglossoides_arvensis | 1698264 | 788294 | 46.4 | 350 | 338 | 338 | 335 | 308 | 238 | 0 | 20 | 57 | 135 | 203 | 0 | 0 |
| W2702_Decalepidanthus_primuloides | 1563052 | 452677 | 29 | 349 | 339 | 338 | 333 | 305 | 233 | 1 | 11 | 23 | 147 | 191 | 0 | 0 |
| W2981_Microparacaryum_intermedium | 2643344 | 891970 | 33.7 | 351 | 340 | 338 | 336 | 308 | 244 | 0 | 14 | 27 | 159 | 179 | 0 | 0 |
| Omphalodes_nitida_W6901 | 5412964 | 2226898 | 41.1 | 352 | 344 | 339 | 339 | 323 | 271 | 1 | 13 | 30 | 141 | 198 | 0 | 0 |
| W0409_Lithospermum_officinale | 2336068 | 937344 | 40.1 | 350 | 340 | 339 | 333 | 315 | 248 | 0 | 12 | 22 | 159 | 180 | 0 | 0 |
| W0480_Hackelia_deflexa | 2236742 | 898646 | 40.2 | 349 | 340 | 339 | 334 | 316 | 250 | 0 | 17 | 29 | 155 | 184 | 0 | 0 |
| W0675_Heterocaryum_szovitsianum | 2658350 | 1171927 | 44.1 | 350 | 343 | 339 | 337 | 313 | 260 | 0 | 24 | 54 | 146 | 193 | 0 | 0 |
| W2115_Paracynoglossum_asperrimum | 1597108 | 575989 | 36.1 | 350 | 342 | 339 | 333 | 301 | 226 | 0 | 13 | 29 | 145 | 194 | 0 | 0 |
| W2623_Myosotidium_hortensia | 2497010 | 880659 | 35.3 | 349 | 339 | 339 | 336 | 313 | 263 | 1 | 47 | 98 | 125 | 214 | 0 | 0 |
| W2833_Dasynotus_daubenmirei | 2415998 | 849449 | 35.2 | 350 | 341 | 339 | 337 | 308 | 252 | 0 | 10 | 26 | 145 | 194 | 0 | 0 |
| W4981_Cerinthe_major | 2144470 | 792726 | 37 | 348 | 339 | 339 | 336 | 303 | 250 | 0 | 14 | 27 | 176 | 163 | 0 | 0 |
| W4990_Adelocaryum_coelestinum | 1775364 | 543329 | 30.6 | 349 | 341 | 339 | 335 | 303 | 232 | 0 | 14 | 28 | 145 | 194 | 0 | 0 |
| W6206_Plagiobothrys_chorisianus_var_hickmanii | 3550380 | 1450190 | 40.8 | 349 | 339 | 339 | 335 | 312 | 265 | 0 | 9 | 23 | 157 | 182 | 0 | 0 |
| W6209_Cryptantha_clevelandii | 4161112 | 1429489 | 34.4 | 349 | 339 | 339 | 335 | 316 | 255 | 0 | 15 | 28 | 151 | 188 | 0 | 0 |
| W6211_Oreocarya_humilis | 2174518 | 641384 | 29.5 | 350 | 339 | 339 | 336 | 306 | 252 | 1 | 8 | 19 | 136 | 203 | 0 | 0 |
| Omphalodes_cappadocica_W6359 | 5081686 | 2028533 | 39.9 | 352 | 344 | 340 | 340 | 322 | 273 | 0 | 16 | 38 | 143 | 197 | 0 | 0 |
| W1320_Paracaryum_rugulosum | 1678338 | 585171 | 34.9 | 350 | 342 | 340 | 339 | 305 | 239 | 0 | 17 | 29 | 142 | 198 | 0 | 0 |
| W2664_Onosma_alborosea | 2812286 | 936595 | 33.3 | 350 | 340 | 340 | 336 | 310 | 244 | 0 | 12 | 26 | 135 | 205 | 0 | 0 |
| W2690_Cryptantha_micrantha | 3720014 | 1504032 | 40.4 | 349 | 342 | 340 | 338 | 317 | 257 | 0 | 7 | 14 | 159 | 181 | 0 | 0 |
| W4082_Pulmonaria_angustifolia | 2715240 | 648513 | 23.9 | 352 | 341 | 340 | 338 | 311 | 241 | 0 | 14 | 33 | 127 | 213 | 0 | 0 |
| W4983_Halacsya_sendtneri | 1458858 | 519282 | 35.6 | 349 | 341 | 340 | 330 | 306 | 235 | 0 | 34 | 110 | 147 | 193 | 0 | 0 |
| W5141_Hydrophyllum_virginianum | 1412180 | 235540 | 16.7 | 350 | 341 | 340 | 336 | 307 | 242 | 0 | 7 | 24 | 143 | 197 | 0 | 0 |
| W6185_Lappula_consanguinea | 3149368 | 1287639 | 40.9 | 351 | 342 | 340 | 335 | 317 | 260 | 0 | 31 | 84 | 136 | 204 | 0 | 0 |
| W6195_Amsinckia_spectabilis | 4171380 | 1903572 | 45.6 | 349 | 343 | 340 | 339 | 316 | 255 | 0 | 13 | 62 | 117 | 223 | 0 | 0 |
| Eritrichium_tschuktschorum_W6953 | 2530696 | 907433 | 35.9 | 351 | 344 | 341 | 337 | 309 | 255 | 1 | 20 | 72 | 101 | 240 | 0 | 0 |
| ERR7599654_Coldenia_procumbens | 8529186 | 2128147 | 25 | 350 | 345 | 341 | 340 | 317 | 233 | 0 | 1 | 3 | 105 | 236 | 0 | 0 |
| ERR7621513_Lepidocordia_punctata | 2467296 | 773932 | 31.4 | 351 | 347 | 341 | 334 | 304 | 217 | 1 | 24 | 103 | 67 | 274 | 0 | 0 |
| W2063_Echium_vulgare | 2662198 | 1071610 | 40.3 | 349 | 345 | 341 | 337 | 308 | 251 | 0 | 11 | 33 | 146 | 195 | 0 | 0 |
| W2620_Memoremea_scorpioides | 2515786 | 893654 | 35.5 | 349 | 341 | 341 | 339 | 312 | 254 | 0 | 10 | 24 | 161 | 180 | 0 | 0 |
| W2970_Microcaryum_pygmaeum | 2288394 | 804260 | 35.1 | 350 | 343 | 341 | 338 | 317 | 263 | 0 | 39 | 107 | 148 | 193 | 0 | 0 |
| W4307_Bothriospermum_zeylanicum | 3842740 | 1111462 | 28.9 | 350 | 343 | 341 | 337 | 319 | 257 | 1 | 14 | 31 | 144 | 197 | 0 | 0 |
| W5355_Lindelofia_longiflora | 1927350 | 563268 | 29.2 | 350 | 343 | 341 | 337 | 304 | 234 | 0 | 19 | 28 | 141 | 200 | 0 | 0 |
| W6210_Cryptantha_micromeres | 3111594 | 1438630 | 46.2 | 350 | 341 | 341 | 337 | 313 | 253 | 1 | 8 | 25 | 155 | 186 | 0 | 0 |
| W6353_Myosotis_scorpiodes | 3214508 | 1389818 | 43.2 | 350 | 342 | 341 | 337 | 314 | 250 | 1 | 17 | 43 | 152 | 189 | 0 | 0 |
| W6362_Trichodesma_indicum | 2833678 | 1268074 | 44.8 | 349 | 342 | 341 | 339 | 319 | 278 | 0 | 21 | 40 | 139 | 202 | 0 | 0 |
| ERR7621532_Myriopus_volubilis | 4240324 | 966492 | 22.8 | 352 | 348 | 342 | 338 | 312 | 243 | 0 | 30 | 86 | 109 | 233 | 0 | 0 |
| Microula_diffusa_W6945 | 2700444 | 935898 | 34.7 | 351 | 344 | 342 | 341 | 317 | 242 | 0 | 11 | 41 | 105 | 237 | 0 | 0 |
| W0629_Symphytum_tuberosum | 1438046 | 583571 | 40.6 | 350 | 342 | 342 | 336 | 308 | 238 | 2 | 19 | 65 | 119 | 223 | 0 | 0 |
| W0666_Trachystemon_orientale | 1590522 | 594527 | 37.4 | 348 | 342 | 342 | 338 | 310 | 240 | 0 | 11 | 36 | 120 | 222 | 0 | 0 |
| W2295_Anoplocaryum_compressum | 2234854 | 716471 | 32.1 | 351 | 344 | 342 | 341 | 310 | 256 | 0 | 15 | 42 | 141 | 201 | 0 | 0 |
| W2728_Mertensia_ciliata | 2487688 | 847242 | 34.1 | 349 | 342 | 342 | 341 | 316 | 260 | 1 | 15 | 34 | 147 | 195 | 0 | 0 |
| W2729_Nonea_lutea | 2351980 | 738716 | 31.4 | 349 | 343 | 342 | 338 | 319 | 266 | 0 | 6 | 15 | 171 | 171 | 0 | 0 |
| W2905_Mertensia_lanceolata | 1860138 | 576837 | 31 | 350 | 342 | 342 | 341 | 306 | 252 | 0 | 16 | 35 | 148 | 194 | 0 | 0 |
| Cynoglossum_germanicum_W6939 | 4942256 | 1343315 | 27.2 | 351 | 346 | 343 | 342 | 318 | 265 | 0 | 23 | 50 | 135 | 208 | 0 | 0 |
| Cynoglossum_zeylanicum_W5229 | 2400632 | 812952 | 33.9 | 351 | 347 | 343 | 338 | 309 | 238 | 0 | 14 | 32 | 115 | 228 | 0 | 0 |
| ERR7621521_Nama_dichotoma | 4008562 | 1144013 | 28.5 | 350 | 347 | 343 | 339 | 318 | 229 | 0 | 29 | 82 | 90 | 253 | 0 | 0 |
| Microula_spathulata_W4278 | 5583848 | 2243415 | 40.2 | 352 | 344 | 343 | 340 | 327 | 259 | 1 | 12 | 31 | 134 | 209 | 0 | 0 |
| Onosma_echioides_W5044 | 3346804 | 934031 | 27.9 | 351 | 343 | 343 | 337 | 320 | 257 | 0 | 18 | 43 | 121 | 222 | 0 | 0 |
| Cynoglossum_officinale_W5234 | 5966042 | 1633712 | 27.4 | 350 | 346 | 344 | 342 | 319 | 268 | 1 | 19 | 39 | 146 | 198 | 0 | 0 |
| ERR7622025_Alibertia_latifolia | 2699712 | 1027092 | 38 | 352 | 349 | 344 | 334 | 292 | 212 | 1 | 2 | 8 | 95 | 249 | 0 | 0 |
| Omphalodes_verna_W6942 | 3796874 | 1628711 | 42.9 | 352 | 345 | 344 | 340 | 321 | 266 | 0 | 16 | 52 | 109 | 235 | 0 | 0 |
| Varronia_bahamensis_W6204 | 1466188 | 325862 | 22.2 | 351 | 345 | 344 | 333 | 301 | 222 | 0 | 13 | 36 | 106 | 238 | 0 | 0 |
| W1301_Rochelia_cancellata | 4569444 | 1883793 | 41.2 | 350 | 344 | 344 | 341 | 319 | 268 | 0 | 11 | 26 | 179 | 165 | 0 | 0 |
| W2691_Harpagonella_palmeri | 5536776 | 2660399 | 48 | 350 | 345 | 344 | 340 | 324 | 270 | 0 | 15 | 26 | 163 | 181 | 0 | 0 |
| W3004_Wellstedia_somalensis | 2463398 | 838570 | 34 | 350 | 345 | 344 | 341 | 317 | 269 | 1 | 14 | 30 | 143 | 201 | 0 | 0 |
| W4272_Cynoglossum_australe | 2920650 | 778417 | 26.7 | 351 | 344 | 344 | 340 | 306 | 242 | 0 | 11 | 33 | 139 | 205 | 0 | 0 |
| W6216_Varronia_cylindrostachya | 1800830 | 612507 | 34 | 350 | 344 | 344 | 341 | 325 | 285 | 1 | 20 | 72 | 146 | 198 | 0 | 0 |
| Eritrichium_tianschanicum_W6954 | 3262112 | 1247755 | 38.2 | 352 | 348 | 345 | 339 | 314 | 253 | 0 | 12 | 36 | 123 | 222 | 0 | 0 |
| ERR7621535_Turricula_parryi | 1908532 | 477881 | 25 | 351 | 349 | 345 | 334 | 300 | 212 | 0 | 9 | 26 | 76 | 269 | 0 | 0 |
| Myosotis_macrosperma_W6937 | 5043488 | 2284278 | 45.3 | 352 | 349 | 345 | 343 | 320 | 272 | 1 | 49 | 178 | 97 | 248 | 0 | 0 |
| Podonosma_orientalis_W5104 | 3614532 | 1159249 | 32.1 | 352 | 346 | 345 | 341 | 320 | 264 | 1 | 9 | 29 | 119 | 226 | 0 | 0 |
| Selkirkia_pauciflora_W6934 | 3684582 | 1172922 | 31.8 | 352 | 347 | 345 | 341 | 320 | 258 | 1 | 34 | 91 | 120 | 225 | 0 | 0 |
| Suchtelenia_calycina_W6952 | 7148632 | 3228864 | 45.2 | 352 | 347 | 345 | 343 | 322 | 276 | 1 | 23 | 41 | 149 | 196 | 0 | 0 |
| W5517_Tiquilia_litoralis | 2309776 | 902328 | 39.1 | 349 | 345 | 345 | 345 | 336 | 295 | 0 | 63 | 159 | 155 | 190 | 0 | 0 |
| W6207_Rochelia_disperma | 3724950 | 1563232 | 42 | 350 | 345 | 345 | 341 | 320 | 266 | 0 | 11 | 25 | 169 | 176 | 0 | 0 |
| Maharanga_sinica_W4665 | 7998760 | 3419046 | 42.7 | 351 | 348 | 346 | 343 | 324 | 271 | 1 | 11 | 28 | 135 | 211 | 0 | 0 |
| SRR11934229_Varronia_rupicola | 81777322 | 1572611 | 1.9 | 352 | 351 | 346 | 346 | 346 | 338 | 1 | 30 | 48 | 305 | 41 | 0 | 0 |
| W2280_Tiquilia_dichotoma | 2664162 | 1253962 | 47.1 | 349 | 347 | 346 | 345 | 337 | 307 | 0 | 71 | 166 | 159 | 187 | 0 | 0 |
| W4634_Ogastemma_pusillum | 5426938 | 2112348 | 38.9 | 352 | 347 | 346 | 346 | 330 | 285 | 1 | 50 | 155 | 116 | 230 | 0 | 0 |
| Adelocaryum_nebulicola_W6931 | 2273216 | 681669 | 30 | 351 | 349 | 347 | 342 | 308 | 224 | 0 | 12 | 27 | 102 | 245 | 0 | 0 |
| Brandelia_erythraea_W6941 | 1986844 | 676695 | 34.1 | 351 | 349 | 347 | 340 | 304 | 237 | 0 | 14 | 35 | 92 | 255 | 0 | 0 |
| ERR7619226_Ipomoea_nil | 3036762 | 1620750 | 53.4 | 351 | 349 | 347 | 340 | 310 | 236 | 0 | 8 | 17 | 128 | 219 | 0 | 0 |
| ERR7621598_Halgania_anagalloides | 2569064 | 462792 | 18 | 351 | 348 | 347 | 343 | 311 | 236 | 0 | 2 | 12 | 99 | 248 | 0 | 0 |
| Heliotropium_giessii_W0607 | 1810152 | 627796 | 34.7 | 351 | 348 | 347 | 346 | 332 | 277 | 0 | 6 | 44 | 65 | 282 | 0 | 0 |
| Omphalodes_linifolia_W6936 | 4286400 | 1842177 | 43 | 352 | 348 | 347 | 347 | 329 | 286 | 1 | 53 | 175 | 102 | 245 | 0 | 0 |
| Tiquilia_hispidissima_W0886 | 3657154 | 1669504 | 45.7 | 351 | 349 | 347 | 346 | 339 | 303 | 0 | 7 | 27 | 142 | 205 | 0 | 0 |
| Varronia_jeremiensis_W6582 | 7089634 | 2179427 | 30.7 | 352 | 347 | 347 | 347 | 335 | 298 | 1 | 14 | 25 | 177 | 170 | 0 | 0 |
| Varronia_serrata_W6583 | 5779242 | 1650336 | 28.6 | 351 | 348 | 347 | 346 | 334 | 294 | 1 | 12 | 23 | 174 | 173 | 0 | 0 |
| W2670_Wellstedia_dinteri | 3381056 | 1075309 | 31.8 | 350 | 347 | 347 | 345 | 331 | 284 | 0 | 20 | 44 | 175 | 172 | 0 | 0 |
| W4931_Myriopus_poliochros | 2445046 | 1091589 | 44.6 | 351 | 348 | 347 | 347 | 344 | 302 | 1 | 43 | 138 | 123 | 224 | 0 | 0 |
| W6188_Coldenia_procumbens | 3791974 | 2001881 | 52.8 | 350 | 347 | 347 | 347 | 341 | 311 | 1 | 2 | 6 | 149 | 198 | 0 | 0 |
| W6364_Codon_schenkii | 2262534 | 992722 | 43.9 | 350 | 348 | 347 | 344 | 332 | 274 | 0 | 2 | 12 | 88 | 259 | 0 | 0 |
| Bourreria_quirosii_W6964 | 6623284 | 3118707 | 47.1 | 352 | 350 | 348 | 348 | 344 | 313 | 1 | 11 | 22 | 102 | 246 | 0 | 0 |
| Caccinia_actinobole_W6944 | 4328898 | 1681458 | 38.8 | 351 | 349 | 348 | 345 | 332 | 268 | 0 | 23 | 67 | 89 | 259 | 0 | 0 |
| Cordia_dichotoma_W4400 | 3637618 | 642735 | 17.7 | 351 | 348 | 348 | 348 | 333 | 288 | 1 | 44 | 151 | 96 | 252 | 0 | 0 |
| Cordia_sebestena_W6190 | 5558402 | 2578893 | 46.4 | 352 | 348 | 348 | 348 | 344 | 315 | 0 | 7 | 25 | 101 | 247 | 0 | 0 |
| Heliotropium_indicum_W4397 | 1198184 | 535185 | 44.7 | 351 | 348 | 348 | 347 | 332 | 293 | 0 | 3 | 7 | 127 | 221 | 0 | 0 |
| Keraunea_brasiliensis_W6910 | 3513290 | 651985 | 18.6 | 351 | 348 | 348 | 346 | 341 | 291 | 0 | 12 | 26 | 72 | 276 | 0 | 0 |
| Keraunea_capixaba_W6911 | 2758866 | 509048 | 18.5 | 351 | 348 | 348 | 347 | 337 | 280 | 0 | 9 | 21 | 76 | 272 | 0 | 0 |
| Keraunea_confusa_W6912 | 3538572 | 720234 | 20.4 | 352 | 348 | 348 | 346 | 340 | 295 | 1 | 11 | 27 | 73 | 275 | 0 | 0 |
| Microula_pustulosa_W6946 | 4965164 | 1856111 | 37.4 | 352 | 352 | 348 | 345 | 326 | 264 | 0 | 17 | 36 | 128 | 220 | 0 | 0 |
| Myosotis_laxa_W6938 | 3668440 | 1581554 | 43.1 | 352 | 349 | 348 | 345 | 318 | 256 | 1 | 19 | 113 | 76 | 272 | 0 | 0 |
| Tiquilia_tacnensis_W6935 | 10775972 | 5628907 | 52.2 | 351 | 350 | 348 | 346 | 344 | 318 | 0 | 91 | 207 | 153 | 195 | 0 | 0 |
| Varronia_salviifolia_W6584 | 7610276 | 3480607 | 45.7 | 352 | 349 | 348 | 348 | 344 | 320 | 0 | 8 | 55 | 76 | 272 | 0 | 0 |
| W0979_Cordia_sonorae | 1855978 | 696083 | 37.5 | 350 | 348 | 348 | 348 | 337 | 296 | 0 | 2 | 19 | 104 | 244 | 0 | 0 |
| W2002_Heliotropium_curassavicum | 990390 | 372903 | 37.7 | 350 | 348 | 348 | 346 | 334 | 272 | 0 | 3 | 3 | 109 | 239 | 0 | 0 |
| W5140_Draperia_systyla | 1737588 | 699150 | 40.2 | 350 | 348 | 348 | 347 | 336 | 305 | 0 | 5 | 25 | 110 | 238 | 0 | 0 |
| W6193_Eucrypta_chrysanthemifolia | 2338384 | 1122375 | 48 | 350 | 348 | 348 | 346 | 338 | 304 | 0 | 6 | 16 | 139 | 209 | 0 | 0 |
| Bourreria_huanita_W6963 | 8702900 | 3701095 | 42.5 | 352 | 349 | 349 | 349 | 346 | 315 | 0 | 15 | 60 | 75 | 274 | 0 | 0 |
| Cordia_sinensis_W6187 | 4521212 | 1032999 | 22.8 | 351 | 349 | 349 | 348 | 340 | 296 | 1 | 46 | 155 | 92 | 257 | 0 | 0 |
| Heliotropium_verdcourtii_W6947 | 4377090 | 774471 | 17.7 | 352 | 351 | 349 | 346 | 331 | 280 | 0 | 5 | 23 | 91 | 258 | 0 | 0 |
| Keraunea_velutina_W6914 | 1912250 | 320843 | 16.8 | 352 | 349 | 349 | 347 | 324 | 253 | 1 | 14 | 43 | 66 | 283 | 0 | 0 |
| Myriopus_stenophyllus_W6576 | 8106292 | 3631170 | 44.8 | 352 | 350 | 349 | 347 | 341 | 317 | 2 | 59 | 136 | 139 | 210 | 0 | 0 |
| Nama_aretioides_W5652 | 7604894 | 3224024 | 42.4 | 352 | 350 | 349 | 348 | 342 | 317 | 0 | 15 | 77 | 78 | 271 | 0 | 0 |
| Nama_densa_W5651 | 6032068 | 2532513 | 42 | 351 | 350 | 349 | 348 | 339 | 311 | 0 | 9 | 22 | 114 | 235 | 0 | 0 |
| Nama_dichotomum_W6930 | 2991782 | 1372231 | 45.9 | 350 | 349 | 349 | 348 | 336 | 298 | 0 | 8 | 19 | 113 | 236 | 0 | 0 |
| Rochefortia_lundellii_W6965 | 8675418 | 4355368 | 50.2 | 352 | 349 | 349 | 349 | 346 | 321 | 1 | 80 | 217 | 117 | 232 | 0 | 0 |
| W1969_Heliotropium_glutinosum | 1859870 | 742703 | 39.9 | 350 | 349 | 349 | 348 | 340 | 296 | 0 | 7 | 8 | 112 | 237 | 0 | 0 |
| W4135_Euploca_humifusa | 4766142 | 1787482 | 37.5 | 352 | 350 | 349 | 349 | 345 | 315 | 0 | 77 | 204 | 122 | 227 | 0 | 0 |
| W6199_Emmenanthe_penduliflora | 2654750 | 1278967 | 48.2 | 351 | 350 | 349 | 349 | 338 | 308 | 0 | 37 | 129 | 113 | 236 | 0 | 0 |
| W6202_Romanzoffia_californica | 3059448 | 1330642 | 43.5 | 350 | 350 | 349 | 348 | 343 | 308 | 1 | 7 | 21 | 139 | 210 | 0 | 0 |
| W6203_Phacelia_bolanderi | 2337630 | 947576 | 40.5 | 350 | 349 | 349 | 348 | 336 | 302 | 0 | 8 | 16 | 151 | 198 | 0 | 0 |
| W6215_Ixorhea_tschudiana | 4184850 | 2048415 | 48.9 | 350 | 349 | 349 | 349 | 341 | 311 | 0 | 1 | 11 | 111 | 238 | 0 | 0 |
| W6367_Ehretia_dicksonii | 1540310 | 628496 | 40.8 | 350 | 349 | 349 | 349 | 339 | 307 | 0 | 4 | 27 | 85 | 264 | 0 | 0 |
| Cordia_decandra_F74 | 6803578 | 2375153 | 34.9 | 351 | 351 | 350 | 349 | 344 | 320 | 0 | 15 | 54 | 93 | 257 | 0 | 0 |
| Ehretia_tinifolia_W6577 | 5909038 | 2898036 | 49 | 351 | 350 | 350 | 350 | 349 | 322 | 0 | 11 | 35 | 103 | 247 | 0 | 0 |
| ERR7621366_Rotula_aquatica | 3147136 | 862479 | 27.4 | 350 | 350 | 350 | 348 | 326 | 258 | 0 | 7 | 15 | 121 | 229 | 0 | 0 |
| Halgania_cyanea_W6567 | 3595334 | 1825785 | 50.8 | 352 | 350 | 350 | 350 | 343 | 318 | 0 | 5 | 30 | 99 | 251 | 0 | 0 |
| Halgania_erecta_W6572 | 8074502 | 3368706 | 41.7 | 352 | 350 | 350 | 350 | 346 | 324 | 0 | 15 | 87 | 73 | 277 | 0 | 0 |
| Heliotropium_arbaniense_W0606 | 3190870 | 1094616 | 34.3 | 351 | 351 | 350 | 348 | 334 | 286 | 1 | 9 | 41 | 81 | 269 | 0 | 0 |
| Heliotropium_corymbosum_W4721 | 6788254 | 2991864 | 44.1 | 351 | 350 | 350 | 350 | 345 | 323 | 0 | 8 | 28 | 104 | 246 | 0 | 0 |
| Heliotropium_europaeum_W6940 | 5953056 | 2302691 | 38.7 | 351 | 350 | 350 | 349 | 342 | 302 | 0 | 36 | 142 | 82 | 268 | 0 | 0 |
| Heliotropium_stenophyllum_W6929 | 4019550 | 1571807 | 39.1 | 351 | 350 | 350 | 349 | 339 | 305 | 0 | 6 | 12 | 99 | 251 | 0 | 0 |
| Hoplestigma_pierreanum_K23888 | 8070156 | 3470939 | 43 | 352 | 350 | 350 | 350 | 347 | 323 | 0 | 7 | 26 | 84 | 266 | 0 | 0 |
| Lepidocordia_williamsii_W6366 | 6796092 | 3536473 | 52 | 351 | 350 | 350 | 350 | 348 | 324 | 1 | 66 | 214 | 102 | 248 | 0 | 0 |
| Myosotis_ramosissima_W6356 | 12909598 | 5582145 | 43.2 | 352 | 351 | 350 | 348 | 334 | 290 | 1 | 33 | 165 | 97 | 253 | 0 | 0 |
| Rochefortia_oblongata_W6923 | 3211384 | 1599571 | 49.8 | 351 | 350 | 350 | 350 | 342 | 311 | 1 | 55 | 199 | 68 | 282 | 0 | 0 |
| Rochefortia_stellata_W6921 | 8533972 | 4445229 | 52.1 | 352 | 350 | 350 | 350 | 348 | 325 | 1 | 72 | 239 | 95 | 255 | 0 | 0 |
| Tournefortia_bicolor_W6578 | 3606038 | 1530392 | 42.4 | 351 | 350 | 350 | 350 | 340 | 298 | 0 | 5 | 13 | 129 | 221 | 0 | 0 |
| W0243_Nama_rothrockii | 2478148 | 1107616 | 44.7 | 350 | 350 | 350 | 350 | 343 | 317 | 0 | 20 | 48 | 80 | 270 | 0 | 0 |
| W4928_Heliotropium_jaffuelii | 1779344 | 589947 | 33.2 | 351 | 350 | 350 | 349 | 338 | 288 | 1 | 6 | 9 | 123 | 227 | 0 | 0 |
| W5653_Wigandia_ecuadorensis | 2418412 | 1098774 | 45.4 | 350 | 350 | 350 | 350 | 341 | 309 | 0 | 10 | 39 | 83 | 267 | 0 | 0 |
| W5662_Eriodictyon_californicum | 3280396 | 1431713 | 43.6 | 350 | 350 | 350 | 350 | 344 | 321 | 0 | 16 | 63 | 72 | 278 | 0 | 0 |
| W6212_Bourreria_ovata | 3763236 | 1805070 | 48 | 350 | 350 | 350 | 350 | 343 | 310 | 0 | 6 | 20 | 106 | 244 | 0 | 0 |
| W6214_Heliotropium_arborescens | 3201378 | 1386300 | 43.3 | 351 | 350 | 350 | 350 | 343 | 308 | 0 | 6 | 15 | 109 | 241 | 0 | 0 |
| Wigandia_brevistyla_W6575 | 8325056 | 3845152 | 46.2 | 351 | 351 | 350 | 350 | 346 | 320 | 0 | 20 | 49 | 85 | 265 | 0 | 0 |
| Wigandia_caracasana_W6943 | 2684772 | 1047596 | 39 | 351 | 351 | 350 | 349 | 342 | 307 | 0 | 12 | 51 | 45 | 305 | 0 | 0 |
| Halgania_solanacea_W6565 | 7124434 | 3020052 | 42.4 | 352 | 351 | 351 | 351 | 346 | 319 | 0 | 14 | 51 | 100 | 251 | 0 | 0 |
| Heliotropium_amplexicaule_F59 | 6421204 | 1921126 | 29.9 | 351 | 351 | 351 | 350 | 342 | 306 | 0 | 12 | 28 | 116 | 235 | 0 | 0 |
| Rochefortia_cubensis_W6928 | 5365730 | 2779644 | 51.8 | 351 | 351 | 351 | 350 | 345 | 320 | 1 | 63 | 223 | 98 | 253 | 0 | 0 |
