# Supplementary material for: An updated phylogeny of Boraginales based on the Angiosperms353 probe set: a roadmap for understanding morphological evolution
Source: Ann Bot. 2025 Apr 10;136(1):77–97. doi: 10.1093/aob/mcaf061 (PMC12401892; doi:10.1093/aob/mcaf061)
Supplement: mcaf061_suppl_Supplementary_Tables_S3 [file mcaf061_suppl_supplementary_tables_s3.docx]

TABLE S3. *Scanner and reconstruction settings.*

| Taxon | Scanned Material | Source voltage (kV) | Source current (µA) | Exposure time (ms) | Frame averaging | Camera binning | Voxel size (µm) | 360^o^ Rotation | Images | Figures |
| --- | --- | --- | --- | --- | --- | --- | --- | --- | --- | --- |
| *Ogastemma pusillum* | mature fruit | 55 | 181 | 1700 | 6 | 1x1 | 1.5 | yes | 1714 | 5A |
| *Codon schenckii* | anthetic gynoecium | 50 | 200 | 569 | 4 | 2x2 | 9 | yes | 1800 | 5B |
| *Nama jamaicensis* | mature fruit | 40 | 200 | 520 | 7 | 1x1 | 3.69 | yes | 1565 | 5C |
| *Wigandia ecuadorensis* | post-anthetic gynoecium | 60 | 133 | 500 | 7 | 1x1 | 11.71 | yes | 1894 | 5D |
| *Emmenanthe penduliflora* | mature fruit | 45 | 165 | 444 | 6 | 2x2 | 7 | no | 961 | 5E |
| *Phacelia malvifolia* | mature fruit | 45 | 165 | 1166 | 3 | 2x2 | 3 | no | 940 | 5F |
| *Coldenia procumbens* | mature fruit | 55 | 181 | 1600 | 6 | 1x1 | 2.2 | yes | 1715 | 5G |
| *Bourreria succulenta* | mature fruit | 60 | 166 | 1500 | 5 | 2x2 | 6.99 | yes | 923 | 5H |
| *Pholisma arenarium* | mature fruit | 45 | 165 | 1300 | 5 | 2x2 | 4.39 | yes | 1894 | 5I |
| *Varronia cylindristachya* | mature fruit | 45 | 177 | 500 | 7 | 1x1 | 5.67 | yes | 1565 | 5J |
| *Heliotropium arbainense* | mature fruit | 65 | 153 | 680 | 7 | 1x1 | 2.99 | yes | 1565 | 5K |
| *Tournefortia* undulata | mature fruit | 65 | 153 | 680 | 7 | 1x1 | 2.99 | yes | 1714 | 5L |
